# Antiplasmodial potential of phytochemicals from *Citrus aurantifolia* peels: a comprehensive in vitro and in silico study

**DOI:** 10.1186/s13065-024-01162-x

**Published:** 2024-03-30

**Authors:** Abeer H. Elmaidomy, Usama Ramadan Abdelmohsen, Ahmed M. Sayed, Faisal H. Altemani, Naseh A. Algehainy, Denisa Soost, Thomas Paululat, Gerhard Bringmann, Esraa M. Mohamed

**Affiliations:** 1 Department of Pharmacognosy, Faculty of Pharmacy, Beni-Suef University, Beni-Suef, 62514 Egypt; 2 Department of Pharmacognosy, Faculty of Pharmacy, Minia University, Minia, 61519 Egypt; 3 Department of Pharmacognosy, Faculty of Pharmacy, Deraya University, Minia, 61111 Egypt; 4 Department of Pharmacognosy, Faculty of Pharmacy, Nahda University, Beni-Suef, 62513 Egypt; 5 Department of Medical Laboratory Technology, Faculty of Applied Medical Sciences, University of Tabuk, Tabuk, 71491 Saudi Arabia; 6 Department of Chemistry and Biology, University of Siegen, Adolf-Reichwein-Str. 2, 57068 Siegen, Germany; 7 Institute of Organic Chemistry, University of Würzburg, Am Hubland, 97074 Würzburg, Germany; 8 Department of Pharmacognosy, Faculty of Pharmacy, MUST, Giza, 12566 Egypt

**Keywords:** *Citrus*, Key lime, Network pharmacology, In silico analysis

## Abstract

Phytochemical investigation of Key lime (*Citrus aurantifolia* L., F. Rutaceae) peels afforded six metabolites, known as methyl isolimonate acetate (1), limonin (2), luteolin (3), 3ˋ-hydroxygenkwanin (4), myricetin (5), and europetin (6). The structures of the isolated compounds were assigned by 1D NMR. In the case of limonin (2), further 1- and 2D NMR experiments were done to further confirm the structure of this most active metabolite. The antiplasmodial properties of the obtained compounds against the pathogenic NF54 strain of *Plasmodium falciparum* were assessed in vitro. According to antiplasmodial screening, only limonin (2), luteolin (3), and myricetin (5) were effective (IC_50_ values of 0.2, 3.4, and 5.9 µM, respectively). We explored the antiplasmodial potential of phytochemicals from *C. aurantifolia* peels using a stepwise in silico-based analysis. We first identified the unique proteins of *P. falciparum* that have no homolog in the human proteome, and then performed inverse docking, Δ*G*_Binding_ calculation, and molecular dynamics simulation to predict the binding affinity and stability of the isolated compounds with these proteins. We found that limonin (2), luteolin (3), and myricetin (5) could interact with 20S a proteasome, choline kinase, and phosphocholine cytidylyltransferase, respectively, which are important enzymes for the survival and growth of the parasite. According to our findings, phytochemicals from *C. aurantifolia* peels can be considered as potential leads for the development of new safe and effective antiplasmodial agents.

**Supplementary Information:**

The online version contains supplementary material available at 10.1186/s13065-024-01162-x.

## Introduction

Malaria continues to be a serious threat to public health, particularly in tropical and subtropical countries. It is a disease caused by parasites belonging to several species of the genus *Plasmodium*, including *Plasmodium falciparum, P. malariae, P. ovale, P. vivax, and P. knowlesi*, which are spread via the bite of an infected female *Anopheles mosquito* [1]. The most virulent of these species is *P. falciparum*, it is responsible for the greatest levels of morbidity and mortality. It is, moreover, the most common species in sub-Saharan Africa (SSA), having the greatest rate of malaria infections and fatalities worldwide [1]. Globally in 2018, there were an estimated 249 million malaria cases and 608 000 malaria deaths in 85 countries. The World Health Organization WHO African Region carries a disproportionately high share of the global malaria burden. In 2018, the Region was home to 94% of malaria cases (233 million) and 95% (580 000) of malaria deaths. Children under 5 accounted for about 80% of all malaria deaths in the Region [1].

Chemotherapy can be used to treat malaria, although the parasites can be highly resistant against many of the medications. In 2006, Cambodia reported the first case of artemisinin resistance, which afterwards spread to the majority of South-East Asia [2]. Another key worry is the safety of chemoprophylaxis; for example, primaquine, atovaquone, and doxycycline are not recommended for use in young children and pregnant women [3]. All these issues make the development of new malaria medications necessary.

Natural substances, such as plant products, have significantly contributed to the development of new drugs during the past 50 years and have been of benefit to the pharmaceutical industry [4]. For instance, numerous pharmacological classes that were first developed based on active chemicals from plant sources have enabled therapies for different infectious diseases, cancer, and other debilitating diseases caused by metabolic abnormalities [4]. Additionally, the first components of anti-malarial chemotherapy, quinine and artemisinin, as well as their synthetic analogs, were also derived from plant sources. Many people in malaria-endemic regions, particularly in Africa, turn to herbal remedies as the primary form of therapy [5]. The expense of conventional medications, accessibility, perceived efficacy, lack of adverse effects, and faith in traditional remedies are only some few of the frequent factors that influence people’s preferences [6].

*Citrus aurantifolia* was imported to North Africa, Europe, and other parts of the world after being native to tropical and subtropical regions of Southeast Asia, including India and China [7]. It is also known as Key lime [8]. *C. aurantifolia* contains active phytochemical substances such as flavonoids including apigenin, hesperetin, kaempferol, nobiletin, quercetin, naringenin, and rutin [9, 10], as well as flavones [11], flavanones [12, 13] triterpenoids [14], and limonoids [15]. The traditional uses of *C. aurantifolia* from several literature reviews are described as antibacterial [16], antidiabetic [17], antifungal [18], antihypertensive [19], anti-inflammatory [20], anti-hyperlipidemic [21], anti-parasitic [22], and antiplatelet activities [11]. It is furthermore used for the treatment of cardiovascular [23], hepatic [24], osteoporosis [25], and urolithiasis disorders [26], and acts as a fertility promoter [27]. Moreover, it can be used for insecticidal activity [28].

Herein, we subjected the peels of *C. aurantifolia* to a stepwise chromatographic isolation to get information about the major phytochemicals in this waste product. Subsequently, we investigated whether the identified phytochemicals have antiplasmodial pharmacological effects, then subjected them to a stepwise in silico-based analysis, which was initiated by a comprehensive inverse docking and ended by several MDS experiments. The potential of the bioactive phytochemicals derived from the *C. aurantifolia* peels sheds light on the high capacity of waste products from edible fruits as a huge potential reservoir for health-promoting agents.

## Results and discussion

### Phytochemical Investigation of *Citrus aurantifolia* peels

**Fig. 1:**
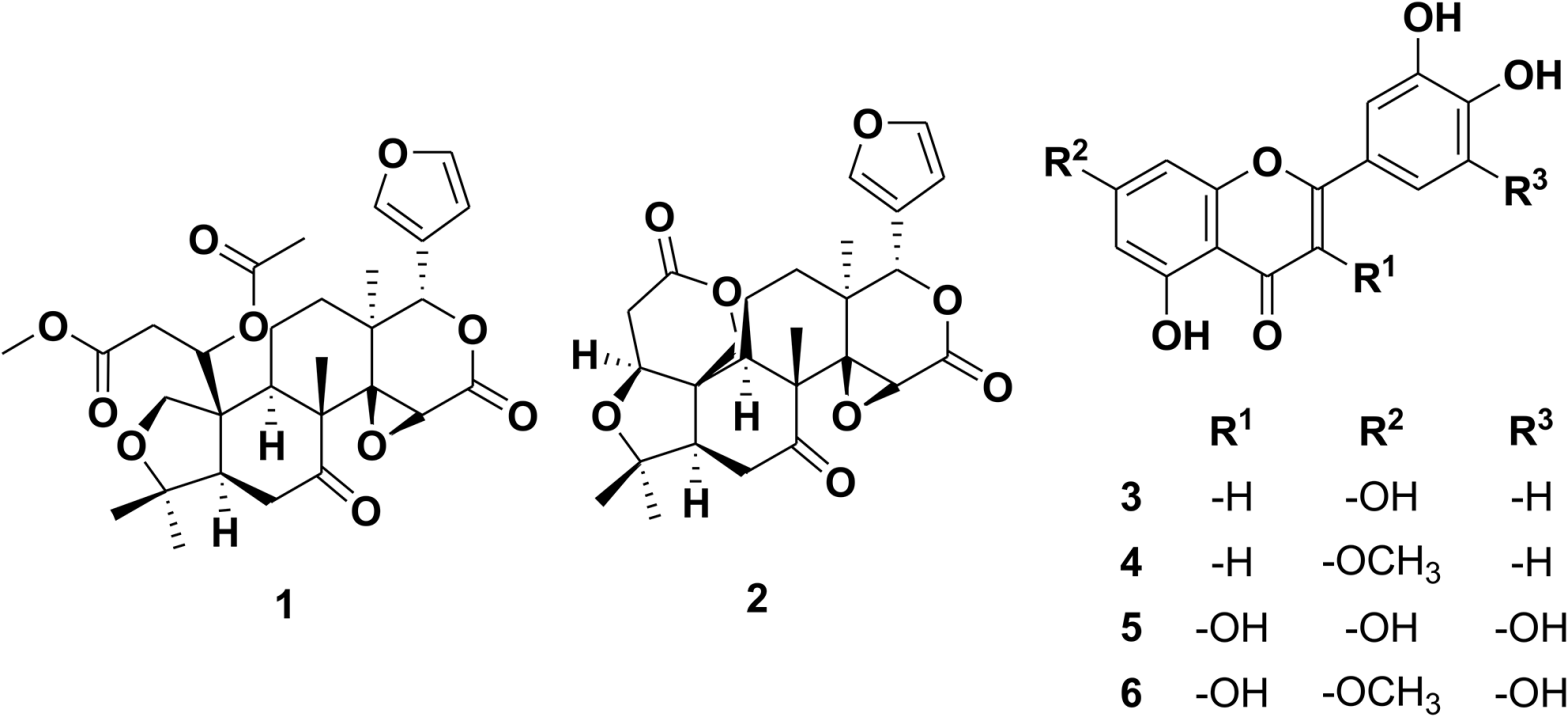
Structures of compounds isolated from *Citrus aurantifolia* peels

Based on the physicochemical and chromatographic plots, the spectral investigations from UV, ^1^H, and DEPT-Q NMR, besides correlations with the literature and some authoritative samples, the crude ethanolic extract of *C. aurantifolia* peels were found to contain the known compounds methyl isolimonate acetate 1 [29, 30], limonin 2 [31], luteolin 3 [32], 3ˋ-hydroxygenkwanin 4 [33], myricetin 5 [34], and europetin 6 [35] (Fig. 1). The identified compound **1** was isolated herein for the first time (Figures S1-15, Fig. 1). Compound **2**, as the most active of the substances investigated in this study, was analyzed more in depth by a series of 1- and 2D NMR experiments.

### Antiplasmodial activities

Malaria remains a significant public health challenge in regions of the world where it is endemic. Natural products have played a key role in the control and treatment of malaria. Quinine, a component of the bark of the *Cinchona* tree, was first used to treat malaria from as early as the 1600s and remained the mainstay for malaria treatment until the 1920s when the more effective synthetic derivative chloroquine became available [36]. Since the failure of chloroquine began in the 1960s [37], several other synthetic naturally derived compounds have been developed for clinical use including atovaquone, amodiaquine, and mefloquine [38, 39]. Since the early 2000s, the sesquiterpene lactone artemisinin, which is derived from the sweet wormwood *Artemisia annua*, or synthetic derivatives (e.g. dihydroartemisinin, artemether and artesunate), have been used as the foundation combination drug partner in the highly effective artemisinin combination therapies. However, according to WHO, malaria is staging a comeback, in part due to the development of drug resistance. Therefore, the continued search for new anti-malarial agents remains an urgent priority.

Due to structural characteristics such as several stereocenters, flexible conformations, presence of heteroatoms, natural products are more likely than synthetic compounds to have multiple targets and/or new targets [40]. Researchers investigating natural products as potential anti-malarial drugs need to incorporate the screening of the compounds for the interaction with newly identified druggable targets, in order to identify hits/leads. Therefore, the continued exploration of natural products as antiplasmodial agents is of great scientific interest [40]. Plant-derived antiplasmodial compounds organized according to plant families covering the literature from 1990 to 2000 have been reviewed [41]. Similarly, reviews categorizing antiplasmodial compounds isolated from plants according to phytochemical classes have been conducted by Bero et al. (2005–2011) [42, 43], Nogueira and Lopes (2009–2010) [44], Tajuddeen and Van Heerden (2010–2017) [40], and Wright (2000–2010) [45]. Finally, reviews covering antiplasmodial marine natural products up to 2009 have been published by Laurent and Pietra, and Fattorusso and Taglialatela-Scafati [46, 47].

In this study, the pathogenic NF54 strain of *P. falciparum* was used to test the antiplasmodial properties of the recovered components **1**–**6** isolated from the ethanolic extract of *C. aurantifolia* peels. According to the antiplasmodial screening, the compounds limonin [2], luteolin [3], and myricetin [5] were efficacious (IC_50_ 0.2, 3.4, and 5.9 µM, respectively), while the others were inert (> 50 µM).

### Bioinformatics-based analysis

#### PPI network of the cancer related targets and KEGG-based enrichment analysis

To identify possible druggable targets for malaria disease, we first collected all previously reported malaria-derived proteins from a number of relevant databases. Using the following keywords: “malaria” and “plasmodium“, we searched in the Toxicogenomics (https://ctdbase.org/) and PlasmoDB (plasmodb.org) databases, and in the the previously published literature. As a result, 3373 proteins relevant to the malaria disease were retrieved (Table S1).

To select the unique targets that are non-homolog to the human host, the collected protein targets in the previous screening were subjected to a screening based on a comparative sequence analysis (see the section Materials and methods) against the human proteome. As a result, 182 proteins (Table S2) were found to be non-homolog to the human host, and hence, they could serve as excellent candidates for in silico drug screening.

In a second step, we used the Cytoscape software to generate a protein-protein interaction (PPI) network from the 181 chosen proteins.

**Fig. 2:**
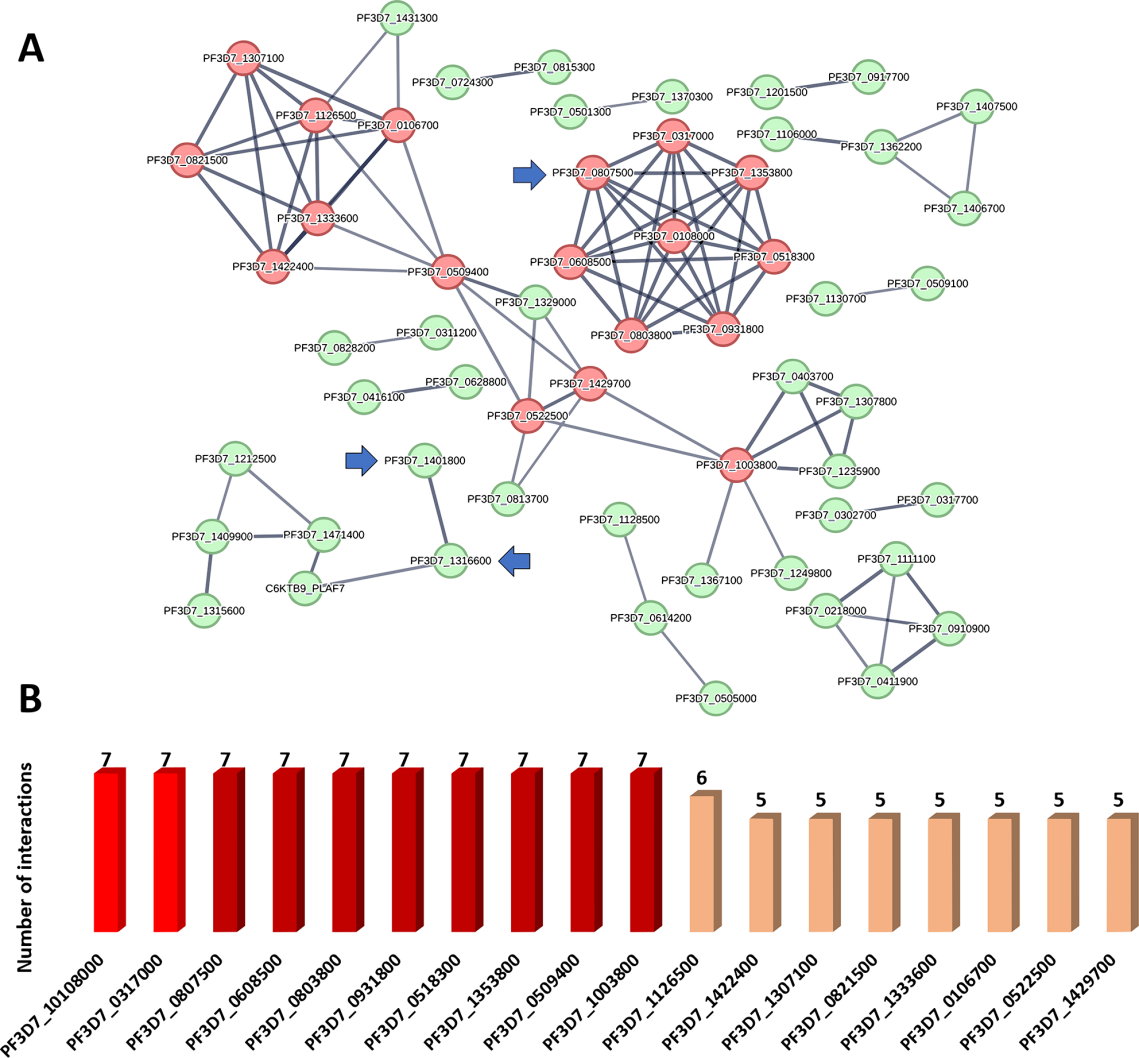
(**A**): *P. falciparum* PPI network. This network consists of 56 nodes and 82 edges with an average node degree of 1.05. The top-interacting nodes are colored red (32.1%, 18 proteins of all interacting nodes, i.e., hub protein). The selection criterion for the top-interacting proteins was a minimum of five interactions for each protein. (**B**): The top interacting nodes (i.e., hub nodes arranged by their degree value). Blue arrows represent the proteins predicted as probable targets for compound **2** (limonin) investigated in the presented study. The thickness of the lines (i.e., edges) represents the degree of confidence (i.e., the strength of data support)

Figure 2 depicts the network properties of the generated PPI, which had an intermediate degree of connectivity (82 edges between 56 nodes; a mean node degree = 1.05; and a local clustering coefficient value = 0.281). The remaining 125 proteins in the list of selected 182 proteins (Table S2) did not demonstrate any links and were thus eliminated from the PPI network.

Therapeutic strategies for diseases including malaria may have a better chance of success if they focus on proteins with high degrees of interaction, as these are typically the most important and relevant molecular targets (i.e., hub proteins or genes) in a given network [48]. Therefore, we highlighted the top 10% (i.e. 18 proteins) interacting proteins (i.e., hub proteins) in the generated network and ranked them by their degree value.

**Fig. 3:**
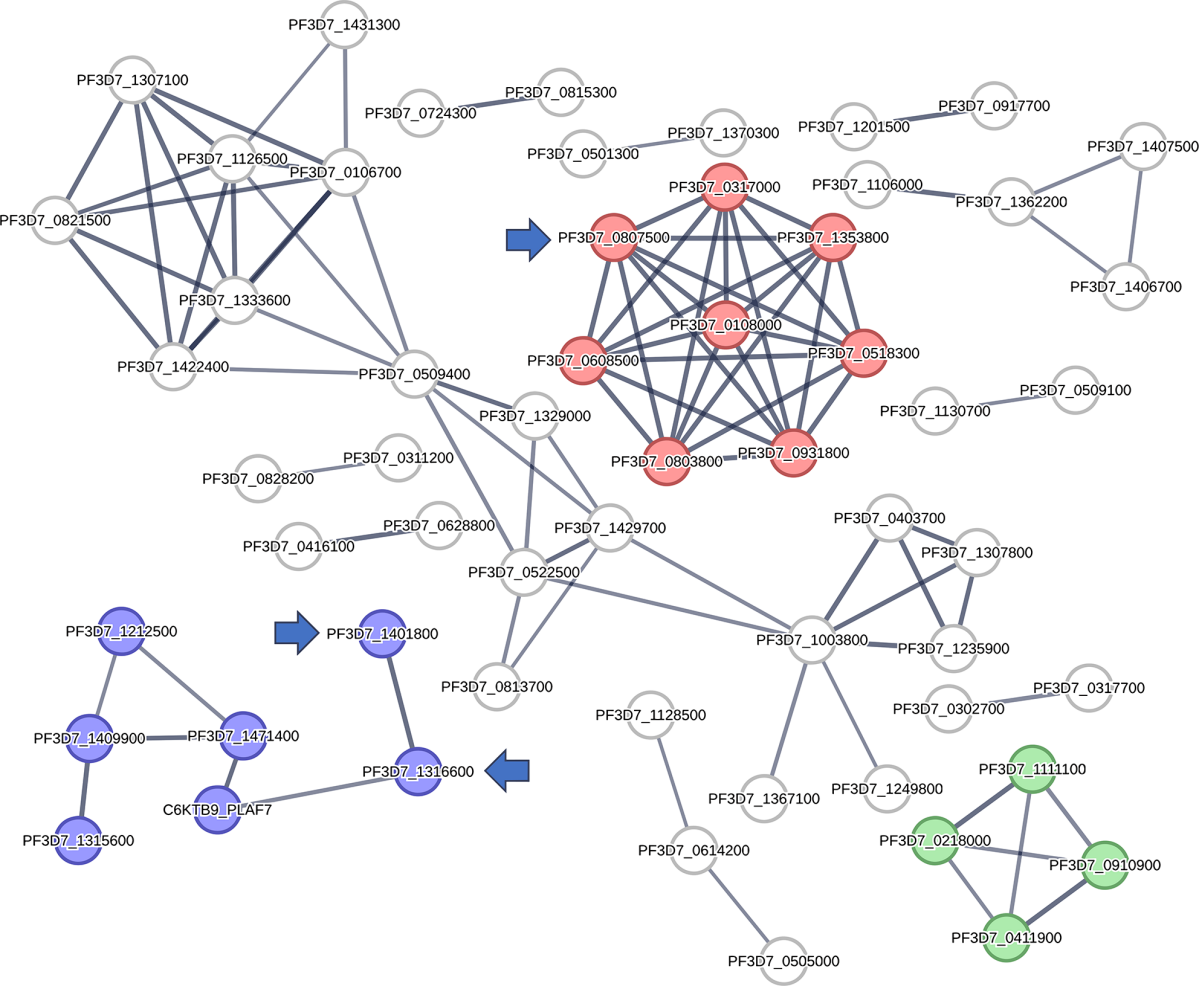
The four key signaling pathways in *P. falciparum* according to the presented PPI networking. Red nodes represent the ubiquitin–proteasome system in *P. falciparum*; violet nodes exemplify the glycerophospholipid metabolism in *P. falciparum*; (iii) DNA replication in *P. falciparum*; and (iv) intracellular anatomical structure in *P. falciparum*. Blue arrows highlight the proteins predicted as probable targets for limonin (**2**) investigated in the presented study. The thickness of the lines (i.e., edges) represents the degree of confidence (i.e., the strength of data support)

Furthermore, we classified the proteins in the current network based on their involvement in the numerous signaling pathways linked to malaria disease development. The KEGG database (https://www.genome.jp/kegg/pathway.html) was used to guide this protein enrichment analysis. Proteins presented in the PPI network (Fig. 3) were categorized according to their involvement in the key malaria disease pathways into four main groups: (i) ubiquitin–proteasome system; (ii) glycerophospholipid metabolism; (iii) DNA replication; and (iv) intracellular anatomical structure (Fig. 3). Altogether, the present protein-protein interaction (PPI) network for the disease malaria provided a brief outline of the interacting proteins and the signaling pathways associated with them, indicating the key proteins that can be considered as critical to the disease development and, thus, as good targets for drug development.

#### Prediction of the target proteins for the isolated compounds

**Fig. 4:**
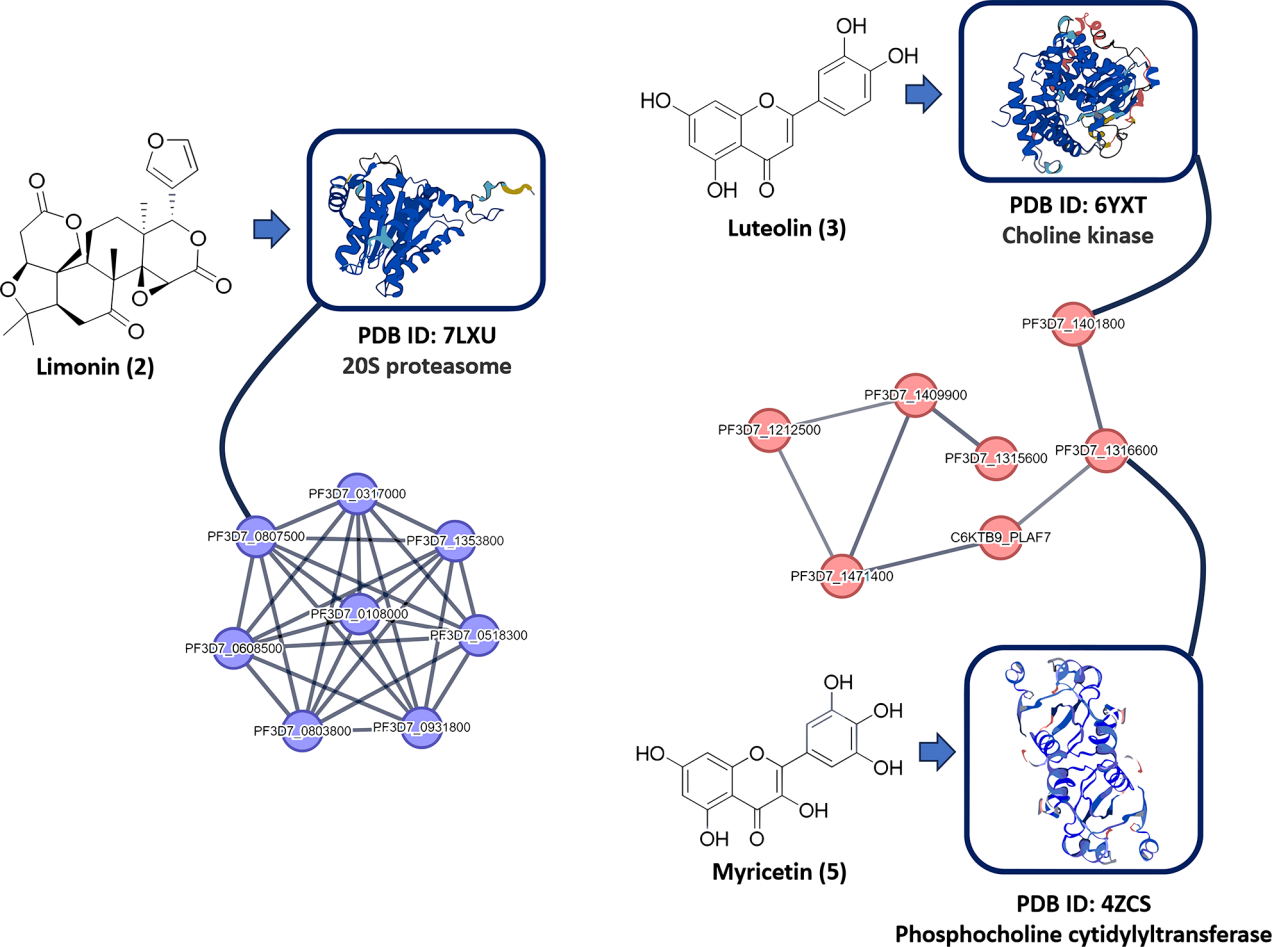
Structures of the *P. falciparum*-derived proteins were predicted to be potential targets for compounds **2** (limonin), **3** (luteolin), and **5** (myricetin). Blue nodes (derived from the PPI network in Fig. 2) represent the proteasome system that limonin (**2**) was predicted to target one of its key proteins (i.e., the 20S proteasome). Red nodes represent the proteins involved in the glycerophospholipid metabolism that luteolin (**3**)  and myricetin (**5**) were predicted to target two of their key proteins (choline kinase and phosphocholine cytidylyltransferase, respectively)

Multiple in silico-based experiments were subsequently performed on compounds **1**–**6** to putatively characterize their potential as possible antiplasmodial agents. The modeled structures of compounds **1**–**6** were run through the PharmMapper (http://www.lilab-ecust.cn/pharmmapper/) prediction platform to see if they might bind to any *P. falciparum*-relevant protein target. PharmMapper is a unique pharmacophore-based virtual screening platform that can match the query structure into the 3D active sites-derived pharmacophore maps of most of the proteins hosted in the Protein Data Bank (PDB; https://www.rcsb.org/) [49]. To select potential targets for compounds **1**–**6**, we set a Fit Score of 6 as a cut-off value. Accordingly, three proteins (i.e., 7LXU, 6YXT, and 4ZCS) were predicted as potential targets for compounds **2**, **3**, and **5**, respectively, of which 7LXU had been identified as hub protein in the *P. falciparum* PPI network described above (Fig. 4). This protein is a key subunit in the 20S proteasome of *P. falciparum*, which has been found recently to be a potential target for new antiplasmodial drugs [50]. The predicted compounds (viz. **2**, **3**, and **5**) were then subjected to molecular docking and MD simulation experiments, which helped us to refine our preliminary pharmacophore-based virtual screening. Proteins were candidate targets if they achieved docking scores below − 7 kcal/mol and absolute binding free energies (Δ*G*_binding_) below − 7 kcal/mol. Docking poses with scores higher than − 7 kcal/mol usually are associated with low affinity in terms of Δ*G*_binding_ [51]. Accordingly, all the predicted proteins followed these conditions and hence, they were subjected to further refinement in 100 ns-long MD simulations.

#### Investigation of the modes of interaction

To investigate the binding modes of limonin (2) inside the active site of one subunit of the 20S proteasome assembly (PDB ID: 7LXU), its modeled structure was redocked into this binding site using AutoDock Vina, and then the top-scoring binding pose was inspected and subjected to a 200 ns-long MD simulation.

**Fig. 5:**
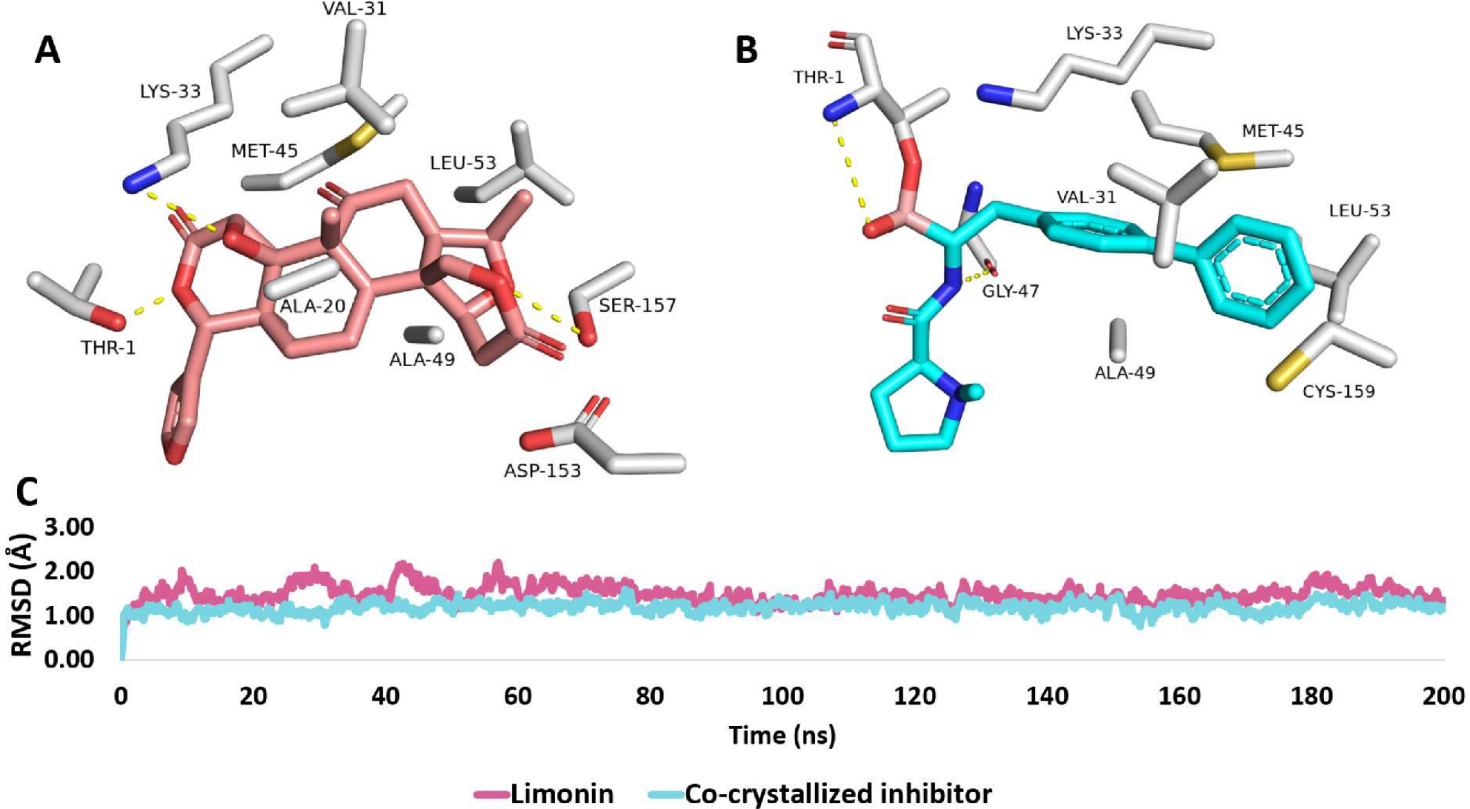
(**A**) Binding modes of limonin (**2**, the brickred-colored structure) alongside (**B**) that of the co-crystallized inhibitor (cyan-colored structures) inside the active site of one subunit of the 20S proteasome assembly (PDB ID: 7LXU) of *P. falciparum* (**A** and **B**, respectively). (**C**) RMSD profiles of limonin (**2**) and the co-crystallized inhibitor over the course of 200 ns-long MD simulation

Limonin (2) and the co-crystallized ligand were found to share a convergent binding mode, where their interactions with the hydrophobic amino acid residues (e.g., VAL-31, LYS-33, MET-45, ALA-49, LEU-53, and CYS-159) were almost the same (Fig. 5A and B). In addition to the covalent interaction with THR-1, the co-crystallized ligand formed a single stable H-bond with GLY-47, while limonin (2) was able to maintain three stable H-bonds with THR-1, LYS-33, and SER-157. Accordingly, the binding stability of both limonin (2) and the co-crystallized ligand, were also convergent, where their RMSD profiles over the course of the MD simulation were almost identical (≈ 1.1 Å), after the first 100 ns (Fig. 5C).

In the case of both luteolin (3) and myricetin (5), each one of them was docked inside the active site of both choline kinase and phosphocholine cytidylyltransferase due to their structural similarity.

**Fig. 6:**
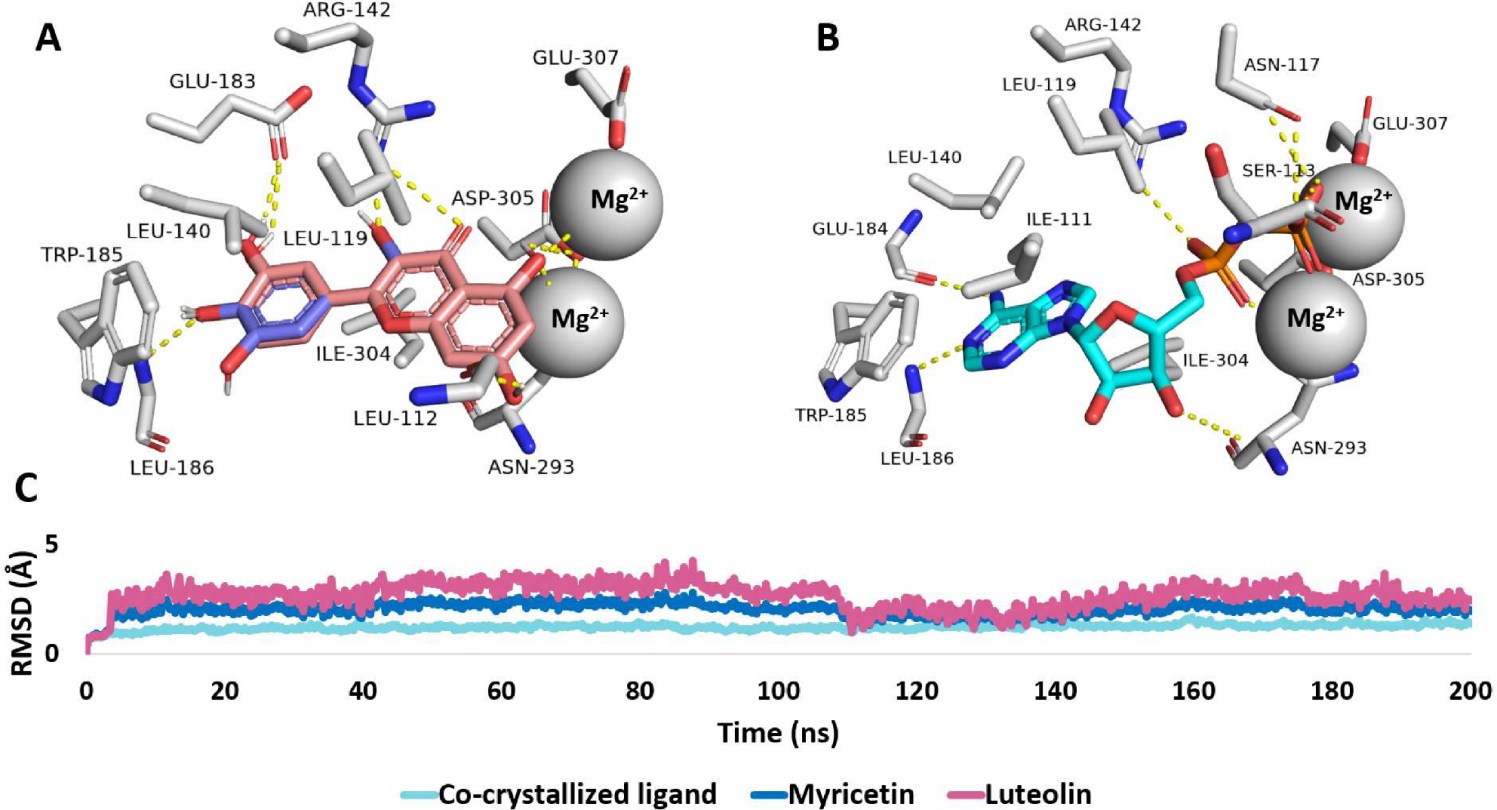
Binding mode of luteolin (**3**, brickred-colored structure) in alignment with myricetin (**5**, blue-colored structures) alongside with that of the co-crystallized ligand (here ADP; cyan-colored structure) inside the choline kinase of *P. falciparum* (**A** and **B**, respectively). RMSD profiles of luteolin (**3**), myricetin (**5**), and the co-crystallized ligand over the course of 200 ns-long MD simulation

In choline kinase, both luteolin (3) and myricetin (5) first shared identical binding modes inside the active site of the enzyme, where they established multiple stable H-bonds with several amino acid residues (Fig. 6). In addition, they formed coordinates interactions with the conserved Mg^2+^. All these hydrophilic interactions were convergent to that of the co-crystallized ligand (ADP). Accordingly, the RMSD profiles of luteolin (3) and myricetin (5) were almost identical, revealing good stability over the course of the MD simulation for both compounds **3** and **5**.

**Fig. 7:**
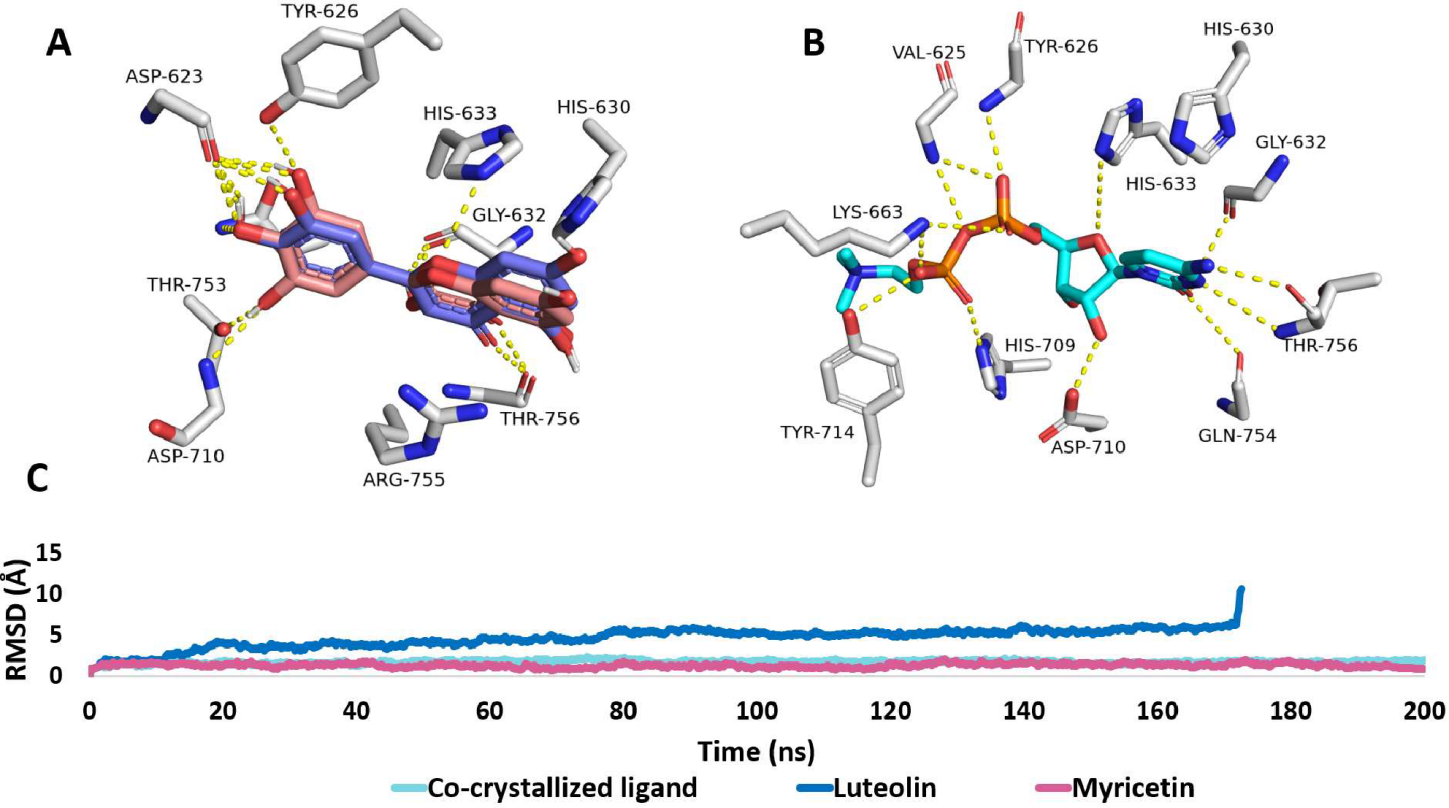
Binding mode of (**A**) luteolin (**3**, blue-colored structure) in alignment with myricetin (**5**, brickred-colored structures) (**B**) alongside with that of the co-crystallized ligand (cyan-colored structure) inside the phosphocholine cytidylyltransferase of *P. falciparum* (**A** and **B**, respectively). (**C**) RMSD profiles of luteolin (3), myricetin (3), and the co-crystallized ligand over the course of 200-ns long MD simulation

Regarding the phosphocholine cytidylyltransferase, both luteolin (3) and myricetin (5) were also identical in their binding poses and almost identical in their interactions, where myricetin (5) was able to form additional stable H-bonds with THR-753 and ASP-710. Hence, this difference was reflected clearly in their RMSD profiles, where luteolin (3) was significantly unstable inside the active site of the enzyme and was detached from it at 173 ns. The RMSD profiles of myricetin (5) and the co-crystallized ligand, by contrast, were stable, showing an average RMSD of 1.7 Å. Accordingly, these important MD simulation-based findings support only myricetin (5) to be a probable inhibitor of the phosphocholine cytidylyltransferase of *P. falciparum* (Fig. 7).

From the previous network pharmacology-based and in silico-based findings, it can be concluded that compounds **2**, **3**, and **5** are promising scaffolds for the development of novel, and efficient antiplasmodial agents that can act via multiple targets and signaling pathways. Accordingly, such a comprehensive bioinformatics-based investigation should be the first step in the development of new therapeutics, particularly from natural products.

## Conclusions

Herein, we investigated the chemical composition of *Citrus aurantifolia* peels by stepwise chromatographic isolation and the subsequent spectroscopic-based structural identification, which offered six known compounds. The isolated compounds were structurally assigned and evaluated in vitro for their antiplasmodial activities against the pathogen responsible for malaria, *Plasmodium falciparum*. The results showed that only the compounds limonin (2), luteolin (3), and myricetin (5) were effective, according to their antiplasmodial activities (IC_50_ 0.2, 3.4, and 5.9 µM, respectively). We explored the antiplasmodial potential of phytochemicals from *C. aurantifolia* peels using a stepwise in silico-based analysis. We first identified the unique proteins of *P. falciparum* that have no homolog in the human proteome, and then performed inverse docking, Δ*G*_Binding_ calculation, and molecular dynamics simulation to predict the binding affinity and stability of the isolated compounds with these proteins. We found that limonin (2), luteolin (3), and myricetin (5) could interact with the 20S proteasome, choline kinase, and phosphocholine cytidylyltransferase, respectively, which are important enzymes for the survival and growth of the parasite. These findings suggest that phytochemicals from *C. aurantifolia* peels may have antiplasmodial activity and could be developed as safe and effective antiplasmodial compounds in the future.

## Materials and methods

### Plant material

Mature fruits of *C. aurantifolia* were collected in January 2021 from the House Garden, Beni-Suef, Egypt, kindly identified by Dr. Abd El-Halim A. Mohammed of the Horticultural Research Institute, Department of Flora, and Phytotaxonomy Research, Dokki, Cairo, Egypt. A voucher specimen (2021-BuPD 119) was deposited at the Department of Pharmacognosy, Faculty of Pharmacy, Beni-Suef University, Egypt.

### Chemicals and reagents

The solvents used in this work included *n*-hexane (*n*-hex., boiling point b.p. 60–80 °C), dichloromethane (DCM), ethyl acetate (EtOAc), ethanol, and methanol (MeOH). They were purchased from El-Nasr Company for Pharmaceuticals and Chemicals (Egypt). Deuterated solvents used for chromatographic and spectroscopic analyses were purchased from Sigma-Aldrich (Saint Louis, Missouri, USA), including dimethyl sulfoxide-*d*_*6*_ (DMSO-*d*_6_), and chloroform-*d* (CDCl_3_-*d*). Column chromatography (CC) was performed using silica gel 60 (63–200 μm, E. Merck, Sigma-Aldrich), while silica gel GF254 for thin-layer chromatography (TLC) (El-Nasr Company for Pharmaceuticals and Chemicals, Egypt) was employed for vacuum liquid chromatography (VLC). Thin-layer chromatography (TLC) was carried out using pre-coated silica gel 60 GF254 plates (E. Merck, Darmstadt, Germany; 20 × 20 cm, 0.25 mm in thickness). Spots were visualized by spraying with *para-*anisaldehyde (PAA) reagent (absolute EtOH:sulfuric acid:glacial acetic acid.:*para*-anisaldehyde (85:5:10:0.5)), followed by heating to 110 °C. luteolin (3), 3ˋ-hydroxygenkwanin (4), myricetin (5), and europetin (6) were purchased from Sigma-Aldrich (Saint Louis, Missouri, USA).

### Spectral analysis

Proton ^1^H and Distortionless Enhancement by Polarization Transfer-Q (DEPT-Q) ^13^C NMR spectra were recorded at 400 and 100 MHz, respectively. Tetramethyl silane (TMS) was used as an internal standard in chloroform-d (CDCl_3_-*d*), and dimethyl sulfoxide-d_6_ (DMSO-d_6_), using the residual solvent peak (δ_H_ = 7.26), and (δ_H_ = 2.50 and δ_C_ = 39.5) as references, respectively. Measurements were performed on a Bruker Advance III 400 MHz with BBFO Smart Probe and a Bruker 400 MHz EON Nitrogen-Free Magnet (Bruker AG, Billerica, MA, USA). Carbon multiplicities were determined using a DEPT-Q experiment, HRESIMS data were obtained using an Acquity Ultra Performance Liquid Chromatography system coupled to a Synapt G2 HDMS quadrupole time-of-flight hybrid mass spectrometer (Waters, Milford, MA, USA). In case of compound 2 the NMR spectra were recorded with a JEOL ECZ 500 (1H:500 MHz, 13C, 125 MHz) at room temperature (T = 25 °C). The ESI mass spectra was measured on an orbitrap mass spectrometer Themro Fisher Exactive driving current : 4 kV, capillary temperature: 300 °C; injection rate: 10 mL/Min. Element analysis data were determined on a HEKATech EUROEA combustionabalyzer.

### Extraction and fractionation of Citrus reticulata peels

*C. aurantifolia* fresh peels (200 g) were finely grind using an OC-60B/60B grinding machine (60–120 mesh, Henan, Mainland China). The ground peels were extracted by maceration using 70% ethanol (300 mL, 3 × for 2 d) at room temperature, and concentrated under vacuum at 45 °C using a rotary evaporator (Buchi Rotavapor R-300, Cole-Parmer, Vernon Hills, IL, USA) to afford 40 g crude extract. The dry extract was suspended in 100 mL of distilled water (H_2_O), and successively portioned with solvents of different polarities (*n*-hex., DCM). The organic phase in each step separately evaporated under reduced pressure to afford the corresponding fractions I (24.0 g) and II (5.0 g), while the remaining mother liquor was concentrated down to give the aqueous fraction (III). All resulting fractions were kept at 4 °C for biological and phytochemical investigations [52–59].

### Isolation and purification of compounds

Fraction II (5 g) was subjected to normal VLC fractionation using silica gel GF_254_ (column 6 × 30 cm, 100 g). Elution was performed using DCM : EtOAc gradient mixtures in the order of increasing polarities (0, 5, 10, 15, 20, 25, 30, 35, 40, 45, 50, 60, 80, and 100%, 250 mL each). The effluents from the column were collected in fractions (250 mL each); and each collected fraction was concentrated and monitored by TLC using the system DCM : EtOAc = 9:1, and the PAA reagent. Similar fractions were grouped and concentrated under reduced pressure to provide two sub-fractions (I_1_ and I_2_). Subfraction I_1_ (1.0 g) was further fractionated on silica gel 60 (100 × 1 cm, 50 g). Elution was performed using a DCM : EtOAc gradient mixtures in the order of increasing polarities (0, 1, 2, 3, 4, 5, 6, 7, 8, 9, and 10%, 1 L each), to afford compound **3**, **4**, **5**, and **6** (7, 10, 13, and 16 mg, respectively). Sub-fraction I_2_ (50 mg) was further fractionated on silica gel 60 (100 × 1 cm, 20 g). Elution was performed using DCM : EtOAc gradient mixtures in the order of increasing polarities (0, 1, 2, 3, 4, 5, 6, 7, 8, 9, and 10%, 1 L each), to afford compounds **1** and **2** (10 and 14 mg, respectively). Compound 2 was further purified on a Sephadex column with MeOH as eluent.

### Antiplasmodial assay

To determine the antiplasmodial effect of isolated compounds in vitro, the Malstat assay was used as described [60]. To synchronize the culture of the *Plasmodium* NF54 strain, parasites with many ring stages were centrifuged, and the pellet was resuspended in the five-fold pellet volume of 5% w/v sorbitol /distilled H_2_O and incubated for 10 min at room temperature. The cells were washed once with RPMI to remove sorbitol and further cultivated. Synchronized ring-stage parasites with 1% parasitemia of the *P. falciparum* NF54 strain were plated in triplicate in 96-well plates (200 µL/well) in the presence of a serial dilution of the extracts dissolved in 0.5% v/v dimethyl sulfoxide (DMSO). The parasites were incubated with the extracts for 72 h at 37 ˚C in the presence of nitrogen containing 5% O_2_ and 5% CO_2_. The incubation of parasites with DMSO at a concentration of 0.5% alone was used as a negative control, and 20% was used as a positive control. An aliquot of 20 µL was removed and added to 100 µL of the Malstat reagent (1% Triton X-100, 10 mg of L-lactate, 3.3 mg Tris and 0.33 mg of APAD (3-acetylpyridine adenine dinucleotide) dissolved in 1 mL of distilled water, pH 9.0) in a new 96-well microtiter plate. The plasmodial lactate dehydrogenase activity was then assessed by adding a 20 µL mixture of NBT (Nitro Blue Tetrazolium)/Diaphorase (1:1; 1 mg/mL stock each) to the Malstat reaction. The optical densities were measured at 630 nm, and the IC_50_ values were calculated from variable-slope sigmoidal dose–response curves using the GraphPad Prism program version 5.

### Screening for the reported *P. falciparum* protein targets

Essential proteins of *P. falciparum* strain 3D7 were retrieved from GeneCards (https://www.genecards.org/) [61], Therapeutic Target Database (TTD, http://db.idrblab.net/ttd/) [62], Comparative Toxicogenomics Database (CTD, http://ctdbase.org/), and DrugBank Database (https://www.drugbank) [63]. The search was narrowed to “*Plasmodium*” and “*falciparum*”. At least two-time repeating targets were chosen.

### Selection of *P. Falciparum* unique proteins

Comparative sequencing analysis using the BLASTp program [64] uncovered the *P. falciparum* critical proteins that lacked a homolog in the human host proteome. In this investigation, we determined that a threshold of 35% query coverage and sequence identity was necessary for meaningful results [65]. We eliminated the proteins that shared a high degree of similarity with the human proteome and focused on the few remaining non-homologs.

### Network construction, molecular docking and MD simulation

Docking was carried out using AutoDock Vina software, and MD simulations were performed with Desmond software, while the construction of PPI network was done using Cytoscape. Detailed descriptions of these procedures can be found in the Supplementary File.

### Electronic supplementary material

Below is the link to the electronic supplementary material.

Supplementary Material 1

## Data Availability

All data generated or analysed during this study are included in this published article, and its supplementary information files.

## Abbreviations

cMD: Classical Molecular Dynamics
DMSO: Dimethyl Sulfoxide
FEP: Free Energy Perturbation
FF14SB: Force Field 14 Stony Brook
Δ*G*_binding_: Absolute Binding Free Energy
GaMD: Gaussian Accelerated Molecular Dynamics
GPUs: Graphics Processing Unit
GUI: Graphical User Interface
IC_50_: Half-maximal Inhibitory Concentration
*K*_i_: Inhibitor Constant
MDS: Molecular Dynamics Simulation
MOI: Multiplicity of Infection
M^pro^: Main Protease
NAMD: Nanoscale Molecular Dynamics
NIQ: Naphthylisoquinoline
NPT: Constant Pressure and Temperature
OPLS: Optimized Potentials for Liquid Simulations
PCR: Polymerase Chain Reaction
PDB: Protein Data Bank
PME: Particle Mesh Ewald
pN: Piconewton
RMSD: Root-Mean-Square Deviation
RMSF: Root-Mean-Square Fluctuation
RT: Reverse Transcriptase
RTqPCR: Quantitative Reverse Transcription Polymerase Chain Reaction
PPI: protein-protein interaction
SHAKE: Secure Hash Algorithm Keccak
SMD: Steered Molecular Dynamics
TIP3P: Transferable Intermolecular Potential with 3 Points
VMD: Visual Molecular Dynamics
