# Supplementary material for: Antiplasmodial potential of phytochemicals from Citrus aurantifolia peels: a comprehensive in vitro and in silico study
Source: BMC Chem. 2024 Mar 30;18(1):60. doi: 10.1186/s13065-024-01162-x (PMC10981828; doi:10.1186/s13065-024-01162-x)
Supplement: Supplementary file 1 — Supplementary Material 1 [file 13065_2024_1162_MOESM1_ESM.docx]

**Antiplasmodial Potential of Phytochemicals from *Citrus aurantifolia* Peels: A Comprehensive *in vitro* and *in silico* Study**

**Abeer H. Elmaidomy^1*^, Usama Ramadan Abdelmohsen^2,3*^, Ahmed M. Sayed^4^, Faisal H. Altemani^5^, Naseh A. Algehainy^5^, Denisa Soost^6^, Thomas Paululat^6^, Gerhard Bringmann^7*^, and Esraa M. Mohamed^8^**

^1^ Department of Pharmacognosy, Faculty of Pharmacy, Beni-Suef University, Beni-Suef 62514, Egypt

^2^ Department of Pharmacognosy, Faculty of Pharmacy, Minia University, Minia 61519, Egypt

^3^ Department of Pharmacognosy, Faculty of Pharmacy, Deraya University, Minia 61111, Egypt

^4^ Department of Pharmacognosy, Faculty of Pharmacy, Nahda University, 62513 Beni-Suef, Egypt

^5^ Department of Medical Laboratory Technology, Faculty of Applied Medical Sciences, University of Tabuk, Tabuk 71491, Saudi Arabia.

^6^ Department of Chemistry and Biology, University of Siegen, Adolf-Reichwein-Str. 2, 57068, Siegen, Germany

^7^ Institute of Organic Chemistry, University of Würzburg, Am Hubland, 97074, Würzburg, Germany

^8^ Department of Pharmacognosy, Faculty of Pharmacy, MUST, Giza, 12566, Egypt

***** Correspondence: authors: usama.ramadan@mu.edu.eg (URA), bringman@chemie.uni-wuerzburg.de (GB), and Abeer011150@pharm.bsu.edu.eg (AHE).

**Methods**

***1. Virtual Target Identification***

The putative target characterization was achieved by pharmacophore-based virtual screening using PharmMapper [1]. This platform assigns a score to each molecule in the PDB that best fits a pharmacophore model that has been extracted and stored as a library ligand dataset in mol2 format. By this procedure, an almost 77 score matrix is produced. After that, when a new molecule is submitted, its fit score is calculated for each pharmacophore, and then each fit score for that pharmacophore is compared to the fit score matrix to determine where it falls on the scale of all the pharmacophore scores. In comparison to chance pharmacophore matching, the pure fit score that results from this procedure carries considerably more weight and assurance. The query structure was submitted to the platform in the PDB format, and the retrieved results were exported as Excel sheet arranging the resulted protein targets according to their fit scores.

***2. Docking Study***

The crystal structures of the proteins used for docking were retrieved from PDB. The AutoDock Vina docking machine was used for the docking experiments [2]. The co-crystallized ligand in each protein was used to determine the binding site and the docking grid-box.

The ligand to binding site shape matching root means square deviation (RMSD) threshold was set to 2.0 Å. The interaction energies were determined using the Charmm force field (v.1.02) with 10.0 Å as a non-bonded cutoff distance and distance-dependent dielectric. Then, 5.0 Å was set as an energy grid extending from the binding site [3]. The tested compounds were energy-minimized inside the selected binding pocket. The editing and visualization of the generated binding poses were performed using PyMOL software [4].

***3. Molecular Dynamics Simulation***

NAMD 3.0.0. software was used for performing MDS [3,5]. This software applies the Charmm-36 force field. Protein systems were built using the QwikMD toolkit of the VMD 1.9.4. software [5,6], where the protein structure was checked for any missing hydrogens, the protonation states of the amino acid residues were set (pH = 7.4), and the co-crystallized water molecules were removed. Metal-containing proteins like choline kinase that contain Mg^+2^ ions in the active site should be parameterized during the protein preparation step. To do so, a hetero state should be generated for hetero atoms like Mg (Generate Hetero States). This function is a part of the VMD 1.9.4.'s Protein Preparation wizard. Harmonic Tcl forces were applied to keep Mg^+2^ in place. Thereafter, the whole structure was embedded in an orthorhombic box of TIP3P water together with 0.15 M Na^+^ and Cl^-^ ions in 20 Å solvent buffer. The prepared systems were then energy-minimized and equilibrated for 5 ns. The parameters and topologies of the ligands were calculated by using the VMD 1.9.4. plugin Force Field Toolkit (ffTK). Subsequently, the generated parameters and topology files were loaded to VMD to readily read the protein–ligand complexes without errors and then conduct the simulation steps.

***4. Binding Free Energy Calculations***

Molecular Mechanics Poisson-Boltzmann Surface Area (MM-PBSA) embedded in the MMPBSA.py module of AMBER18 was utilized to calculate the binding free energy of the docked complex [7]. A total of 100 frames were processed from the trajectories, and the net energy of the system was estimated using the following equation:

Δ*G*_Binding_ = Δ*G*_Complex_ – Δ*G*_Receptor_ – Δ*G_I_*_nhibitor_

Each of the aforementioned terms requires the calculation of multiple energy components, including van der Waals energy, electrostatic energy, internal energy from molecular mechanics, and polar contribution to solvation energy.

***5. Networks Construction***

All retrieved *P. falciparum*-relevant proteins were submitted to STRING database [8] in order to find out the potential interactions between them. Cytoscape 3.8.2 (<https://www.cytoscape.org/>) was used for the PPI networks constructions and analysis [9] using the interaction data exported from the STRIN analysis. The lowest interaction score was set to 0.4, and the rest of the parameters were set to the default setting to obtain the PPI networks.

**Table S1.** *P. falciparum* proteins

| **Protein ID** | **Protein name** |
| --- | --- |
| C6KST8\|C6KST8 | Zinc transporter ZIP1 |
| Q9U0K3\|Q9U0K3 | Zinc finger Ran-binding domain-containing protein 2 |
| Q9U0I8\|Q9U0I8 | Zinc finger protein |
| C0H4E5\|C0H4E5 | Zinc finger protein |
| Q8I3R4\|Q8I3R4 | Zinc finger protein |
| Q8IB20\|Q8IB20 | Zinc finger protein |
| Q8I331\|Q8I331 | Zinc finger protein |
| C0H566\|C0H566 | Zinc finger protein |
| Q8I5L2\|Q8I5L2 | Zinc finger protein |
| Q8I4Z8\|Q8I4Z8 | Zinc finger protein |
| Q8IEL2\|Q8IEL2 | Zinc finger protein |
| A0A5K1K928\|A0A5K1K928 | Zinc finger protein |
| Q8IDC0\|Q8IDC0 | Zinc finger protein |
| Q8IM65\|Q8IM65 | Zinc finger protein |
| Q8ILV4\|Q8ILV4 | Zinc finger protein |
| Q8ILT7\|Q8ILT7 | Zinc finger protein |
| Q8ILC9\|Q8ILC9 | Zinc finger protein |
| Q8IL66\|Q8IL66 | Zinc finger protein |
| Q8IKP5\|Q8IKP5 | Zinc finger protein |
| Q8IKJ5\|Q8IKJ5 | Zinc finger protein |
| Q8IKJ3\|Q8IKJ3 | Zinc finger protein |
| O77322\|O77322 | Zinc finger protein |
| O97273\|O97273 | Zinc finger protein |
| A0A5K1K8I4\|A0A5K1K8I4 | Zinc finger protein |
| Q8I3L5\|Q8I3L5 | Zinc finger protein |
| Q8IJV8\|Q8IJV8 | Zinc finger (CCCH type) protein |
| A0A5K1K8E3\|A0A5K1K8E3 | Zinc finger (CCCH type) protein |
| O77335\|O77335 | YTH domain-containing protein |
| Q8IL91\|Q8IL91 | YrhK domain-containing protein OS |
| C6S3J4\|C6S3J4 | Yos1-like protein |
| Q8IDB8\|Q8IDB8 | YOP1-like protein |
| Q8IKJ7\|Q8IKJ7 | YL1 nuclear protein |
| C0H470\|C0H470 | YGGT family protein |
| C0H4S5\|C0H4S5 | YEATS domain-containing protein |
| Q8IIA5\|Q8IIA5 | XTBD domain-containing protein |
| Q8I3G2\|Q8I3G2 | XAP-5 DNA binding protein |
| Q8IIQ0\|Q8IIQ0 | WW domain-containing protein OS |
| Q8IL37\|Q8IL37 | WD repeat-containing protein OS |
| Q8IDM8\|Q8IDM8 | WD repeat-containing protein 92 |
| Q8I4Z2\|Q8I4Z2 | WD repeat-containing protein 82 |
| Q8I3K2\|Q8I3K2 | WD repeat-containing protein 55 |
| O97292\|O97292 | WD repeat-containing protein |
| Q8IJZ5\|Q8IJZ5 | WD repeat-containing protein |
| Q8IJB3\|Q8IJB3 | WD repeat-containing protein |
| Q8II99\|Q8II99 | WD repeat-containing protein |
| Q8IHX6\|Q8IHX6 | WD repeat-containing protein |
| Q8I5H3\|Q8I5H3 | WD repeat-containing protein |
| Q8I5B5\|Q8I5B5 | WD repeat-containing protein |
| Q8I5A2\|Q8I5A2 | WD repeat-containing protein |
| A0A5K1K8S8\|A0A5K1K8S8 | WD repeat-containing protein |
| A0A144A0U7\|A0A144A0U7 | W2 domain-containing protein OS |
| Q8IKJ0\|Q8IKJ0 | V-type proton ATPase subunit OS |
| Q8IEP9\|Q8IEP9 | V-type proton ATPase subunit H OS |
| Q8IE84\|Q8IE84 | V-type proton ATPase subunit G |
| Q8IHW4\|Q8IHW4 | V-type proton ATPase subunit F OS |
| Q8I2H3\|Q8I2H3 | V-type proton ATPase subunit E |
| Q8IDS0\|Q8IDS0 | V-type proton ATPase subunit D |
| Q8I280\|Q8I280 | V-type proton ATPase subunit C OS |
| Q8IAQ8\|Q8IAQ8 | V-type proton ATPase subunit a |
| Q8I3R7\|Q8I3R7 | V-type proton ATPase proteolipid subunit OS |
| Q8IDF7\|Q8IDF7 | V-type proton ATPase 21 kDa proteolipid subunit |
| C0H4P0\|C0H4P0 | V-type ATPase V0 subunit e |
| Q8IHX3\|Q8IHX3 | VPS9 domain-containing protein |
| Q8IAL0\|Q8IAL0 | von Willebrand factor A domain-related protein OS |
| Q8ILE3\|Q8ILE3 | Voltage-dependent anion-selective channel protein |
| C6KSR6\|C6KSR6 | VFT protein OS |
| O77313\|O77313 | Vesicle-fusing ATPase OS |
| Q8IL71\|Q8IL71 | Vesicle-associated membrane protein |
| Q8I563\|Q8I563 | Vesicle transport v-SNARE protein VTI1 |
| A0A143ZX23\|A0A143ZX23 | Vesicle transport v-SNARE protein |
| Q8IE98\|Q8IE98 | Vesicle transport protein OS |
| C0H5F1\|C0H5F1 | Very-long-chain (3R)-3-hydroxyacyl-CoA dehydratase OS |
| Q8IKL5\|Q8IKL5 | Valyl-tRNA synthetase OS |
| O77354\|O77354 | Valine-tRNA ligase |
| Q8IK92\|Q8IK92 | Vacuole membrane protein 1 |
| A0A144A0N0\|A0A144A0N0 | Vacuolar-sorting protein SNF |
| C6KT60\|C6KT60 | Vacuolar transporter chaperone |
| Q6ZMA8\|Q6ZMA8 | Vacuolar proton pump subunit B OS |
| B9ZSH9\|B9ZSH9 | Vacuolar protein sorting-associated protein VTA1 |
| Q8IAZ5\|Q8IAZ5 | Vacuolar protein sorting-associated protein 9 |
| Q8IBI7\|Q8IBI7 | Vacuolar protein sorting-associated protein 53 |
| A0A5K1K8G8\|A0A5K1K8G8 | Vacuolar protein sorting-associated protein 52 |
| Q8I2A9\|Q8I2A9 | Vacuolar protein sorting-associated protein 51 |
| Q8I388\|Q8I388 | Vacuolar protein sorting-associated protein 46 |
| O96243\|O96243 | Vacuolar protein sorting-associated protein 45 OS |
| Q8IKQ5\|Q8IKQ5 | Vacuolar protein sorting-associated protein 4 OS |
| Q8IIQ6\|Q8IIQ6 | Vacuolar protein sorting-associated protein 35 OS |
| Q8I2G8\|Q8I2G8 | Vacuolar protein sorting-associated protein 33 |
| Q8IM27\|Q8IM27 | Vacuolar protein sorting-associated protein 29 OS |
| A0A144A4V4\|A0A144A4V4 | Vacuolar protein sorting-associated protein 26 |
| Q8IAZ9\|Q8IAZ9 | Vacuolar protein sorting-associated protein 2 |
| Q8IEL8\|Q8IEL8 | Vacuolar protein sorting-associated protein 18 |
| Q8I524\|Q8I524 | Vacuolar protein sorting-associated protein 16 |
| Q8I5I0\|Q8I5I0 | Vacuolar iron transporter OS |
| Q8IDH2\|Q8IDH2 | Vacuolar fusion protein MON1 |
| Q8I5G0\|Q8I5G0 | VAC14 domain-containing protein |
| A0A144A0S1\|A0A144A0S1 | Uroporphyrinogen-III synthase |
| Q8ILU6\|Q8ILU6 | Uracil-DNA glycosylase |
| B9ZSI0\|B9ZSI0 | Uncharacterized protein |
| Q8I299\|Q8I299 | Uncharacterized protein |
| B9ZSI2\|B9ZSI2 | Uncharacterized protein |
| Q8I290\|Q8I290 | Uncharacterized protein |
| Q8I287\|Q8I287 | Uncharacterized protein |
| Q8I286\|Q8I286 | Uncharacterized protein |
| B9ZSI3\|B9ZSI3 | Uncharacterized protein |
| Q8I277\|Q8I277 | Uncharacterized protein |
| Q8I276\|Q8I276 | Uncharacterized protein |
| Q8I271\|Q8I271 | Uncharacterized protein |
| Q8I262\|Q8I262 | Uncharacterized protein |
| Q8I260\|Q8I260 | Uncharacterized protein |
| Q8I256\|Q8I256 | Uncharacterized protein |
| Q8I234\|Q8I234 | Uncharacterized protein |
| B9ZSI9\|B9ZSI9 | Uncharacterized protein |
| Q8I230\|Q8I230 | Uncharacterized protein |
| Q8I229\|Q8I229 | Uncharacterized protein |
| Q8I227\|Q8I227 | Uncharacterized protein |
| Q9U0M7\|Q9U0M7 | Uncharacterized protein |
| Q8I2D5\|Q8I2D5 | Uncharacterized protein |
| Q8I2D3\|Q8I2D3 | Uncharacterized protein |
| Q8I670\|Q8I670 | Uncharacterized protein |
| O96119\|O96119 | Uncharacterized protein |
| O96126\|O96126 | Uncharacterized protein |
| C6S3A7\|C6S3A7 | Uncharacterized protein |
| C6S3A8\|C6S3A8 | Uncharacterized protein |
| Q8I665\|Q8I665 | Uncharacterized protein |
| O96151\|O96151 | Uncharacterized protein |
| O96170\|O96170 | Uncharacterized protein |
| O96174\|O96174 | Uncharacterized protein |
| O96187\|O96187 | Uncharacterized protein |
| O96188\|O96188 | Uncharacterized protein |
| O96190\|O96190 | Uncharacterized protein |
| O96201\|O96201 | Uncharacterized protein |
| O96205\|O96205 | Uncharacterized protein |
| O96211\|O96211 | Uncharacterized protein |
| O96213\|O96213 | Uncharacterized protein |
| C6S3B6\|C6S3B6 | Uncharacterized protein |
| O96217\|O96217 | Uncharacterized protein |
| O96224\|O96224 | Uncharacterized protein |
| O96225\|O96225 | Uncharacterized protein |
| O96227\|O96227 | Uncharacterized protein |
| O96228\|O96228 | Uncharacterized protein |
| O96235\|O96235 | Uncharacterized protein |
| C6S3B7\|C6S3B7 | Uncharacterized protein |
| O96240\|O96240 | Uncharacterized protein |
| A0A143ZWT2\|A0A143ZWT2 | Uncharacterized protein |
| O96253\|O96253 | Uncharacterized protein |
| A0A143ZYK0\|A0A143ZYK0 | Uncharacterized protein |
| O96256\|O96256 | Uncharacterized protein |
| O96259\|O96259 | Uncharacterized protein |
| O96261\|O96261 | Uncharacterized protein |
| Q8I656\|Q8I656 | Uncharacterized protein |
| O96274\|O96274 | Uncharacterized protein |
| Q7KWI7\|Q7KWI7 | Uncharacterized protein |
| Q7KWI6\|Q7KWI6 | Uncharacterized protein |
| O96278\|O96278 | Uncharacterized protein |
| Q8I653\|Q8I653 | Uncharacterized protein |
| Q8I651\|Q8I651 | Uncharacterized protein |
| O97335\|O97335 | Uncharacterized protein |
| C0H467\|C0H467 | Uncharacterized protein |
| O97331\|O97331 | Uncharacterized protein |
| O97336\|O97336 | Uncharacterized protein |
| O97332\|O97332 | Uncharacterized protein |
| O97333\|O97333 | Uncharacterized protein |
| O97234\|O97234 | Uncharacterized protein |
| Q9NLB6\|Q9NLB6 | Uncharacterized protein |
| Q9NLB5\|Q9NLB5 | Uncharacterized protein |
| Q9NLB4\|Q9NLB4 | Uncharacterized protein |
| Q9NLB3\|Q9NLB3 | Uncharacterized protein |
| O97243\|O97243 | Uncharacterized protein |
| O97255\|O97255 | Uncharacterized protein |
| O77324\|O77324 | Uncharacterized protein |
| O77326\|O77326 | Uncharacterized protein |
| Q9NLA9\|Q9NLA9 | Uncharacterized protein |
| Q9NLA8\|Q9NLA8 | Uncharacterized protein |
| O77359\|O77359 | Uncharacterized protein |
| O77357\|O77357 | Uncharacterized protein |
| O77356\|O77356 | Uncharacterized protein |
| Q9NFE4\|Q9NFE4 | Uncharacterized protein |
| O77342\|O77342 | Uncharacterized protein |
| O77340\|O77340 | Uncharacterized protein |
| O77339\|O77339 | Uncharacterized protein |
| O77337\|O77337 | Uncharacterized protein |
| C0H471\|C0H471 | Uncharacterized protein |
| C0H472\|C0H472 | Uncharacterized protein |
| A0A143ZV68\|A0A143ZV68 | Uncharacterized protein |
| C0H475\|C0H475 | Uncharacterized protein |
| O97269\|O97269 | Uncharacterized protein |
| O97274\|O97274 | Uncharacterized protein |
| C0H479\|C0H479 | Uncharacterized protein |
| O77369\|O77369 | Uncharacterized protein |
| O97279\|O97279 | Uncharacterized protein |
| O97286\|O97286 | Uncharacterized protein |
| O97291\|O97291 | Uncharacterized protein |
| O97298\|O97298 | Uncharacterized protein |
| O97299\|O97299 | Uncharacterized protein |
| O97300\|O97300 | Uncharacterized protein |
| O97301\|O97301 | Uncharacterized protein |
| Q8I1Z7\|Q8I1Z7 | Uncharacterized protein |
| Q8I1Y9\|Q8I1Y9 | Uncharacterized protein |
| Q8I1Y7\|Q8I1Y7 | Uncharacterized protein |
| Q8I1X0\|Q8I1X0 | Uncharacterized protein |
| Q8I1W8\|Q8I1W8 | Uncharacterized protein |
| Q9U0M0\|Q9U0M0 | Uncharacterized protein |
| Q9U0L6\|Q9U0L6 | Uncharacterized protein |
| Q9U0K6\|Q9U0K6 | Uncharacterized protein |
| Q9U0K4\|Q9U0K4 | Uncharacterized protein |
| C0H499\|C0H499 | Uncharacterized protein |
| Q9U0J8\|Q9U0J8 | Uncharacterized protein |
| Q9U0I3\|Q9U0I3 | Uncharacterized protein |
| Q9U0I2\|Q9U0I2 | Uncharacterized protein |
| Q9U0I0\|Q9U0I0 | Uncharacterized protein |
| Q9U0H5\|Q9U0H5 | Uncharacterized protein |
| Q9U0G8\|Q9U0G8 | Uncharacterized protein |
| Q8I1T0\|Q8I1T0 | Uncharacterized protein |
| Q8I1S5\|Q8I1S5 | Uncharacterized protein |
| C0H4A9\|C0H4A9 | Uncharacterized protein |
| Q8I1R8\|Q8I1R8 | Uncharacterized protein |
| C0H4B0\|C0H4B0 | Uncharacterized protein |
| C0H4B2\|C0H4B2 | Uncharacterized protein |
| C0H4B5\|C0H4B5 | Uncharacterized protein |
| Q8I1P7\|Q8I1P7 | Uncharacterized protein |
| Q8I1P6\|Q8I1P6 | Uncharacterized protein |
| A0A146LZZ4\|A0A146LZZ4 | Uncharacterized protein |
| Q8IFP8\|Q8IFP8 | Uncharacterized protein |
| Q8IFN3\|Q8IFN3 | Uncharacterized protein |
| Q8IFM7\|Q8IFM7 | Uncharacterized protein |
| Q8I477\|Q8I477 | Uncharacterized protein |
| Q8I475\|Q8I475 | Uncharacterized protein |
| Q8I448\|Q8I448 | Uncharacterized protein |
| Q8I442\|Q8I442 | Uncharacterized protein |
| Q8I439\|Q8I439 | Uncharacterized protein |
| A0A5K1K846\|A0A5K1K846 | Uncharacterized protein |
| Q8I432\|Q8I432 | Uncharacterized protein |
| C0H4D1\|C0H4D1 | Uncharacterized protein |
| A0A5K1K9E3\|A0A5K1K9E3 | Uncharacterized protein |
| Q8I407\|Q8I407 | Uncharacterized protein |
| Q8I403\|Q8I403 | Uncharacterized protein |
| Q8I3Y7\|Q8I3Y7 | Uncharacterized protein |
| Q8I3Y5\|Q8I3Y5 | Uncharacterized protein |
| Q8I3Y2\|Q8I3Y2 | Uncharacterized protein |
| Q8I3Y1\|Q8I3Y1 | Uncharacterized protein |
| Q8I3X9\|Q8I3X9 | Uncharacterized protein |
| Q8I3X7\|Q8I3X7 | Uncharacterized protein |
| C0H4D5\|C0H4D5 | Uncharacterized protein |
| Q8I3W8\|Q8I3W8 | Uncharacterized protein |
| Q8I3V9\|Q8I3V9 | Uncharacterized protein |
| C0H4E2\|C0H4E2 | Uncharacterized protein |
| Q8I3S0\|Q8I3S0 | Uncharacterized protein |
| Q8I3R1\|Q8I3R1 | Uncharacterized protein |
| A0A5K1K8L5\|A0A5K1K8L5 | Uncharacterized protein |
| C0H4F4\|C0H4F4 | Uncharacterized protein |
| Q8I3P8\|Q8I3P8 | Uncharacterized protein |
| Q8I3P3\|Q8I3P3 | Uncharacterized protein |
| Q8I3P2\|Q8I3P2 | Uncharacterized protein |
| A0A5K1K8Q3\|A0A5K1K8Q3 | Uncharacterized protein |
| Q8I3M8\|Q8I3M8 | Uncharacterized protein |
| Q8I3M4\|Q8I3M4 | Uncharacterized protein |
| Q8I3M3\|Q8I3M3 | Uncharacterized protein |
| Q8I3M2\|Q8I3M2 | Uncharacterized protein |
| Q8I3M0\|Q8I3M0 | Uncharacterized protein |
| Q8I3K8\|Q8I3K8 | Uncharacterized protein |
| Q8I3K4\|Q8I3K4 | Uncharacterized protein |
| Q8I3K1\|Q8I3K1 | Uncharacterized protein |
| Q8I3I4\|Q8I3I4 | Uncharacterized protein |
| C0H4G5\|C0H4G5 | Uncharacterized protein |
| C0H4G6\|C0H4G6 | Uncharacterized protein |
| A0A143ZXE1\|A0A143ZXE1 | Uncharacterized protein |
| Q8I3G7\|Q8I3G7 | Uncharacterized protein |
| Q8I3G4\|Q8I3G4 | Uncharacterized protein |
| Q8I3F5\|Q8I3F5 | Uncharacterized protein |
| Q8I3E9\|Q8I3E9 | Uncharacterized protein |
| C0H4H1\|C0H4H1 | Uncharacterized protein |
| C6KSL9\|C6KSL9 | Uncharacterized protein |
| C6KSN8\|C6KSN8 | Uncharacterized protein |
| C6KSP2\|C6KSP2 | Uncharacterized protein |
| C6KSP7\|C6KSP7 | Uncharacterized protein |
| C6KSR2\|C6KSR2 | Uncharacterized protein |
| C6KSS7\|C6KSS7 | Uncharacterized protein |
| C6KSS9\|C6KSS9 | Uncharacterized protein |
| C6KST0\|C6KST0 | Uncharacterized protein |
| C6KST2\|C6KST2 | Uncharacterized protein |
| C6KST4\|C6KST4 | Uncharacterized protein |
| C6KST6\|C6KST6 | Uncharacterized protein |
| C6KSU0\|C6KSU0 | Uncharacterized protein |
| C6KSU6\|C6KSU6 | Uncharacterized protein |
| C6KSV2\|C6KSV2 | Uncharacterized protein |
| C6KSW4\|C6KSW4 | Uncharacterized protein |
| C6KSW8\|C6KSW8 | Uncharacterized protein |
| C6KSX3\|C6KSX3 | Uncharacterized protein |
| C6KSX4\|C6KSX4 | Uncharacterized protein |
| C6KSX8\|C6KSX8 | Uncharacterized protein |
| C6KSZ1\|C6KSZ1 | Uncharacterized protein |
| C6KT04\|C6KT04 | Uncharacterized protein |
| C6KT05\|C6KT05 | Uncharacterized protein |
| C6KT07\|C6KT07 | Uncharacterized protein |
| C6KT14\|C6KT14 | Uncharacterized protein |
| C6KT21\|C6KT21 | Uncharacterized protein |
| C6KT22\|C6KT22 | Uncharacterized protein |
| C6KT31\|C6KT31 | Uncharacterized protein |
| C6KT32\|C6KT32 | Uncharacterized protein |
| C6KT36\|C6KT36 | Uncharacterized protein |
| C6KT38\|C6KT38 | Uncharacterized protein |
| C6KT42\|C6KT42 | Uncharacterized protein |
| C6KT48\|C6KT48 | Uncharacterized protein |
| C6KT49\|C6KT49 | Uncharacterized protein |
| C6KT57\|C6KT57 | Uncharacterized protein |
| C6KT61\|C6KT61 | Uncharacterized protein |
| C6KT77\|C6KT77 | Uncharacterized protein |
| C6KT80\|C6KT80 | Uncharacterized protein |
| C6KT88\|C6KT88 | Uncharacterized protein |
| C6KT90\|C6KT90 | Uncharacterized protein |
| C6KT91\|C6KT91 | Uncharacterized protein |
| C6KT93\|C6KT93 | Uncharacterized protein |
| C6KT98\|C6KT98 | Uncharacterized protein |
| C6KTA2\|C6KTA2 | Uncharacterized protein |
| C6KTA6\|C6KTA6 | Uncharacterized protein |
| C6KTB2\|C6KTB2 | Uncharacterized protein |
| C6KTC2\|C6KTC2 | Uncharacterized protein |
| C0H4K0\|C0H4K0 | Uncharacterized protein |
| Q8IC40\|Q8IC40 | Uncharacterized protein |
| Q8IC38\|Q8IC38 | Uncharacterized protein |
| Q8IC33\|Q8IC33 | Uncharacterized protein |
| A0A143ZY62\|A0A143ZY62 | Uncharacterized protein |
| Q8IC18\|Q8IC18 | Uncharacterized protein |
| C0H4L4\|C0H4L4 | Uncharacterized protein |
| C0H4L7\|C0H4L7 | Uncharacterized protein |
| C0H4L9\|C0H4L9 | Uncharacterized protein |
| Q8IC14\|Q8IC14 | Uncharacterized protein |
| Q8IBZ4\|Q8IBZ4 | Uncharacterized protein |
| C0H4M4\|C0H4M4 | Uncharacterized protein |
| Q8IBZ0\|Q8IBZ0 | Uncharacterized protein |
| Q8IBY9\|Q8IBY9 | Uncharacterized protein |
| Q8IBY8\|Q8IBY8 | Uncharacterized protein |
| C0H4M5\|C0H4M5 | Uncharacterized protein |
| Q8IBY5\|Q8IBY5 | Uncharacterized protein |
| Q8IBY3\|Q8IBY3 | Uncharacterized protein |
| Q8IBX9\|Q8IBX9 | Uncharacterized protein |
| Q8IBX8\|Q8IBX8 | Uncharacterized protein |
| Q8IBX7\|Q8IBX7 | Uncharacterized protein |
| C0H4M7\|C0H4M7 | Uncharacterized protein |
| Q8IBV1\|Q8IBV1 | Uncharacterized protein |
| Q8IBU3\|Q8IBU3 | Uncharacterized protein |
| Q8IBT7\|Q8IBT7 | Uncharacterized protein |
| Q8IBT3\|Q8IBT3 | Uncharacterized protein |
| C0H4N2\|C0H4N2 | Uncharacterized protein |
| A0A143ZVJ7\|A0A143ZVJ7 | Uncharacterized protein |
| C0H4N6\|C0H4N6 | Uncharacterized protein |
| C0H4N7\|C0H4N7 | Uncharacterized protein |
| Q8IBR1\|Q8IBR1 | Uncharacterized protein |
| Q8IBQ4\|Q8IBQ4 | Uncharacterized protein |
| Q8IBQ3\|Q8IBQ3 | Uncharacterized protein |
| Q8IBQ0\|Q8IBQ0 | Uncharacterized protein |
| Q8IBP9\|Q8IBP9 | Uncharacterized protein |
| C0H4N9\|C0H4N9 | Uncharacterized protein |
| Q8IBP6\|Q8IBP6 | Uncharacterized protein |
| A0A143ZWQ6\|A0A143ZWQ6 | Uncharacterized protein |
| Q8IBP0\|Q8IBP0 | Uncharacterized protein |
| Q8IBN9\|Q8IBN9 | Uncharacterized protein |
| Q8IBN6\|Q8IBN6 | Uncharacterized protein |
| Q8IBN3\|Q8IBN3 | Uncharacterized protein |
| Q8IBN2\|Q8IBN2 | Uncharacterized protein |
| Q8IBM6\|Q8IBM6 | Uncharacterized protein |
| Q8IBM4\|Q8IBM4 | Uncharacterized protein |
| Q8IBM1\|Q8IBM1 | Uncharacterized protein |
| Q8IBM0\|Q8IBM0 | Uncharacterized protein |
| Q8IBL5\|Q8IBL5 | Uncharacterized protein |
| Q8IBL1\|Q8IBL1 | Uncharacterized protein |
| Q8IBK7\|Q8IBK7 | Uncharacterized protein |
| C0H4P2\|C0H4P2 | Uncharacterized protein |
| Q8IBK0\|Q8IBK0 | Uncharacterized protein |
| Q8IBJ8\|Q8IBJ8 | Uncharacterized protein |
| Q8IBJ7\|Q8IBJ7 | Uncharacterized protein |
| C0H4P4\|C0H4P4 | Uncharacterized protein |
| Q8IBJ0\|Q8IBJ0 | Uncharacterized protein |
| Q8IBI9\|Q8IBI9 | Uncharacterized protein |
| C0H4P6\|C0H4P6 | Uncharacterized protein |
| A0A146M1S3\|A0A146M1S3 | Uncharacterized protein |
| Q8IBI0\|Q8IBI0 | Uncharacterized protein |
| Q8IBH4\|Q8IBH4 | Uncharacterized protein |
| Q8IBH3\|Q8IBH3 | Uncharacterized protein |
| Q8IBH0\|Q8IBH0 | Uncharacterized protein |
| Q8IBG3\|Q8IBG3 | Uncharacterized protein |
| Q8IBG0\|Q8IBG0 | Uncharacterized protein |
| Q8IAK7\|Q8IAK7 | Uncharacterized protein |
| A0A5K1K9E5\|A0A5K1K9E5 | Uncharacterized protein |
| Q8IAL4\|Q8IAL4 | Uncharacterized protein |
| C0H4R2\|C0H4R2 | Uncharacterized protein |
| A0A143ZZV5\|A0A143ZZV5 | Uncharacterized protein |
| A0A143ZVL3\|A0A143ZVL3 | Uncharacterized protein |
| A0A143ZXM1\|A0A143ZXM1 | Uncharacterized protein |
| A0A143ZWH7\|A0A143ZWH7 | Uncharacterized protein |
| C0H4S9\|C0H4S9 | Uncharacterized protein |
| A0A143ZWS9\|A0A143ZWS9 | Uncharacterized protein |
| Q8IAU4\|Q8IAU4 | Uncharacterized protein |
| Q8IAV1\|Q8IAV1 | Uncharacterized protein |
| Q8IAV3\|Q8IAV3 | Uncharacterized protein |
| Q8IAV5\|Q8IAV5 | Uncharacterized protein |
| C0H4U3\|C0H4U3 | Uncharacterized protein |
| A0A5K1K7Z9\|A0A5K1K7Z9 | Uncharacterized protein |
| Q8IAW6\|Q8IAW6 | Uncharacterized protein |
| C0H4U5\|C0H4U5 | Uncharacterized protein |
| C0H4U6\|C0H4U6 | Uncharacterized protein |
| Q8IAY2\|Q8IAY2 | Uncharacterized protein |
| Q8IAZ2\|Q8IAZ2 | Uncharacterized protein |
| C0H4U8\|C0H4U8 | Uncharacterized protein |
| Q8IB08\|Q8IB08 | Uncharacterized protein |
| Q8IB11\|Q8IB11 | Uncharacterized protein |
| Q8IB13\|Q8IB13 | Uncharacterized protein |
| C0H4V9\|C0H4V9 | Uncharacterized protein |
| C0H4W1\|C0H4W1 | Uncharacterized protein |
| Q8IB39\|Q8IB39 | Uncharacterized protein |
| Q8IB42\|Q8IB42 | Uncharacterized protein |
| Q8IB43\|Q8IB43 | Uncharacterized protein |
| Q8IB44\|Q8IB44 | Uncharacterized protein |
| Q8IB46\|Q8IB46 | Uncharacterized protein |
| Q8IB48\|Q8IB48 | Uncharacterized protein |
| C0H4W7\|C0H4W7 | Uncharacterized protein |
| C0H4W9\|C0H4W9 | Uncharacterized protein |
| C0H4X4\|C0H4X4 | Uncharacterized protein |
| Q8IB79\|Q8IB79 | Uncharacterized protein |
| Q8IB82\|Q8IB82 | Uncharacterized protein |
| Q8IB83\|Q8IB83 | Uncharacterized protein |
| C0H4X7\|C0H4X7 | Uncharacterized protein |
| C0H4X9\|C0H4X9 | Uncharacterized protein |
| Q8IB93\|Q8IB93 | Uncharacterized protein |
| Q8IBA6\|Q8IBA6 | Uncharacterized protein |
| Q8IBA8\|Q8IBA8 | Uncharacterized protein |
| A0A5K1K8A0\|A0A5K1K8A0 | Uncharacterized protein |
| Q8IBC0\|Q8IBC0 | Uncharacterized protein |
| Q8IBC1\|Q8IBC1 | Uncharacterized protein |
| Q8IBD3\|Q8IBD3 | Uncharacterized protein |
| C0H512\|C0H512 | Uncharacterized protein |
| Q8I3B9\|Q8I3B9 | Uncharacterized protein |
| Q8I3B8\|Q8I3B8 | Uncharacterized protein |
| A0A143ZVB6\|A0A143ZVB6 | Uncharacterized protein |
| Q8I399\|Q8I399 | Uncharacterized protein |
| Q8I394\|Q8I394 | Uncharacterized protein |
| Q8I387\|Q8I387 | Uncharacterized protein |
| C0H522\|C0H522 | Uncharacterized protein |
| A0A146M145\|A0A146M145 | Uncharacterized protein |
| C0H523\|C0H523 | Uncharacterized protein |
| Q8I376\|Q8I376 | Uncharacterized protein |
| Q8I369\|Q8I369 | Uncharacterized protein |
| Q8I367\|Q8I367 | Uncharacterized protein |
| Q8I363\|Q8I363 | Uncharacterized protein |
| Q8I356\|Q8I356 | Uncharacterized protein |
| Q8I340\|Q8I340 | Uncharacterized protein |
| C0H534\|C0H534 | Uncharacterized protein |
| Q8I334\|Q8I334 | Uncharacterized protein |
| Q8I332\|Q8I332 | Uncharacterized protein |
| Q8I325\|Q8I325 | Uncharacterized protein |
| Q8I319\|Q8I319 | Uncharacterized protein |
| Q8I316\|Q8I316 | Uncharacterized protein |
| Q8I315\|Q8I315 | Uncharacterized protein |
| Q8I314\|Q8I314 | Uncharacterized protein |
| Q8I308\|Q8I308 | Uncharacterized protein |
| Q8I307\|Q8I307 | Uncharacterized protein |
| Q8I2Z7\|Q8I2Z7 | Uncharacterized protein |
| Q8I2Z6\|Q8I2Z6 | Uncharacterized protein |
| Q8I2Z0\|Q8I2Z0 | Uncharacterized protein |
| Q8I2Y9\|Q8I2Y9 | Uncharacterized protein |
| C0H539\|C0H539 | Uncharacterized protein |
| C0H541\|C0H541 | Uncharacterized protein |
| Q8I2Y1\|Q8I2Y1 | Uncharacterized protein |
| Q8I2Y0\|Q8I2Y0 | Uncharacterized protein |
| Q8I2X8\|Q8I2X8 | Uncharacterized protein |
| Q8I2V0\|Q8I2V0 | Uncharacterized protein |
| Q8I2U7\|Q8I2U7 | Uncharacterized protein |
| C0H547\|C0H547 | Uncharacterized protein |
| C0H548\|C0H548 | Uncharacterized protein |
| Q8I2T8\|Q8I2T8 | Uncharacterized protein |
| Q8I2T6\|Q8I2T6 | Uncharacterized protein |
| Q8I2T4\|Q8I2T4 | Uncharacterized protein |
| Q8I2S2\|Q8I2S2 | Uncharacterized protein |
| C0H554\|C0H554 | Uncharacterized protein |
| Q8I2R6\|Q8I2R6 | Uncharacterized protein |
| C0H557\|C0H557 | Uncharacterized protein |
| C0H560\|C0H560 | Uncharacterized protein |
| C0H562\|C0H562 | Uncharacterized protein |
| Q8I2N8\|Q8I2N8 | Uncharacterized protein |
| C0H569\|C0H569 | Uncharacterized protein |
| Q8I2M5\|Q8I2M5 | Uncharacterized protein |
| Q8I2M4\|Q8I2M4 | Uncharacterized protein |
| Q8I2M2\|Q8I2M2 | Uncharacterized protein |
| Q8I2L9\|Q8I2L9 | Uncharacterized protein |
| A0A143ZXG1\|A0A143ZXG1 | Uncharacterized protein |
| C0H573\|C0H573 | Uncharacterized protein |
| C0H574\|C0H574 | Uncharacterized protein |
| Q8I2K7\|Q8I2K7 | Uncharacterized protein |
| C0H580\|C0H580 | Uncharacterized protein |
| C0H583\|C0H583 | Uncharacterized protein |
| C0H587\|C0H587 | Uncharacterized protein |
| C0H589\|C0H589 | Uncharacterized protein |
| Q8I2H6\|Q8I2H6 | Uncharacterized protein |
| C0H591\|C0H591 | Uncharacterized protein |
| Q8I2G3\|Q8I2G3 | Uncharacterized protein |
| Q8I2E9\|Q8I2E9 | Uncharacterized protein |
| Q8IK27\|Q8IK27 | Uncharacterized protein |
| Q8IK11\|Q8IK11 | Uncharacterized protein |
| Q8IK07\|Q8IK07 | Uncharacterized protein |
| Q8IK06\|Q8IK06 | Uncharacterized protein |
| C6S3C0\|C6S3C0 | Uncharacterized protein |
| Q8IJZ2\|Q8IJZ2 | Uncharacterized protein |
| Q8IJZ0\|Q8IJZ0 | Uncharacterized protein |
| Q8IJY8\|Q8IJY8 | Uncharacterized protein |
| C6S3C1\|C6S3C1 | Uncharacterized protein |
| Q8IJY5\|Q8IJY5 | Uncharacterized protein |
| Q8IJY2\|Q8IJY2 | Uncharacterized protein |
| Q8IJY1\|Q8IJY1 | Uncharacterized protein |
| A0A143ZX62\|A0A143ZX62 | Uncharacterized protein |
| Q8IJW7\|Q8IJW7 | Uncharacterized protein |
| Q8IJV4\|Q8IJV4 | Uncharacterized protein |
| Q8IJU7\|Q8IJU7 | Uncharacterized protein |
| Q8IJT6\|Q8IJT6 | Uncharacterized protein |
| Q8IJT4\|Q8IJT4 | Uncharacterized protein |
| Q8IJT0\|Q8IJT0 | Uncharacterized protein |
| Q8IJS4\|Q8IJS4 | Uncharacterized protein |
| Q8IJQ9\|Q8IJQ9 | Uncharacterized protein |
| Q8IJQ8\|Q8IJQ8 | Uncharacterized protein |
| Q8IJQ0\|Q8IJQ0 | Uncharacterized protein |
| Q8IJN5\|Q8IJN5 | Uncharacterized protein |
| Q8IJM1\|Q8IJM1 | Uncharacterized protein |
| Q8IJL7\|Q8IJL7 | Uncharacterized protein |
| Q8IJL3\|Q8IJL3 | Uncharacterized protein |
| Q8IJJ3\|Q8IJJ3 | Uncharacterized protein |
| Q8IJJ1\|Q8IJJ1 | Uncharacterized protein |
| Q8IJI2\|Q8IJI2 | Uncharacterized protein |
| Q8IJH2\|Q8IJH2 | Uncharacterized protein |
| Q8IJH0\|Q8IJH0 | Uncharacterized protein |
| Q8IJG5\|Q8IJG5 | Uncharacterized protein |
| Q8IJG1\|Q8IJG1 | Uncharacterized protein |
| Q8IJG0\|Q8IJG0 | Uncharacterized protein |
| Q8IJF7\|Q8IJF7 | Uncharacterized protein |
| Q8IJF5\|Q8IJF5 | Uncharacterized protein |
| Q8IJF2\|Q8IJF2 | Uncharacterized protein |
| Q8IJF0\|Q8IJF0 | Uncharacterized protein |
| Q8IJE5\|Q8IJE5 | Uncharacterized protein |
| C6S3D5\|C6S3D5 | Uncharacterized protein |
| Q8IJD9\|Q8IJD9 | Uncharacterized protein |
| C6S3D6\|C6S3D6 | Uncharacterized protein |
| Q8IJC8\|Q8IJC8 | Uncharacterized protein |
| Q8IJB8\|Q8IJB8 | Uncharacterized protein |
| Q8IJB6\|Q8IJB6 | Uncharacterized protein |
| Q8IJB4\|Q8IJB4 | Uncharacterized protein |
| Q8IJB2\|Q8IJB2 | Uncharacterized protein |
| Q8IJA8\|Q8IJA8 | Uncharacterized protein |
| Q8IJA2\|Q8IJA2 | Uncharacterized protein |
| Q8IJ95\|Q8IJ95 | Uncharacterized protein |
| Q8IJ89\|Q8IJ89 | Uncharacterized protein |
| C6S3E0\|C6S3E0 | Uncharacterized protein |
| Q8IJ84\|Q8IJ84 | Uncharacterized protein |
| Q8IJ80\|Q8IJ80 | Uncharacterized protein |
| Q8IJ75\|Q8IJ75 | Uncharacterized protein |
| C6S3E2\|C6S3E2 | Uncharacterized protein |
| Q8IJ65\|Q8IJ65 | Uncharacterized protein |
| A0A143ZYJ4\|A0A143ZYJ4 | Uncharacterized protein |
| Q8IJ58\|Q8IJ58 | Uncharacterized protein |
| Q8IJ43\|Q8IJ43 | Uncharacterized protein |
| Q8IJ42\|Q8IJ42 | Uncharacterized protein |
| A0A143ZYL5\|A0A143ZYL5 | Uncharacterized protein |
| Q8IJ33\|Q8IJ33 | Uncharacterized protein |
| C6S3E3\|C6S3E3 | Uncharacterized protein |
| C6S3E4\|C6S3E4 | Uncharacterized protein |
| Q8IJ25\|Q8IJ25 | Uncharacterized protein |
| A0A143ZY51\|A0A143ZY51 | Uncharacterized protein |
| Q8IIX7\|Q8IIX7 | Uncharacterized protein |
| Q8IIX4\|Q8IIX4 | Uncharacterized protein |
| Q8IIX2\|Q8IIX2 | Uncharacterized protein |
| Q8IIX1\|Q8IIX1 | Uncharacterized protein |
| Q8IIV9\|Q8IIV9 | Uncharacterized protein |
| Q8IIU9\|Q8IIU9 | Uncharacterized protein |
| Q8IIU5\|Q8IIU5 | Uncharacterized protein |
| Q8IIU4\|Q8IIU4 | Uncharacterized protein |
| Q8IIU1\|Q8IIU1 | Uncharacterized protein |
| Q8IIT9\|Q8IIT9 | Uncharacterized protein |
| Q8IIT8\|Q8IIT8 | Uncharacterized protein |
| Q8IIT4\|Q8IIT4 | Uncharacterized protein |
| Q8IIT1\|Q8IIT1 | Uncharacterized protein |
| Q8IIR5\|Q8IIR5 | Uncharacterized protein |
| C6S3E7\|C6S3E7 | Uncharacterized protein |
| C6S3E8\|C6S3E8 | Uncharacterized protein |
| C6S3E9\|C6S3E9 | Uncharacterized protein |
| Q8IIP6\|Q8IIP6 | Uncharacterized protein |
| Q8IIP3\|Q8IIP3 | Uncharacterized protein |
| Q8IIP0\|Q8IIP0 | Uncharacterized protein |
| Q8IIN0\|Q8IIN0 | Uncharacterized protein |
| Q8IIM4\|Q8IIM4 | Uncharacterized protein |
| Q8IIL7\|Q8IIL7 | Uncharacterized protein |
| Q8IIL3\|Q8IIL3 | Uncharacterized protein |
| Q8IIL2\|Q8IIL2 | Uncharacterized protein |
| A0A144A0T9\|A0A144A0T9 | Uncharacterized protein |
| Q8IIJ3\|Q8IIJ3 | Uncharacterized protein |
| Q8III8\|Q8III8 | Uncharacterized protein |
| Q8IIH8\|Q8IIH8 | Uncharacterized protein |
| A0A143ZYQ2\|A0A143ZYQ2 | Uncharacterized protein |
| A0A143ZYR6\|A0A143ZYR6 | Uncharacterized protein |
| Q8IIF9\|Q8IIF9 | Uncharacterized protein |
| Q8IIF5\|Q8IIF5 | Uncharacterized protein |
| Q8IIF1\|Q8IIF1 | Uncharacterized protein |
| Q8IIE6\|Q8IIE6 | Uncharacterized protein |
| Q8IIE2\|Q8IIE2 | Uncharacterized protein |
| Q8IIE1\|Q8IIE1 | Uncharacterized protein |
| Q8IID6\|Q8IID6 | Uncharacterized protein |
| Q8IIC0\|Q8IIC0 | Uncharacterized protein |
| Q8IIB3\|Q8IIB3 | Uncharacterized protein |
| C6S3G9\|C6S3G9 | Uncharacterized protein |
| Q8II91\|Q8II91 | Uncharacterized protein |
| Q8II89\|Q8II89 | Uncharacterized protein |
| Q8II84\|Q8II84 | Uncharacterized protein |
| Q8II83\|Q8II83 | Uncharacterized protein |
| A0A143ZYW1\|A0A143ZYW1 | Uncharacterized protein |
| Q8II66\|Q8II66 | Uncharacterized protein |
| Q8II65\|Q8II65 | Uncharacterized protein |
| Q8II55\|Q8II55 | Uncharacterized protein |
| Q8II51\|Q8II51 | Uncharacterized protein |
| Q8II50\|Q8II50 | Uncharacterized protein |
| Q8II49\|Q8II49 | Uncharacterized protein |
| A0A144A2V3\|A0A144A2V3 | Uncharacterized protein |
| Q8II46\|Q8II46 | Uncharacterized protein |
| Q8II34\|Q8II34 | Uncharacterized protein |
| Q8II33\|Q8II33 | Uncharacterized protein |
| Q8II30\|Q8II30 | Uncharacterized protein |
| Q8II29\|Q8II29 | Uncharacterized protein |
| Q8II22\|Q8II22 | Uncharacterized protein |
| Q8II20\|Q8II20 | Uncharacterized protein |
| Q8II15\|Q8II15 | Uncharacterized protein |
| Q8II12\|Q8II12 | Uncharacterized protein |
| A0A143ZYI9\|A0A143ZYI9 | Uncharacterized protein |
| C6S3F6\|C6S3F6 | Uncharacterized protein |
| Q8II06\|Q8II06 | Uncharacterized protein |
| Q8IHZ8\|Q8IHZ8 | Uncharacterized protein |
| Q8IHZ7\|Q8IHZ7 | Uncharacterized protein |
| Q8IHY4\|Q8IHY4 | Uncharacterized protein |
| Q8IHY2\|Q8IHY2 | Uncharacterized protein |
| Q8IHX0\|Q8IHX0 | Uncharacterized protein |
| Q8IHW8\|Q8IHW8 | Uncharacterized protein |
| Q8IHW3\|Q8IHW3 | Uncharacterized protein |
| Q8IHW1\|Q8IHW1 | Uncharacterized protein |
| Q8IHV6\|Q8IHV6 | Uncharacterized protein |
| Q8IHV5\|Q8IHV5 | Uncharacterized protein |
| Q8IHV2\|Q8IHV2 | Uncharacterized protein |
| Q8IHV0\|Q8IHV0 | Uncharacterized protein |
| Q8IHU2\|Q8IHU2 | Uncharacterized protein |
| Q8IHT6\|Q8IHT6 | Uncharacterized protein |
| Q8IHR9\|Q8IHR9 | Uncharacterized protein |
| Q8IHR0\|Q8IHR0 | Uncharacterized protein |
| Q8IHQ7\|Q8IHQ7 | Uncharacterized protein |
| Q8IHQ6\|Q8IHQ6 | Uncharacterized protein |
| A0A143ZZG7\|A0A143ZZG7 | Uncharacterized protein |
| C6S3G8\|C6S3G8 | Uncharacterized protein |
| Q8IHN5\|Q8IHN5 | Uncharacterized protein |
| Q8IHM9\|Q8IHM9 | Uncharacterized protein |
| Q8I628\|Q8I628 | Uncharacterized protein |
| A0A144A0J9\|A0A144A0J9 | Uncharacterized protein |
| Q8I618\|Q8I618 | Uncharacterized protein |
| Q8I614\|Q8I614 | Uncharacterized protein |
| A0A144A114\|A0A144A114 | Uncharacterized protein |
| Q8I600\|Q8I600 | Uncharacterized protein |
| Q8I5Z8\|Q8I5Z8 | Uncharacterized protein |
| Q8I5Z6\|Q8I5Z6 | Uncharacterized protein |
| A0A144A3J2\|A0A144A3J2 | Uncharacterized protein |
| A0A143ZZL4\|A0A143ZZL4 | Uncharacterized protein |
| Q8I5Y6\|Q8I5Y6 | Uncharacterized protein |
| A0A143ZZM5\|A0A143ZZM5 | Uncharacterized protein |
| Q8I5X7\|Q8I5X7 | Uncharacterized protein |
| Q8I5X1\|Q8I5X1 | Uncharacterized protein |
| A0A143ZZG4\|A0A143ZZG4 | Uncharacterized protein |
| Q8I5W6\|Q8I5W6 | Uncharacterized protein |
| Q8I5U6\|Q8I5U6 | Uncharacterized protein |
| Q8I5U4\|Q8I5U4 | Uncharacterized protein |
| Q8I5T8\|Q8I5T8 | Uncharacterized protein |
| Q8I5T0\|Q8I5T0 | Uncharacterized protein |
| Q8I5R5\|Q8I5R5 | Uncharacterized protein |
| Q8I5Q9\|Q8I5Q9 | Uncharacterized protein |
| Q8I5Q8\|Q8I5Q8 | Uncharacterized protein |
| Q8I5Q7\|Q8I5Q7 | Uncharacterized protein |
| Q8I5Q2\|Q8I5Q2 | Uncharacterized protein |
| C6S3K6\|C6S3K6 | Uncharacterized protein |
| Q8I5Q1\|Q8I5Q1 | Uncharacterized protein |
| Q8I5P6\|Q8I5P6 | Uncharacterized protein |
| Q8I5N8\|Q8I5N8 | Uncharacterized protein |
| Q8I5N1\|Q8I5N1 | Uncharacterized protein |
| C6S3K7\|C6S3K7 | Uncharacterized protein |
| Q8I5K7\|Q8I5K7 | Uncharacterized protein |
| A0A144A1U0\|A0A144A1U0 | Uncharacterized protein |
| Q8I5K1\|Q8I5K1 | Uncharacterized protein |
| Q8I5J5\|Q8I5J5 | Uncharacterized protein |
| Q8I5J4\|Q8I5J4 | Uncharacterized protein |
| Q8I5I6\|Q8I5I6 | Uncharacterized protein |
| Q8I5I5\|Q8I5I5 | Uncharacterized protein |
| A0A144A125\|A0A144A125 | Uncharacterized protein |
| A0A143ZZX1\|A0A143ZZX1 | Uncharacterized protein |
| Q8I5H6\|Q8I5H6 | Uncharacterized protein |
| Q8I5H0\|Q8I5H0 | Uncharacterized protein |
| Q8I5G9\|Q8I5G9 | Uncharacterized protein |
| Q8I5G7\|Q8I5G7 | Uncharacterized protein |
| Q8I5G5\|Q8I5G5 | Uncharacterized protein |
| Q8I5G4\|Q8I5G4 | Uncharacterized protein |
| A0A143ZZZ3\|A0A143ZZZ3 | Uncharacterized protein |
| Q8I5F8\|Q8I5F8 | Uncharacterized protein |
| Q8I5F5\|Q8I5F5 | Uncharacterized protein |
| Q8I5E8\|Q8I5E8 | Uncharacterized protein |
| Q8I5E5\|Q8I5E5 | Uncharacterized protein |
| Q8I5E2\|Q8I5E2 | Uncharacterized protein |
| Q8I5D8\|Q8I5D8 | Uncharacterized protein |
| Q8I5D6\|Q8I5D6 | Uncharacterized protein |
| Q8I5D4\|Q8I5D4 | Uncharacterized protein |
| Q8I5C9\|Q8I5C9 | Uncharacterized protein |
| Q8I5C8\|Q8I5C8 | Uncharacterized protein |
| Q8I5C3\|Q8I5C3 | Uncharacterized protein |
| C6S3K8\|C6S3K8 | Uncharacterized protein |
| C6S3K9\|C6S3K9 | Uncharacterized protein |
| Q8I596\|Q8I596 | Uncharacterized protein |
| A0A144A4C1\|A0A144A4C1 | Uncharacterized protein |
| A0A144A1U3\|A0A144A1U3 | Uncharacterized protein |
| Q8I591\|Q8I591 | Uncharacterized protein |
| Q8I584\|Q8I584 | Uncharacterized protein |
| Q8I582\|Q8I582 | Uncharacterized protein |
| Q8I571\|Q8I571 | Uncharacterized protein |
| Q8I559\|Q8I559 | Uncharacterized protein |
| Q8I554\|Q8I554 | Uncharacterized protein |
| Q8I552\|Q8I552 | Uncharacterized protein |
| Q8I548\|Q8I548 | Uncharacterized protein |
| Q8I538\|Q8I538 | Uncharacterized protein |
| Q8I517\|Q8I517 | Uncharacterized protein |
| Q8I508\|Q8I508 | Uncharacterized protein |
| Q8I506\|Q8I506 | Uncharacterized protein |
| Q8I504\|Q8I504 | Uncharacterized protein |
| Q8I4Z7\|Q8I4Z7 | Uncharacterized protein |
| Q8I4Y4\|Q8I4Y4 | Uncharacterized protein |
| Q8I4Y2\|Q8I4Y2 | Uncharacterized protein |
| Q8I4X9\|Q8I4X9 | Uncharacterized protein |
| A0A144A322\|A0A144A322 | Uncharacterized protein |
| Q8I4X2\|Q8I4X2 | Uncharacterized protein |
| Q8I4W1\|Q8I4W1 | Uncharacterized protein |
| Q8I4V0\|Q8I4V0 | Uncharacterized protein |
| Q8I4U8\|Q8I4U8 | Uncharacterized protein |
| Q8I4U7\|Q8I4U7 | Uncharacterized protein |
| Q8I4U6\|Q8I4U6 | Uncharacterized protein |
| Q8I4U4\|Q8I4U4 | Uncharacterized protein |
| Q8I4U3\|Q8I4U3 | Uncharacterized protein |
| Q8I4S0\|Q8I4S0 | Uncharacterized protein |
| Q8I4R8\|Q8I4R8 | Uncharacterized protein |
| Q8I4R6\|Q8I4R6 | Uncharacterized protein |
| Q8I4R3\|Q8I4R3 | Uncharacterized protein |
| Q8I4Q2\|Q8I4Q2 | Uncharacterized protein |
| Q8IEI7\|Q8IEI7 | Uncharacterized protein |
| C0H595\|C0H595 | Uncharacterized protein |
| C0H596\|C0H596 | Uncharacterized protein |
| Q8IET6\|Q8IET6 | Uncharacterized protein |
| Q8IES9\|Q8IES9 | Uncharacterized protein |
| Q8IES2\|Q8IES2 | Uncharacterized protein |
| Q8IEQ8\|Q8IEQ8 | Uncharacterized protein |
| C0H5A1\|C0H5A1 | Uncharacterized protein |
| Q8IEQ3\|Q8IEQ3 | Uncharacterized protein |
| Q8IEP8\|Q8IEP8 | Uncharacterized protein |
| Q8IEP1\|Q8IEP1 | Uncharacterized protein |
| Q8IEP0\|Q8IEP0 | Uncharacterized protein |
| C0H5A5\|C0H5A5 | Uncharacterized protein |
| C0H5A6\|C0H5A6 | Uncharacterized protein |
| C0H5A7\|C0H5A7 | Uncharacterized protein |
| Q8IEM7\|Q8IEM7 | Uncharacterized protein |
| Q8IEM4\|Q8IEM4 | Uncharacterized protein |
| Q8IEL7\|Q8IEL7 | Uncharacterized protein |
| Q8IEL6\|Q8IEL6 | Uncharacterized protein |
| C0H5A9\|C0H5A9 | Uncharacterized protein |
| Q8IEJ9\|Q8IEJ9 | Uncharacterized protein |
| C0H5B0\|C0H5B0 | Uncharacterized protein |
| Q8IEH8\|Q8IEH8 | Uncharacterized protein |
| Q8IEH4\|Q8IEH4 | Uncharacterized protein |
| Q8IEH3\|Q8IEH3 | Uncharacterized protein |
| Q8IEG5\|Q8IEG5 | Uncharacterized protein |
| Q8IEG2\|Q8IEG2 | Uncharacterized protein |
| Q8IEF9\|Q8IEF9 | Uncharacterized protein |
| C0H5B9\|C0H5B9 | Uncharacterized protein |
| C0H5C0\|C0H5C0 | Uncharacterized protein |
| Q8IEE1\|Q8IEE1 | Uncharacterized protein |
| C0H5C1\|C0H5C1 | Uncharacterized protein |
| Q8IED9\|Q8IED9 | Uncharacterized protein |
| Q8IED6\|Q8IED6 | Uncharacterized protein |
| Q8IED3\|Q8IED3 | Uncharacterized protein |
| Q8IEC6\|Q8IEC6 | Uncharacterized protein |
| A0A5K1K826\|A0A5K1K826 | Uncharacterized protein |
| Q8IEC1\|Q8IEC1 | Uncharacterized protein |
| Q8IEB8\|Q8IEB8 | Uncharacterized protein |
| Q8IEA7\|Q8IEA7 | Uncharacterized protein |
| A0A5K1K8Q7\|A0A5K1K8Q7 | Uncharacterized protein |
| Q8IEA2\|Q8IEA2 | Uncharacterized protein |
| A0A5K1K836\|A0A5K1K836 | Uncharacterized protein |
| C0H5D2\|C0H5D2 | Uncharacterized protein |
| Q8IE81\|Q8IE81 | Uncharacterized protein |
| Q8IE76\|Q8IE76 | Uncharacterized protein |
| C0H5D5\|C0H5D5 | Uncharacterized protein |
| A0A5K1K9E9\|A0A5K1K9E9 | Uncharacterized protein |
| Q8IE58\|Q8IE58 | Uncharacterized protein |
| C0H5E0\|C0H5E0 | Uncharacterized protein |
| A0A5K1K8S6\|A0A5K1K8S6 | Uncharacterized protein |
| Q8IE43\|Q8IE43 | Uncharacterized protein |
| A0A5K1K8J1\|A0A5K1K8J1 | Uncharacterized protein |
| A0A5K1K8T4\|A0A5K1K8T4 | Uncharacterized protein |
| A0A5K1K8T3\|A0A5K1K8T3 | Uncharacterized protein |
| A0A5K1K9F1\|A0A5K1K9F1 | Uncharacterized protein |
| A0A5K1K8U3\|A0A5K1K8U3 | Uncharacterized protein |
| A0A5K1K984\|A0A5K1K984 | Uncharacterized protein |
| A0A5K1K8J9\|A0A5K1K8J9 | Uncharacterized protein |
| Q8IDZ3\|Q8IDZ3 | Uncharacterized protein |
| A0A5K1K8V1\|A0A5K1K8V1 | Uncharacterized protein |
| Q8IDX4\|Q8IDX4 | Uncharacterized protein |
| Q8IDX1\|Q8IDX1 | Uncharacterized protein |
| C0H5F6\|C0H5F6 | Uncharacterized protein |
| Q8IDW9\|Q8IDW9 | Uncharacterized protein |
| Q8IDW6\|Q8IDW6 | Uncharacterized protein |
| A0A5K1K8U7\|A0A5K1K8U7 | Uncharacterized protein |
| A0A5K1K989\|A0A5K1K989 | Uncharacterized protein |
| A0A5K1K898\|A0A5K1K898 | Uncharacterized protein |
| A0A5K1K8M0\|A0A5K1K8M0 | Uncharacterized protein |
| A0A5K1K8Y9\|A0A5K1K8Y9 | Uncharacterized protein |
| A0A5K1K8I2\|A0A5K1K8I2 | Uncharacterized protein |
| A0A5K1K8W6\|A0A5K1K8W6 | Uncharacterized protein |
| A0A5K1K969\|A0A5K1K969 | Uncharacterized protein |
| C0H5G1\|C0H5G1 | Uncharacterized protein |
| A0A5K1K8W7\|A0A5K1K8W7 | Uncharacterized protein |
| A0A5K1K9F5\|A0A5K1K9F5 | Uncharacterized protein |
| A0A5K1K8X3\|A0A5K1K8X3 | Uncharacterized protein |
| Q8IDQ8\|Q8IDQ8 | Uncharacterized protein |
| A0A5K1K8C4\|A0A5K1K8C4 | Uncharacterized protein |
| A0A5K1K8J8\|A0A5K1K8J8 | Uncharacterized protein |
| C0H5H3\|C0H5H3 | Uncharacterized protein |
| A0A5K1K9F6\|A0A5K1K9F6 | Uncharacterized protein |
| A0A5K1K8P9\|A0A5K1K8P9 | Uncharacterized protein |
| A0A5K1K9F7\|A0A5K1K9F7 | Uncharacterized protein |
| A0A5K1K9A2\|A0A5K1K9A2 | Uncharacterized protein |
| A0A5K1K8R0\|A0A5K1K8R0 | Uncharacterized protein |
| A0A5K1K8Z2\|A0A5K1K8Z2 | Uncharacterized protein |
| A0A5K1K8Y7\|A0A5K1K8Y7 | Uncharacterized protein |
| A0A5K1K9F8\|A0A5K1K9F8 | Uncharacterized protein |
| A0A5K1K8Z5\|A0A5K1K8Z5 | Uncharacterized protein |
| A0A5K1K985\|A0A5K1K985 | Uncharacterized protein |
| A0A5K1K8F9\|A0A5K1K8F9 | Uncharacterized protein |
| A0A5K1K8S2\|A0A5K1K8S2 | Uncharacterized protein |
| Q8IDI8\|Q8IDI8 | Uncharacterized protein |
| A0A5K1K904\|A0A5K1K904 | Uncharacterized protein |
| A0A143ZVD1\|A0A143ZVD1 | Uncharacterized protein |
| Q8IDH7\|Q8IDH7 | Uncharacterized protein |
| A0A5K1K907\|A0A5K1K907 | Uncharacterized protein |
| Q8IDF4\|Q8IDF4 | Uncharacterized protein |
| Q8IDF2\|Q8IDF2 | Uncharacterized protein |
| Q8IDD7\|Q8IDD7 | Uncharacterized protein |
| C0H5I6\|C0H5I6 | Uncharacterized protein |
| C0H5I8\|C0H5I8 | Uncharacterized protein |
| C0H5J0\|C0H5J0 | Uncharacterized protein |
| A0A5K1K8P5\|A0A5K1K8P5 | Uncharacterized protein |
| A0A5K1K912\|A0A5K1K912 | Uncharacterized protein |
| A0A5K1K918\|A0A5K1K918 | Uncharacterized protein |
| A0A5K1K9B5\|A0A5K1K9B5 | Uncharacterized protein |
| A0A5K1K954\|A0A5K1K954 | Uncharacterized protein |
| A0A5K1K9G3\|A0A5K1K9G3 | Uncharacterized protein |
| A0A5K1K8L4\|A0A5K1K8L4 | Uncharacterized protein |
| A0A5K1K8X0\|A0A5K1K8X0 | Uncharacterized protein |
| C0H5K6\|C0H5K6 | Uncharacterized protein |
| A0A5K1K935\|A0A5K1K935 | Uncharacterized protein |
| A0A5K1K961\|A0A5K1K961 | Uncharacterized protein |
| Q8ID49\|Q8ID49 | Uncharacterized protein |
| A0A5K1K8S1\|A0A5K1K8S1 | Uncharacterized protein |
| Q8ID41\|Q8ID41 | Uncharacterized protein |
| Q8ID34\|Q8ID34 | Uncharacterized protein |
| C0H5L2\|C0H5L2 | Uncharacterized protein |
| C0H5L3\|C0H5L3 | Uncharacterized protein |
| Q8ID17\|Q8ID17 | Uncharacterized protein |
| C0H5L6\|C0H5L6 | Uncharacterized protein |
| A0A146M1M7\|A0A146M1M7 | Uncharacterized protein |
| Q8I4N8\|Q8I4N8 | Uncharacterized protein |
| Q8IM76\|Q8IM76 | Uncharacterized protein |
| Q8IM72\|Q8IM72 | Uncharacterized protein |
| Q8IM69\|Q8IM69 | Uncharacterized protein |
| C6S3H1\|C6S3H1 | Uncharacterized protein |
| Q8IM61\|Q8IM61 | Uncharacterized protein |
| Q8IM52\|Q8IM52 | Uncharacterized protein |
| Q8IM44\|Q8IM44 | Uncharacterized protein |
| Q8IM22\|Q8IM22 | Uncharacterized protein |
| Q8IM20\|Q8IM20 | Uncharacterized protein |
| Q8IM17\|Q8IM17 | Uncharacterized protein |
| Q8IM11\|Q8IM11 | Uncharacterized protein |
| Q8IM01\|Q8IM01 | Uncharacterized protein |
| A0A144A3L0\|A0A144A3L0 | Uncharacterized protein |
| Q8ILZ0\|Q8ILZ0 | Uncharacterized protein |
| Q8ILY4\|Q8ILY4 | Uncharacterized protein |
| Q8ILX7\|Q8ILX7 | Uncharacterized protein |
| Q8ILW4\|Q8ILW4 | Uncharacterized protein |
| Q8ILW2\|Q8ILW2 | Uncharacterized protein |
| Q8ILV8\|Q8ILV8 | Uncharacterized protein |
| Q8ILV3\|Q8ILV3 | Uncharacterized protein |
| Q8ILU0\|Q8ILU0 | Uncharacterized protein |
| A0A144A2E6\|A0A144A2E6 | Uncharacterized protein |
| Q8ILT2\|Q8ILT2 | Uncharacterized protein |
| C6S3H6\|C6S3H6 | Uncharacterized protein |
| Q8ILR4\|Q8ILR4 | Uncharacterized protein |
| Q8ILR0\|Q8ILR0 | Uncharacterized protein |
| Q8ILQ5\|Q8ILQ5 | Uncharacterized protein |
| Q8ILQ4\|Q8ILQ4 | Uncharacterized protein |
| Q8ILQ3\|Q8ILQ3 | Uncharacterized protein |
| Q8ILN3\|Q8ILN3 | Uncharacterized protein |
| Q8ILN0\|Q8ILN0 | Uncharacterized protein |
| Q8ILM9\|Q8ILM9 | Uncharacterized protein |
| Q8ILK6\|Q8ILK6 | Uncharacterized protein |
| Q8ILK5\|Q8ILK5 | Uncharacterized protein |
| Q8ILJ6\|Q8ILJ6 | Uncharacterized protein |
| Q8ILJ4\|Q8ILJ4 | Uncharacterized protein |
| Q8ILJ1\|Q8ILJ1 | Uncharacterized protein |
| Q8ILJ0\|Q8ILJ0 | Uncharacterized protein |
| C6S3I3\|C6S3I3 | Uncharacterized protein |
| Q8ILH5\|Q8ILH5 | Uncharacterized protein |
| Q8ILC5\|Q8ILC5 | Uncharacterized protein |
| Q8ILC0\|Q8ILC0 | Uncharacterized protein |
| Q8ILB5\|Q8ILB5 | Uncharacterized protein |
| Q8ILA8\|Q8ILA8 | Uncharacterized protein |
| Q8ILA5\|Q8ILA5 | Uncharacterized protein |
| Q8ILA3\|Q8ILA3 | Uncharacterized protein |
| Q8IL99\|Q8IL99 | Uncharacterized protein |
| Q8IL95\|Q8IL95 | Uncharacterized protein |
| Q8IL93\|Q8IL93 | Uncharacterized protein |
| Q8IL90\|Q8IL90 | Uncharacterized protein |
| Q8IL82\|Q8IL82 | Uncharacterized protein |
| Q8IL61\|Q8IL61 | Uncharacterized protein |
| Q8IL59\|Q8IL59 | Uncharacterized protein |
| Q8IL40\|Q8IL40 | Uncharacterized protein |
| Q8IL31\|Q8IL31 | Uncharacterized protein |
| Q8IL27\|Q8IL27 | Uncharacterized protein |
| C6S3I7\|C6S3I7 | Uncharacterized protein |
| Q8IL24\|Q8IL24 | Uncharacterized protein |
| Q8IL18\|Q8IL18 | Uncharacterized protein |
| Q8IL16\|Q8IL16 | Uncharacterized protein |
| A0A144A260\|A0A144A260 | Uncharacterized protein |
| Q8IL00\|Q8IL00 | Uncharacterized protein |
| C6S3I8\|C6S3I8 | Uncharacterized protein |
| C6S3I9\|C6S3I9 | Uncharacterized protein |
| Q8IKZ8\|Q8IKZ8 | Uncharacterized protein |
| Q8IKZ4\|Q8IKZ4 | Uncharacterized protein |
| C6S3J0\|C6S3J0 | Uncharacterized protein |
| C6S3J1\|C6S3J1 | Uncharacterized protein |
| Q8IKX9\|Q8IKX9 | Uncharacterized protein |
| Q8IKX7\|Q8IKX7 | Uncharacterized protein |
| Q8IKX6\|Q8IKX6 | Uncharacterized protein |
| C6S3J2\|C6S3J2 | Uncharacterized protein |
| Q8IKV2\|Q8IKV2 | Uncharacterized protein |
| C6S3J3\|C6S3J3 | Uncharacterized protein |
| Q8IKT9\|Q8IKT9 | Uncharacterized protein |
| Q8IKT0\|Q8IKT0 | Uncharacterized protein |
| Q8IKR6\|Q8IKR6 | Uncharacterized protein |
| Q8IKR2\|Q8IKR2 | Uncharacterized protein |
| Q8IKQ6\|Q8IKQ6 | Uncharacterized protein |
| Q8IKQ4\|Q8IKQ4 | Uncharacterized protein |
| Q8IKP9\|Q8IKP9 | Uncharacterized protein |
| Q8IKN7\|Q8IKN7 | Uncharacterized protein |
| Q8IKN5\|Q8IKN5 | Uncharacterized protein |
| Q8IKM9\|Q8IKM9 | Uncharacterized protein |
| Q8IKM2\|Q8IKM2 | Uncharacterized protein |
| C6S3J8\|C6S3J8 | Uncharacterized protein |
| Q8IKL3\|Q8IKL3 | Uncharacterized protein |
| Q8IKL2\|Q8IKL2 | Uncharacterized protein |
| Q8IKK8\|Q8IKK8 | Uncharacterized protein |
| Q8IKJ6\|Q8IKJ6 | Uncharacterized protein |
| C6S3K0\|C6S3K0 | Uncharacterized protein |
| Q8IKI4\|Q8IKI4 | Uncharacterized protein |
| Q8IKI1\|Q8IKI1 | Uncharacterized protein |
| Q8IKH6\|Q8IKH6 | Uncharacterized protein |
| C6S3K1\|C6S3K1 | Uncharacterized protein |
| Q8IKG8\|Q8IKG8 | Uncharacterized protein |
| Q8IKG0\|Q8IKG0 | Uncharacterized protein |
| Q8IKF9\|Q8IKF9 | Uncharacterized protein |
| Q8IKE6\|Q8IKE6 | Uncharacterized protein |
| Q8IKE2\|Q8IKE2 | Uncharacterized protein |
| Q8IKD9\|Q8IKD9 | Uncharacterized protein |
| Q8IKD7\|Q8IKD7 | Uncharacterized protein |
| Q8IKD6\|Q8IKD6 | Uncharacterized protein |
| Q8IKD5\|Q8IKD5 | Uncharacterized protein |
| Q8IKD0\|Q8IKD0 | Uncharacterized protein |
| Q8IKC2\|Q8IKC2 | Uncharacterized protein |
| Q8IKC1\|Q8IKC1 | Uncharacterized protein |
| Q8IKB5\|Q8IKB5 | Uncharacterized protein |
| Q8IKA2\|Q8IKA2 | Uncharacterized protein |
| Q8IKA1\|Q8IKA1 | Uncharacterized protein |
| Q8IK87\|Q8IK87 | Uncharacterized protein |
| A0A144A7A0\|A0A144A7A0 | Uncharacterized protein |
| Q8IK78\|Q8IK78 | Uncharacterized protein |
| Q8IK71\|Q8IK71 | Uncharacterized protein |
| Q8IK70\|Q8IK70 | Uncharacterized protein |
| A0A144A4N4\|A0A144A4N4 | Uncharacterized protein |
| Q8IK54\|Q8IK54 | Uncharacterized protein |
| Q8IK46\|Q8IK46 | Uncharacterized protein |
| C6KSQ8\|C6KSQ8 | Uncharacterized protein |
| O96204\|O96204 | Uncharacterized protein |
| O97230\|O97230 | Uncharacterized protein |
| O97236\|O97236 | Uncharacterized protein |
| Q8I5U3\|Q8I5U3 | Uncharacterized protein |
| Q8I1T6\|Q8I1T6 | Uncharacterized protein |
| Q8I1Q4\|Q8I1Q4 | Uncharacterized protein |
| Q8I1P3\|Q8I1P3 | Uncharacterized protein |
| A0A5K1K8R2\|A0A5K1K8R2 | Uncharacterized protein |
| A0A143ZXJ2\|A0A143ZXJ2 | Uncharacterized protein |
| Q8I350\|Q8I350 | Uncharacterized protein |
| Q8I322\|Q8I322 | Uncharacterized protein |
| C0H536\|C0H536 | Uncharacterized protein |
| Q8IIF6\|Q8IIF6 | Uncharacterized protein |
| Q8IIL6\|Q8IIL6 | Uncharacterized protein |
| C6S3I5\|C6S3I5 | Uncharacterized protein |
| Q8ILQ6\|Q8ILQ6 | Uncharacterized protein |
| Q8ILX0\|Q8ILX0 | Uncharacterized protein |
| Q8IM09\|Q8IM09 | Uncharacterized protein |
| Q8I462\|Q8I462 | Uncharacterized protein |
| Q8I425\|Q8I425 | Uncharacterized protein |
| Q8I3W5\|Q8I3W5 | Uncharacterized protein |
| C6KT10\|C6KT10 | Uncharacterized protein |
| C6KT13\|C6KT13 | Uncharacterized protein |
| A0A5K1K942\|A0A5K1K942 | Uncharacterized protein |
| Q9NFA1\|Q9NFA1 | Uncharacterized protein |
| A0A143ZVL6\|A0A143ZVL6 | Uncharacterized protein |
| Q8IK94\|Q8IK94 | Uncharacterized protein |
| Q8IK96\|Q8IK96 | Uncharacterized protein |
| Q8IL70\|Q8IL70 | Uncharacterized protein |
| Q8IKW8\|Q8IKW8 | Uncharacterized protein |
| Q8IKS5\|Q8IKS5 | Uncharacterized protein |
| Q8IKD1\|Q8IKD1 | Uncharacterized protein |
| A0A5K1K8R5\|A0A5K1K8R5 | Uncharacterized protein |
| A0A5K1K8I1\|A0A5K1K8I1 | Uncharacterized protein |
| A0A5K1K8U2\|A0A5K1K8U2 | Uncharacterized protein |
| Q8IEK2\|Q8IEK2 | Uncharacterized protein |
| A0A5K1K8R1\|A0A5K1K8R1 | Uncharacterized protein |
| C0H4M8\|C0H4M8 | Uncharacterized protein |
| Q8IBV5\|Q8IBV5 | Uncharacterized protein |
| Q8I5N3\|Q8I5N3 | Uncharacterized protein |
| Q8I5D1\|Q8I5D1 | Uncharacterized protein |
| Q8I525\|Q8I525 | Uncharacterized protein |
| Q8I231\|Q8I231 | UMP-CMP kinase |
| O96196\|O96196 | UDP-N-acetylglucosamine transferase subunit ALG14 |
| Q8IDQ3\|Q8IDQ3 | UDP-N-acetylglucosamine pyrophosphorylase |
| Q8IIM9\|Q8IIM9 | UDP-galactose transporter |
| Q8IIJ6\|Q8IIJ6 | Ubiquitinyl hydrolase 1 OS |
| Q8I5F0\|Q8I5F0 | Ubiquitinyl hydrolase 1 OS |
| Q8IHY3\|Q8IHY3 | Ubiquitin-related modifier 1 homolog OS |
| O77341\|O77341 | Ubiquitin-protein ligase |
| Q8IEI4\|Q8IEI4 | Ubiquitin-like protein nedd8 homolog |
| C6S3K2\|C6S3K2 | Ubiquitin-like protein ATG12 OS |
| Q8I2T2\|Q8I2T2 | Ubiquitin-like protein |
| Q8IEG7\|Q8IEG7 | Ubiquitin-like protein |
| Q8I545\|Q8I545 | Ubiquitin-like modifier HUB1 |
| Q8IB31\|Q8IB31 | Ubiquitin-like domain-containing protein OS |
| A0A143ZYV5\|A0A143ZYV5 | Ubiquitin-like domain-containing protein OS |
| C6KSR0\|C6KSR0 | Ubiquitin-conjugating enzyme E2 PEX4 |
| Q8I607\|Q8I607 | Ubiquitin-conjugating enzyme E2 OS |
| Q8IDP1\|Q8IDP1 | Ubiquitin-conjugating enzyme E2 OS |
| Q8I3J4\|Q8I3J4 | Ubiquitin-conjugating enzyme E2 N |
| O97241\|O97241 | Ubiquitin-conjugating enzyme E2 |
| O77397\|O77397 | Ubiquitin-conjugating enzyme E2 |
| Q8IAW2\|Q8IAW2 | Ubiquitin-conjugating enzyme E2 |
| Q8IJ70\|Q8IJ70 | Ubiquitin-conjugating enzyme E2 |
| Q8ILW5\|Q8ILW5 | Ubiquitin-conjugating enzyme E2 |
| Q8IDZ6\|Q8IDZ6 | Ubiquitin-activating enzyme OS |
| Q8ID54\|Q8ID54 | Ubiquitin-activating enzyme OS |
| Q8I5F9\|Q8I5F9 | Ubiquitin-activating enzyme E1 OS |
| C0H5H9\|C0H5H9 | Ubiquitin-activating enzyme E1 |
| Q8I1Z5\|Q8I1Z5 | Ubiquitin specific protease |
| Q8IAS1\|Q8IAS1 | Ubiquitin regulatory protein |
| C0H4U7\|C0H4U7 | Ubiquitin OS |
| Q8ID50\|Q8ID50 | Ubiquitin OS |
| C0H540\|C0H540 | Ubiquitin fusion degradation protein 1 OS |
| Q8ILR6\|Q8ILR6 | Ubiquitin fusion degradation protein 1 |
| Q8I1U8\|Q8I1U8 | Ubiquitin carboxyl-terminal hydrolase OS |
| Q8I3J3\|Q8I3J3 | Ubiquitin carboxyl-terminal hydrolase OS |
| Q8IKM8\|Q8IKM8 | Ubiquitin carboxyl-terminal hydrolase isozyme L3 OS |
| Q8I296\|Q8I296 | Ubiquitin carboxyl-terminal hydrolase 1 |
| Q8IBJ1\|Q8IBJ1 | Ubiquitin carboxyl-terminal hydrolase |
| A0A143ZYI5\|A0A143ZYI5 | Ubiquinone biosynthesis protein COQ4 homolog |
| Q8IBK9\|Q8IBK9 | Ubiquinone biosynthesis O-methyltransferase |
| Q8I661\|Q8I661 | Ubiquinol-cytochrome-c reductase complex assembly factor 1 |
| C0H4Y9\|C0H4Y9 | U6 snRNA-associated Sm-like protein LSm8 OS |
| Q8IE68\|Q8IE68 | U6 snRNA-associated Sm-like protein LSm6 |
| Q8IL38\|Q8IL38 | U6 snRNA-associated Sm-like protein LSm5 OS |
| Q8IIT3\|Q8IIT3 | U6 snRNA-associated Sm-like protein LSm4 OS |
| C0H4W2\|C0H4W2 | U6 snRNA-associated Sm-like protein LSm3 OS |
| Q8I3Q7\|Q8I3Q7 | U6 snRNA-associated Sm-like protein LSm2 OS |
| Q8IIB9\|Q8IIB9 | U6 snRNA-associated Sm-like protein LSm1 OS |
| Q8I5V6\|Q8I5V6 | U6 snRNA-associated Sm-like protein LSm |
| A0A5K1K8P1\|A0A5K1K8P1 | U6 snRNA phosphodiesterase |
| Q8IJZ9\|Q8IJZ9 | U5 small nuclear ribonucleoprotein component |
| Q8I6Z8\|Q8I6Z8 | U4/U6.U5 tri-snRNP-associated protein 2 |
| O97303\|O97303 | U4/U6.U5 tri-snRNP-associated protein 1 |
| C0H4V5\|C0H4V5 | U4/U6.U5 small nuclear ribonucleoprotein |
| Q8I2U4\|Q8I2U4 | U4/U6 snRNA-associated-splicing factor |
| C0H5G7\|C0H5G7 | U4/U6 small nuclear ribonucleoprotein PRP4 |
| C0H4A0\|C0H4A0 | U4/U6 small nuclear ribonucleoprotein PRP31 |
| Q8IEM1\|Q8IEM1 | U4/U6 small nuclear ribonucleoprotein PRP3 |
| Q8I5F1\|Q8I5F1 | U3 small nucleolar RNA-interacting protein 2 |
| Q8IEP4\|Q8IEP4 | U3 small nucleolar RNA-associated protein 6 |
| Q8IDZ2\|Q8IDZ2 | U3 small nucleolar RNA-associated protein 4 |
| Q8IJY7\|Q8IJY7 | U3 small nucleolar RNA-associated protein 25 |
| A0A5K1K903\|A0A5K1K903 | U3 small nucleolar RNA-associated protein 21 |
| Q8IAN1\|Q8IAN1 | U3 small nucleolar RNA-associated protein 14 |
| Q8IJR4\|Q8IJR4 | U3 small nucleolar RNA-associated protein 13 |
| Q8IKZ5\|Q8IKZ5 | U3 small nucleolar RNA-associated protein 12 |
| A0A144A0U0\|A0A144A0U0 | U3 small nucleolar RNA-associated protein 11 OS |
| Q8IBM7\|Q8IBM7 | U3 small nucleolar RNA-associated protein |
| Q8IJD2\|Q8IJD2 | U3 small nucleolar ribonucleoprotein protein MPP10 |
| Q8IB23\|Q8IB23 | U3 small nucleolar ribonucleoprotein protein IMP4 |
| Q8IJ59\|Q8IJ59 | U2 snRNA/tRNA pseudouridine synthase |
| Q8I2G9\|Q8I2G9 | U2 small nuclear ribonucleoprotein B'' |
| C0H5L8\|C0H5L8 | U2 small nuclear ribonucleoprotein A' |
| Q8IKU9\|Q8IKU9 | U1 snRNP-associated protein |
| C0H5A4\|C0H5A4 | U1 small nuclear ribonucleoprotein A |
| Q8ID37\|Q8ID37 | U1 small nuclear ribonucleoprotein |
| Q8IAR7\|Q8IAR7 | Tyrosine-tRNA ligase OS |
| Q8IIJ2\|Q8IIJ2 | Tyrosine-tRNA ligase OS |
| C0H5A8\|C0H5A8 | Tyrosine recombinase OS |
| Q8IBL0\|Q8IBL0 | Type 2A phosphatase-associated protein 42 |
| O77361\|O77361 | TVN-junction protein 1 OS |
| Q8I250\|Q8I250 | Tubulin-specific chaperone A OS |
| Q8I381\|Q8I381 | Tubulin-specific chaperone |
| C6S3C6\|C6S3C6 | Tubulin-folding cofactor C OS |
| Q8IAN7\|Q8IAN7 | Tubulin gamma chain OS |
| A0A143ZWL7\|A0A143ZWL7 | Tubulin beta chain OS |
| Q8IFP3\|Q8IFP3 | Tubulin alpha chain OS |
| Q6ZLZ9\|Q6ZLZ9 | Tubulin alpha chain OS |
| Q8IKR7\|Q8IKR7 | t-SNARE coiled-coil homology domain-containing protein OS |
| Q8IDW3\|Q8IDW3 | Tryptophanyl-tRNA synthetase OS |
| A0A144A140\|A0A144A140 | Tryptophan-tRNA ligase OS |
| A0A144A1E9\|A0A144A1E9 | tRNA-intron lyase OS |
| Q8I718\|Q8I718 | tRNA-intron lyase OS |
| Q8I4W7\|Q8I4W7 | tRNA(Phe) (4-demethylwyosine(3 |
| Q8IJL6\|Q8IJL6 | tRNA(Phe) (4-demethylwyosine(3 |
| Q9U0H6\|Q9U0H6 | tRNA(Ile)-2-lysyl-cytidine synthase OS |
| Q8IBM3\|Q8IBM3 | tRNA(His) guanylyltransferase OS |
| Q8IIG2\|Q8IIG2 | tRNA nucleotidyltransferase |
| C0H4V2\|C0H4V2 | tRNA modification GTPase |
| Q8IJK4\|Q8IJK4 | tRNA methyltransferase |
| Q8IIQ2\|Q8IIQ2 | tRNA m5C-methyltransferase |
| Q8I3L6\|Q8I3L6 | tRNA 4-demethylwyosine synthase (AdoMet-dependent) OS |
| Q8IEC3\|Q8IEC3 | tRNA (Guanine(26)-N(2))-dimethyltransferase |
| Q8IEF7\|Q8IEF7 | tRNA (adenine(58)-N(1))-methyltransferase OS |
| Q8I324\|Q8I324 | tRNA (adenine(58)-N(1))-methyltransferase non-catalytic subunit TRM6 OS |
| Q8IEN6\|Q8IEN6 | Tripartite motif protein |
| A0A144A4G4\|A0A144A4G4 | Triosephosphate isomerase OS |
| Q8I420\|Q8I420 | Triose phosphate transporter OS |
| C6KTB3\|C6KTB3 | Transportin OS |
| O96181\|O96181 | Transporter |
| Q8IKC4\|Q8IKC4 | Transmembrane protein 43 |
| C6KSW7\|C6KSW7 | Transmembrane protein 234 |
| Q8IJX1\|Q8IJX1 | Transmembrane protein 14 |
| Q8IFP5\|Q8IFP5 | Transmembrane emp24 domain-containing protein |
| A0A5K1K8K6\|A0A5K1K8K6 | Transmembrane emp24 domain-containing protein |
| Q8I6T5\|Q8I6T5 | Transmembrane emp24 domain-containing protein |
| A0A143ZZU2\|A0A143ZZU2 | Transmembrane 9 superfamily member OS |
| Q8IIU7\|Q8IIU7 | Translocon component PTEX88 OS |
| Q8ILA1\|Q8ILA1 | Translocon component PTEX150 OS |
| Q8IEC8\|Q8IEC8 | Translocation protein SEC63 |
| Q8IL86\|Q8IL86 | Translocation protein SEC62 OS |
| O97314\|O97314 | Translation machinery-associated protein |
| Q8IAX2\|Q8IAX2 | Translation initiation factor SUI1 |
| Q8I4Z4\|Q8I4Z4 | Translation initiation factor SUI1 |
| Q8IB86\|Q8IB86 | Translation initiation factor IF-3 OS |
| Q8IJ64\|Q8IJ64 | Translation initiation factor IF-3 |
| C6KSR8\|C6KSR8 | Translation initiation factor IF-2 |
| Q8IBA3\|Q8IBA3 | Translation initiation factor IF-2 |
| Q8IEJ7\|Q8IEJ7 | Translation initiation factor IF-2 |
| Q8IKE7\|Q8IKE7 | Translation initiation factor IF-1 OS |
| A0A5K1K977\|A0A5K1K977 | Translation initiation factor eIF-2B subunit gamma |
| Q8I4S8\|Q8I4S8 | Translation initiation factor eIF-2B subunit beta |
| Q8IIC9\|Q8IIC9 | Translation elongation factor EF-1 |
| C6KSV3\|C6KSV3 | Transketolase OS |
| Q8IK12\|Q8IK12 | Transformer-2 protein homolog beta |
| Q8IJP9\|Q8IJP9 | Transcriptional coactivator ADA2 OS |
| C6KT72\|C6KT72 | Transcription or splicing factor-like protein |
| Q8ILH6\|Q8ILH6 | Transcription initiation TFIID-like |
| Q8I3P1\|Q8I3P1 | Transcription initiation factor TFIID subunit 10 |
| Q8I2M0\|Q8I2M0 | Transcription initiation factor TFIID subunit |
| Q8I237\|Q8I237 | Transcription initiation factor TFIIB |
| Q8IHS1\|Q8IHS1 | Transcription initiation factor IIF subunit beta |
| C0H4N3\|C0H4N3 | Transcription initiation factor IIE subunit alpha |
| Q8I4S7\|Q8I4S7 | Transcription initiation factor IIA subunit 2 |
| A0A143ZY73\|A0A143ZY73 | Transcription initiation factor IIA subunit 1 |
| Q9U0L3\|Q9U0L3 | Transcription factor |
| Q8IM32\|Q8IM32 | Transcription elongation factor SPT6 |
| C6KSV4\|C6KSV4 | Transcription elongation factor SPT5 |
| Q8IJA5\|Q8IJA5 | Transcription elongation factor SPT4 |
| Q8IBV2\|Q8IBV2 | Transcription elongation factor s-II |
| Q8I306\|Q8I306 | Transcription elongation factor 1 homolog OS |
| Q8IK89\|Q8IK89 | Trailer hitch homolog |
| O77358\|O77358 | Trafficking protein particle complex subunit OS |
| Q8I1Q2\|Q8I1Q2 | Trafficking protein particle complex subunit OS |
| Q8IM42\|Q8IM42 | Trafficking protein particle complex subunit OS |
| A0A144A213\|A0A144A213 | Trafficking protein particle complex subunit OS |
| Q8I451\|Q8I451 | Trafficking protein particle complex subunit 8 |
| C6KT27\|C6KT27 | Trafficking protein particle complex subunit 6A |
| C0H4V0\|C0H4V0 | Trafficking protein particle complex subunit 2-like protein |
| Q8IE05\|Q8IE05 | Trafficking protein particle complex subunit 2 |
| C6KTE3\|C6KTE3 | TPH domain-containing protein |
| Q8I3Z9\|Q8I3Z9 | Topoisomerase I OS |
| A0A143ZYV4\|A0A143ZYV4 | TMEM65 domain-containing protein |
| Q8I1X3\|Q8I1X3 | TMEM33 domain-containing protein |
| O96246\|O96246 | TMEM121 domain-containing protein |
| Q8I472\|Q8I472 | Tim10/DDP family zinc finger protein |
| Q8I5M8\|Q8I5M8 | Thrombospondin-related apical membrane protein OS |
| Q76NM2\|Q76NM2 | Thrombospondin-related anonymous protein OS |
| Q8IIA4\|Q8IIA4 | Threonyl-tRNA synthetase OS |
| Q8I610\|Q8I610 | Threonylcarbamoyl-AMP synthase |
| Q8I4T6\|Q8I4T6 | THO complex subunit 2 |
| Q8IDH5\|Q8IDH5 | Thioredoxin-related protein |
| Q8IBS1\|Q8IBS1 | Thioredoxin-like protein OS |
| Q8IKL4\|Q8IKL4 | Thioredoxin-like protein OS |
| C0H561\|C0H561 | Thioredoxin-like protein 2 OS |
| Q8I2W0\|Q8I2W0 | Thioredoxin-like protein 1 |
| Q8I3H6\|Q8I3H6 | Thioredoxin-like protein |
| Q8ILQ8\|Q8ILQ8 | Thioredoxin-like protein |
| Q8IIV8\|Q8IIV8 | Thioredoxin-like mero protein OS |
| Q8I5B9\|Q8I5B9 | Thioredoxin-like associated protein 1 |
| Q8IDP4\|Q8IDP4 | Thioredoxin-2 OS |
| Q8I5Q6\|Q8I5Q6 | Thioredoxin peroxidase 2 OS |
| A0A144A4E0\|A0A144A4E0 | Thioredoxin OS |
| C0H5C4\|C0H5C4 | Thioredoxin domain-containing protein OS |
| C0H556\|C0H556 | Thiamine diphosphokinase OS |
| Q8I391\|Q8I391 | TFIIS N-terminal domain-containing protein OS |
| Q8II85\|Q8II85 | TFIIS central domain-containing protein |
| C6KTE5\|C6KTE5 | Tetratricopeptide repeat protein |
| A0A143ZWF7\|A0A143ZWF7 | Tetratricopeptide repeat protein |
| Q8IIR4\|Q8IIR4 | Tetratricopeptide repeat protein |
| Q8I5Y9\|Q8I5Y9 | Tetratricopeptide repeat protein |
| Q8I5S8\|Q8I5S8 | Tetratricopeptide repeat protein |
| Q8IES8\|Q8IES8 | Tetratricopeptide repeat protein |
| Q8IEK8\|Q8IEK8 | Tetratricopeptide repeat protein |
| A0A5K1K972\|A0A5K1K972 | Tetratricopeptide repeat protein |
| Q8I568\|Q8I568 | TetQ family GTPase |
| C0H558\|C0H558 | Telomeric repeat binding factor 1 |
| Q7KQK4\|Q7KQK4 | Telomere repeat-binding zinc finger protein OS |
| C6KST5\|C6KST5 | T-complex protein 1 subunit zeta OS |
| O96220\|O96220 | T-complex protein 1 subunit theta OS |
| Q8I5C4\|Q8I5C4 | T-complex protein 1 subunit gamma OS |
| O97282\|O97282 | T-complex protein 1 subunit epsilon OS |
| C0H5I7\|C0H5I7 | T-complex protein 1 subunit delta OS |
| Q8I612\|Q8I612 | TBCC domain-containing protein |
| A0A144A0A3\|A0A144A0A3 | TBC domain-containing protein |
| Q8IDL4\|Q8IDL4 | TBC domain protein |
| Q8I226\|Q8I226 | TatD-like deoxyribonuclease OS |
| Q8I440\|Q8I440 | TATA-box-binding protein OS |
| C6KSX9\|C6KSX9 | Syntaxin-binding protein |
| Q8IKV1\|Q8IKV1 | Syntaxin-6 |
| O96189\|O96189 | Syntaxin |
| Q8IIW1\|Q8IIW1 | Syntaxin |
| Q8I5U8\|Q8I5U8 | Syntaxin |
| Q8I4Z9\|Q8I4Z9 | Syntaxin |
| C0H5E1\|C0H5E1 | Syntaxin |
| Q8IE08\|Q8IE08 | Syntaxin |
| Q8ILE4\|Q8ILE4 | Syntaxin |
| Q8I5M6\|Q8I5M6 | Symplekin |
| C0H4E7\|C0H4E7 | SWIB/MDM2 domain-containing protein OS |
| O97304\|O97304 | Survival motor neuron-like protein OS |
| Q8ILF1\|Q8ILF1 | Surface-related antigen SRA OS |
| Q8I3F8\|Q8I3F8 | SURF1 domain-containing protein |
| Q8ID38\|Q8ID38 | Suppressor of kinetochore protein 1 |
| Q8I5B4\|Q8I5B4 | Sun-family protein |
| Q8I301\|Q8I301 | SUMO-conjugating enzyme UBC9 OS |
| Q8I553\|Q8I553 | SUMO-activating enzyme subunit OS |
| Q8IHS2\|Q8IHS2 | SUMO-activating enzyme subunit 1 OS |
| Q8I242\|Q8I242 | Sulfhydryl oxidase OS |
| Q8I509\|Q8I509 | Sulfhydryl oxidase OS |
| Q8I3K0\|Q8I3K0 | Suf domain-containing protein OS |
| Q8IJU2\|Q8IJU2 | Succinate dehydrogenase subunit 4 |
| C6KSV6\|C6KSV6 | Succinate dehydrogenase subunit 3 |
| Q8IJ66\|Q8IJ66 | Succinate dehydrogenase [ubiquinone] flavoprotein subunit |
| Q8IHZ5\|SUB2 | Subtilisin-like protease 2 OS |
| Q8I0V0\|SUB1 | Subtilisin-like protease 1 OS |
| Q8I5B7\|Q8I5B7 | Subpellicular microtubule protein 2 |
| Q8I357\|Q8I357 | Subpellicular microtubule protein 1 |
| Q8I3L3\|Q8I3L3 | Structural maintenance of chromosomes protein 6 |
| Q8IIC5\|Q8IIC5 | Structural maintenance of chromosomes protein 5 OS |
| Q8I413\|Q8I413 | Structural maintenance of chromosomes protein 4 OS |
| Q8II57\|Q8II57 | Structural maintenance of chromosomes protein 1 |
| Q8IL67\|Q8IL67 | Stromal-processing peptidase |
| Q8I401\|Q8I401 | Stripes inner membrane complex protein |
| O77376\|O77376 | Stomatin-like protein |
| O97329\|O97329 | Stevor-like OS |
| Q8I2C0\|Q8I2C0 | Stevor OS |
| O96118\|O96118 | Stevor OS |
| O96283\|O96283 | Stevor OS |
| O96291\|O96291 | Stevor OS |
| Q9U5L9\|Q9U5L9 | Stevor OS |
| Q9U5M0\|Q9U5M0 | Stevor OS |
| Q8I201\|Q8I201 | Stevor OS |
| Q8IFL0\|Q8IFL0 | Stevor OS |
| C6KTF1\|C6KTF1 | Stevor OS |
| A0A143ZXI1\|A0A143ZXI1 | Stevor OS |
| Q8IBE5\|Q8IBE5 | Stevor OS |
| A0A143ZWI7\|A0A143ZWI7 | Stevor OS |
| A0A143ZXE3\|A0A143ZXE3 | Stevor OS |
| A0A143ZW11\|A0A143ZW11 | Stevor OS |
| A0A143ZXP1\|A0A143ZXP1 | Stevor OS |
| Q8I3D8\|Q8I3D8 | Stevor OS |
| Q8IJ07\|Q8IJ07 | Stevor OS |
| Q8IHM4\|Q8IHM4 | Stevor OS |
| Q8I4P6\|Q8I4P6 | Stevor OS |
| Q8I4P4\|Q8I4P4 | Stevor OS |
| Q8IEU4\|Q8IEU4 | Stevor OS |
| Q8IM81\|Q8IM81 | Stevor OS |
| A0A144A3E5\|A0A144A3E5 | Stevor OS |
| C0H4B9\|C0H4B9 | Steroid dehydrogenase |
| Q8I298\|Q8I298 | StAR-related lipid transfer protein OS |
| O96208\|O96208 | SRR1-like protein OS |
| Q8I624\|Q8I624 | SRAP domain-containing protein |
| C6S3F9\|C6S3F9 | Sporozoite surface protein essential for liver stage development |
| Q8I3U2\|Q8I3U2 | Sporozoite surface antigen MB2 OS |
| Q8IDR4\|Q8IDR4 | Sporozoite protein essential for cell traversal OS |
| Q8IBC9\|Q8IBC9 | Sporozoite invasion-associated protein 2 OS |
| Q8IBD1\|Q8IBD1 | Sporozoite and liver stage tryptophan-rich protein |
| Q8IIH4\|Q8IIH4 | Splicing factor U2AF small subunit |
| Q8IKE9\|Q8IKE9 | Splicing factor U2AF large subunit |
| C0H5I5\|C0H5I5 | Splicing factor subunit OS |
| Q8I5G8\|Q8I5G8 | Splicing factor 3B subunit 6 |
| Q8ILQ0\|Q8ILQ0 | Splicing factor 3B subunit 4 |
| Q8I574\|Q8I574 | Splicing factor 3B subunit 3 |
| Q8IKL7\|Q8IKL7 | Splicing factor 3B subunit 2 |
| O77327\|O77327 | Splicing factor 3B subunit 1 |
| Q8I2R0\|Q8I2R0 | Splicing factor 3A subunit 3 |
| C6KT39\|C6KT39 | Splicing factor 3A subunit 2 |
| Q8IK93\|Q8IK93 | Splicing factor 3A subunit 1 |
| Q8IE99\|Q8IE99 | Splicing factor 1 OS |
| O97225\|O97225 | Spindle pole body protein |
| A0A143ZVB5\|A0A143ZVB5 | Sphingomyelin synthase 1 |
| Q8II73\|Q8II73 | Spermidine synthase OS |
| A0A143ZW95\|A0A143ZW95 | Sorting assembly machinery 50 kDa subunit |
| Q8IKV8\|Q8IKV8 | Sortilin OS |
| Q8IKM4\|Q8IKM4 | snRNA-activating protein complex subunit 3 |
| C6KT82\|C6KT82 | SNF2 helicase |
| C6S3K5\|C6S3K5 | SND2 domain-containing protein |
| Q8I346\|Q8I346 | SNARE protein OS |
| Q8I3G6\|Q8I3G6 | SNARE protein |
| Q8IB99\|Q8IB99 | SNARE protein |
| Q8IET3\|Q8IET3 | SNARE protein |
| C0H5C6\|C0H5C6 | SNARE protein |
| C0H5D3\|C0H5D3 | SNARE protein |
| Q8IKY7\|Q8IKY7 | SNARE protein |
| Q8ID52\|Q8ID52 | SNARE associated Golgi protein |
| Q8I444\|Q8I444 | Small ubiquitin-related modifier OS |
| Q8I5G2\|Q8I5G2 | Small subunit rRNA processing factor |
| Q8I5X4\|Q8I5X4 | Small subunit rRNA processing factor |
| Q8I275\|Q8I275 | Small ribosomal subunit assembling AARP2 protein OS |
| Q8ILU8\|Q8ILU8 | Small nuclear ribonucleoprotein-associated protein B |
| C0H529\|C0H529 | Small nuclear ribonucleoprotein Sm D3 OS |
| O96265\|O96265 | Small nuclear ribonucleoprotein Sm D2 OS |
| Q8IIA8\|Q8IIA8 | Small nuclear ribonucleoprotein Sm D1 OS |
| Q8IB57\|Q8IB57 | Small nuclear ribonucleoprotein G OS |
| Q8IDJ7\|Q8IDJ7 | Small nuclear ribonucleoprotein E OS |
| Q8IB02\|Q8IB02 | Small heat shock protein HSP20 |
| Q8I1S0\|Q8I1S0 | Small GTP-binding protein sar1 OS |
| Q8IC43\|Q8IC43 | Small exported membrane protein 1 OS |
| Q8II94\|Q8II94 | Sm protein F OS |
| C0H597\|C0H597 | Sld5 domain-containing protein OS |
| Q8IEQ9\|Q8IEQ9 | Site-2 protease S2P |
| Q8I415\|Q8I415 | Single-stranded DNA-binding protein OS |
| Q8IJX3\|Q8IJX3 | Single-strand telomeric DNA-binding protein GBP2 |
| C0H4Q0\|C0H4Q0 | Signal recognition particle subunit SRP9 OS |
| C6KT56\|C6KT56 | Signal recognition particle subunit SRP68 |
| Q8I5P4\|Q8I5P4 | Signal recognition particle subunit SRP19 OS |
| Q8I613\|Q8I613 | Signal recognition particle subunit SRP14 OS |
| Q8II00\|Q8II00 | Signal recognition particle subunit SRP |
| Q8I4W4\|Q8I4W4 | Signal recognition particle receptor subunit beta OS |
| Q8ID42\|Q8ID42 | Signal recognition particle receptor subunit alpha |
| Q8IKX4\|Q8IKX4 | Signal recognition particle 54 kDa protein OS |
| Q8IKQ9\|Q8IKQ9 | Signal peptide peptidase OS |
| Q8IE14\|Q8IE14 | Signal peptidase I OS |
| Q8ILC7\|Q8ILC7 | Signal peptidase complex subunit SPC1 |
| Q8I3A5\|Q8I3A5 | Signal peptidase complex subunit 3 |
| Q9NFA0\|Q9NFA0 | Signal peptidase complex subunit 2 OS |
| Q8IKE5\|Q8IKE5 | Shewanella-like protein phosphatase 1 |
| Q6ZMA7\|Q6ZMA7 | Sexual stage-specific protein OS |
| Q8IER9\|Q8IER9 | SET domain-containing protein OS |
| Q8I422\|Q8I422 | SET domain protein |
| Q8IBB0\|Q8IBB0 | SET domain protein |
| Q8IBS3\|Q8IBS3 | Seryl-tRNA synthetase OS |
| A0A143ZZQ0\|A0A143ZZQ0 | Seryl-tRNA synthetase OS |
| Q9TY96\|SERA6 | Serine-repeat antigen protein 6 OS |
| O97259\|O97259 | Serine/threonine-protein phosphatase OS |
| Q8IAM8\|Q8IAM8 | Serine/threonine-protein phosphatase OS |
| Q8I2Q4\|Q8I2Q4 | Serine/threonine-protein phosphatase OS |
| Q8I2N2\|Q8I2N2 | Serine/threonine-protein phosphatase OS |
| C6S3C9\|C6S3C9 | Serine/threonine-protein phosphatase OS |
| Q8ILV1\|Q8ILV1 | Serine/threonine-protein phosphatase OS |
| Q8ILL9\|Q8ILL9 | Serine/threonine-protein phosphatase OS |
| Q8IKH5\|Q8IKH5 | Serine/threonine-protein phosphatase OS |
| Q8ILG3\|Q8ILG3 | Serine/threonine-protein phosphatase 2A activator OS |
| Q8IKJ1\|Q8IKJ1 | Serine/threonine protein phosphatase UIS2 |
| Q8IM55\|Q8IM55 | Serine/threonine protein phosphatase CPPED1 |
| Q8IB64\|Q8IB64 | Serine/threonine protein kinase VPS15 |
| Q8IEG4\|Q8IEG4 | Serine/threonine protein kinase PK9 OS |
| C0H466\|C0H466 | Serine/threonine protein kinase |
| C0H515\|C0H515 | Serine/threonine protein kinase |
| Q8IIL5\|Q8IIL5 | Serine/threonine protein kinase |
| Q8IHP1\|Q8IHP1 | Serine/threonine protein kinase |
| A0A5K1K8V9\|A0A5K1K8V9 | Serine/threonine protein kinase |
| Q8IKX5\|Q8IKX5 | Serine/threonine protein kinase |
| O96226\|O96226 | Serine/threonine protein kinase |
| Q8IHR5\|Q8IHR5 | Serine/threonine protein kinase |
| Q8I468\|Q8I468 | Serine/arginine-rich splicing factor 12 OS |
| Q8I3T5\|Q8I3T5 | Serine/arginine-rich splicing factor 1 OS |
| Q8ILT9\|Q8ILT9 | Serine palmitoyltransferase |
| Q8I566\|GLYA | Serine hydroxymethyltransferase OS |
| C0H4T3\|C0H4T3 | Separase OS |
| C0H4Y4\|C0H4Y4 | Sentrin-specific protease 2 |
| Q8I583\|Q8I583 | Sentrin-specific protease 1 OS |
| Q9TY98\|Q9TY98 | Sel1 repeat-containing protein |
| O96183\|O96183 | Secretory complex protein 61 gamma subunit OS |
| O96207\|O96207 | Secreted protein with altered thrombospondin repeat domain OS |
| C6KT40\|C6KT40 | Secreted ookinete protein 25 |
| A0A5K1K8W5\|A0A5K1K8W5 | Secreted ookinete protein |
| C0H4E9\|C0H4E9 | Secreted ookinete protein |
| C6KTD7\|C6KTD7 | Secreted ookinete protein |
| Q8IM51\|Q8IM51 | Secreted ookinete adhesive protein |
| Q8IJ69\|Q8IJ69 | Sec1 family protein |
| C6KSN0\|C6KSN0 | Sde2 |
| Q8IKR4\|Q8IKR4 | SCD domain-containing protein OS |
| Q8IB87\|Q8IB87 | SAYSvFN domain-containing protein |
| A0A5K1K911\|A0A5K1K911 | Sas10 domain-containing protein |
| C0H535\|C0H535 | SAP domain-containing protein |
| A0A143ZZC7\|A0A143ZZC7 | S-antigen protein OS |
| Q8I556\|Q8I556 | S-adenosyl-methyltransferase |
| Q8IKX0\|Q8IKX0 | S-adenosylmethionine-dependent methyltransferase |
| Q7K6A4\|Q7K6A4 | S-adenosylmethionine synthase OS |
| Q8IJ77\|Q8IJ77 | S-adenosylmethionine decarboxylase/ornithine decarboxylase OS |
| Q8IL76\|Q8IL76 | Sad1/UNC domain-containing protein |
| C6KSM6\|C6KSM6 | SAC3 domain-containing protein |
| Q8IIU3\|Q8IIU3 | RuvB-like helicase OS |
| Q8ID85\|Q8ID85 | RuvB-like helicase OS |
| O96219\|O96219 | RTR1 domain-containing protein |
| Q8I4T4\|Q8I4T4 | rRNA-processing protein FCF2 |
| C0H4V7\|C0H4V7 | rRNA-processing protein FCF1 |
| Q8I5G3\|Q8I5G3 | rRNA-processing protein |
| Q8IM49\|Q8IM49 | rRNA biogenesis protein RRP5 |
| Q8I247\|Q8I247 | rRNA biogenesis protein RRP36 |
| A0A144A0V8\|A0A144A0V8 | rRNA adenine N(6)-methyltransferase OS |
| Q8ILT8\|Q8ILT8 | rRNA adenine N(6)-methyltransferase OS |
| Q8IM23\|Q8IM23 | rRNA 2'-O-methyltransferase fibrillarin |
| Q8II69\|Q8II69 | rRNA (Cytosine-C(5))-methyltransferase |
| A0A143ZZZ9\|A0A143ZZZ9 | RNA-splicing ligase RtcB homolog OS |
| Q8I1Q0\|Q8I1Q0 | RNA-binding protein NOB1 |
| Q8IJK1\|Q8IJK1 | RNA-binding protein 34 |
| O77316\|O77316 | RNA-binding protein |
| O97318\|O97318 | RNA-binding protein |
| C6KSN1\|C6KSN1 | RNA-binding protein |
| C6KSP9\|C6KSP9 | RNA-binding protein |
| C6KTD1\|C6KTD1 | RNA-binding protein |
| Q8IBU0\|Q8IBU0 | RNA-binding protein |
| C0H4P9\|C0H4P9 | RNA-binding protein |
| C0H4U1\|C0H4U1 | RNA-binding protein |
| C0H4W4\|C0H4W4 | RNA-binding protein |
| Q8IB66\|Q8IB66 | RNA-binding protein |
| Q8I2N3\|Q8I2N3 | RNA-binding protein |
| C0H570\|C0H570 | RNA-binding protein |
| Q8IIQ7\|Q8IIQ7 | RNA-binding protein |
| Q8II95\|Q8II95 | RNA-binding protein |
| Q8I5X0\|Q8I5X0 | RNA-binding protein |
| Q8I4Y7\|Q8I4Y7 | RNA-binding protein |
| Q8IEK9\|Q8IEK9 | RNA-binding protein |
| C0H5C7\|C0H5C7 | RNA-binding protein |
| A0A5K1K940\|A0A5K1K940 | RNA-binding protein |
| Q8IE18\|Q8IE18 | RNA-binding protein |
| C0H5J5\|C0H5J5 | RNA-binding protein |
| Q8IKT8\|Q8IKT8 | RNA-binding protein |
| Q8IJG3\|Q8IJG3 | RNA-binding protein |
| Q8IL17\|Q8IL17 | RNA-binding protein |
| Q8I5D9\|Q8I5D9 | RNA pseudouridylate synthase |
| Q8I5D3\|Q8I5D3 | RNA pseudouridylate synthase |
| Q8IJ79\|Q8IJ79 | RNA polymerase II-associated protein 1 |
| Q8IL51\|Q8IL51 | RNA polymerase II transcription factor B subunit 5 |
| Q8IDG5\|Q8IDG5 | RNA polymerase II transcription factor B subunit 4 |
| Q8IHR1\|Q8IHR1 | RNA methyltransferase OS |
| A0A5K1K9E4\|A0A5K1K9E4 | RNA methyltransferase |
| Q8IJ98\|Q8IJ98 | RNA methyltransferase |
| Q8ILD7\|Q8ILD7 | RNA methyltransferase |
| A0A5K1K8J0\|A0A5K1K8J0 | RNA lariat debranching enzyme |
| Q8I457\|Q8I457 | RNA helicase OS |
| C6KT73\|C6KT73 | RNA helicase OS |
| C0H4K7\|C0H4K7 | RNA helicase |
| C6KSZ8\|C6KSZ8 | RNA and export factor binding protein |
| Q8I717\|Q8I717 | RNA 3'-terminal phosphate cyclase-like protein |
| Q8II27\|Q8II27 | RNA (Uracil-5-)methyltransferase |
| C0H5H2\|C0H5H2 | RING-type domain-containing protein OS |
| Q8I2F6\|Q8I2F6 | Ring-exported protein 4 OS |
| Q8I2G0\|Q8I2G0 | Ring-exported protein 2 OS |
| Q8I2G1\|Q8I2G1 | Ring-exported protein 1 OS |
| O96182\|O96182 | RING zinc finger protein |
| Q8I660\|Q8I660 | RING zinc finger protein |
| Q8IJW9\|Q8IJW9 | RING zinc finger protein |
| Q8IJC2\|Q8IJC2 | RING zinc finger protein |
| Q8IDY8\|Q8IDY8 | RING zinc finger protein |
| Q8IM37\|Q8IM37 | RING zinc finger protein |
| Q8IIW3\|Q8IIW3 | RIIa domain-containing protein OS |
| Q9NFB5\|Q9NFB5 | Rifin |
| Q9NFB3\|Q9NFB3 | Rifin |
| Q9NFB2\|Q9NFB2 | Rifin |
| Q9NFB0\|Q9NFB0 | Rifin |
| B9ZSH6\|B9ZSH6 | Rifin |
| Q8I2C1\|Q8I2C1 | Rifin |
| Q8I2B9\|Q8I2B9 | Rifin |
| Q8I2D4\|Q8I2D4 | Rifin |
| Q8I2C8\|Q8I2C8 | Rifin |
| Q8I2C7\|Q8I2C7 | Rifin |
| Q8I2C5\|Q8I2C5 | Rifin |
| O96109\|O96109 | Rifin |
| O96112\|O96112 | Rifin |
| O96113\|O96113 | Rifin |
| O96114\|O96114 | Rifin |
| O96116\|O96116 | Rifin |
| O96288\|O96288 | Rifin |
| O96289\|O96289 | Rifin |
| O96290\|O96290 | Rifin |
| O96292\|O96292 | Rifin |
| O96293\|O96293 | Rifin |
| O96295\|O96295 | Rifin |
| O97325\|O97325 | Rifin |
| O97327\|O97327 | Rifin |
| O97310\|O97310 | Rifin |
| Q8I219\|Q8I219 | Rifin |
| Q8I216\|Q8I216 | Rifin |
| Q8I214\|Q8I214 | Rifin |
| Q8I213\|Q8I213 | Rifin |
| Q8I212\|Q8I212 | Rifin |
| Q8I211\|Q8I211 | Rifin |
| Q8I210\|Q8I210 | Rifin |
| Q8I209\|Q8I209 | Rifin |
| Q8I1V5\|Q8I1V5 | Rifin |
| Q8I1V4\|Q8I1V4 | Rifin |
| Q8IFQ3\|Q8IFQ3 | Rifin |
| Q8IFK9\|Q8IFK9 | Rifin |
| Q8IFK8\|Q8IFK8 | Rifin |
| Q8I494\|Q8I494 | Rifin |
| Q8I493\|Q8I493 | Rifin |
| Q8I3E7\|Q8I3E7 | Rifin |
| C6KSK9\|C6KSK9 | Rifin |
| C6KSL1\|C6KSL1 | Rifin |
| C6KT17\|C6KT17 | Rifin |
| C6KTF0\|C6KTF0 | Rifin |
| C6KTF2\|C6KTF2 | Rifin |
| C6KTF4\|C6KTF4 | Rifin |
| C6KTF5\|C6KTF5 | Rifin |
| C6KTF6\|C6KTF6 | Rifin |
| A0A143ZVI0\|A0A143ZVI0 | Rifin |
| A0A143ZWN4\|A0A143ZWN4 | Rifin |
| Q8IC49\|Q8IC49 | Rifin |
| Q8IBW6\|Q8IBW6 | Rifin |
| Q8IBE3\|Q8IBE3 | Rifin |
| Q8IBE2\|Q8IBE2 | Rifin |
| C0H4Q5\|C0H4Q5 | Rifin |
| Q8IBD9\|Q8IBD9 | Rifin |
| A0A143ZVK7\|A0A143ZVK7 | Rifin |
| Q8IAK4\|Q8IAK4 | Rifin |
| Q8IAS6\|Q8IAS6 | Rifin |
| A0A143ZYB5\|A0A143ZYB5 | Rifin |
| A0A143ZVN0\|A0A143ZVN0 | Rifin |
| A0A143ZVN3\|A0A143ZVN3 | Rifin |
| A0A143ZWJ4\|A0A143ZWJ4 | Rifin |
| A0A143ZWV2\|A0A143ZWV2 | Rifin |
| Q8I3E4\|Q8I3E4 | Rifin |
| Q8I3E3\|Q8I3E3 | Rifin |
| Q8I3E2\|Q8I3E2 | Rifin |
| Q8I3E1\|Q8I3E1 | Rifin |
| Q8I3E0\|Q8I3E0 | Rifin |
| Q8I3D9\|Q8I3D9 | Rifin |
| Q8I3D7\|Q8I3D7 | Rifin |
| Q8I3D4\|Q8I3D4 | Rifin |
| Q8I2E7\|Q8I2E7 | Rifin |
| Q8I2E6\|Q8I2E6 | Rifin |
| Q8I2E5\|Q8I2E5 | Rifin |
| Q8I082\|Q8I082 | Rifin |
| Q8IK36\|Q8IK36 | Rifin |
| Q8IK35\|Q8IK35 | Rifin |
| Q8IK34\|Q8IK34 | Rifin |
| Q8IK32\|Q8IK32 | Rifin |
| Q8IJ08\|Q8IJ08 | Rifin |
| Q8IJ06\|Q8IJ06 | Rifin |
| Q8IJ05\|Q8IJ05 | Rifin |
| A0A143ZXG5\|A0A143ZXG5 | Rifin |
| Q8IJ02\|Q8IJ02 | Rifin |
| Q8IJ00\|Q8IJ00 | Rifin |
| Q8IIZ8\|Q8IIZ8 | Rifin |
| Q8IIZ7\|Q8IIZ7 | Rifin |
| Q8IIZ3\|Q8IIZ3 | Rifin |
| Q8IIZ1\|Q8IIZ1 | Rifin |
| Q8IIY5\|Q8IIY5 | Rifin |
| Q8IIY4\|Q8IIY4 | Rifin |
| Q8IIY3\|Q8IIY3 | Rifin |
| Q8IHM3\|Q8IHM3 | Rifin |
| Q8IHM2\|Q8IHM2 | Rifin |
| Q8IHM1\|Q8IHM1 | Rifin |
| Q8I642\|Q8I642 | Rifin |
| Q8I641\|Q8I641 | Rifin |
| Q8I0E0\|Q8I0E0 | Rifin |
| Q8I4Q0\|Q8I4Q0 | Rifin |
| Q8I4P7\|Q8I4P7 | Rifin |
| Q8I4P5\|Q8I4P5 | Rifin |
| Q8I4P3\|Q8I4P3 | Rifin |
| Q8I4P2\|Q8I4P2 | Rifin |
| Q8I4N9\|Q8I4N9 | Rifin |
| Q8I4N7\|Q8I4N7 | Rifin |
| Q8I4N6\|Q8I4N6 | Rifin |
| Q8IEV0\|Q8IEV0 | Rifin |
| Q8IEU7\|Q8IEU7 | Rifin |
| Q8IEU6\|Q8IEU6 | Rifin |
| Q8IEU5\|Q8IEU5 | Rifin |
| C0H5N2\|C0H5N2 | Rifin |
| C0H5N3\|C0H5N3 | Rifin |
| C0H5N5\|C0H5N5 | Rifin |
| C0H5N6\|C0H5N6 | Rifin |
| C0H5N8\|C0H5N8 | Rifin |
| C0H5N9\|C0H5N9 | Rifin |
| Q8IM85\|Q8IM85 | Rifin |
| Q8I0G7\|Q8I0G7 | Rifin |
| Q8IM84\|Q8IM84 | Rifin |
| Q8IK44\|Q8IK44 | Rifin |
| Q8IK40\|Q8IK40 | Rifin |
| Q8IK38\|Q8IK38 | Rifin |
| Q8I5L3\|Q8I5L3 | Ribulose-phosphate 3-epimerase OS |
| O96173\|O96173 | Ribosome-recycling factor OS |
| Q8IFQ7\|Q8IFQ7 | Ribosome-recycling factor OS |
| A0A5K1K870\|A0A5K1K870 | Ribosome-interacting GTPase 1 |
| Q8IK05\|Q8IK05 | Ribosome-binding factor A |
| Q8I516\|Q8I516 | Ribosome-binding factor A |
| Q8IJC0\|Q8IJC0 | Ribosome production factor 2 homolog OS |
| C0H549\|C0H549 | Ribosome production factor 1 |
| Q8ILY6\|Q8ILY6 | Ribosome maturation protein SBDS |
| Q8IJ86\|Q8IJ86 | Ribosome maturation factor RimM |
| Q8IIB5\|Q8IIB5 | Ribosome biogenesis regulatory protein OS |
| C6KTE0\|C6KTE0 | Ribosome biogenesis protein YTM1 |
| Q8IKV7\|Q8IKV7 | Ribosome biogenesis protein TSR1 |
| Q8ID32\|Q8ID32 | Ribosome biogenesis protein MRT4 |
| Q8IBG5\|Q8IBG5 | Ribosome biogenesis protein BRX1 |
| Q8IM36\|Q8IM36 | Ribosome biogenesis protein BOP1 homolog OS |
| Q9U0I1\|Q9U0I1 | Ribosome biogenesis GTPase A |
| Q8I655\|Q8I655 | Ribosome associated membrane protein RAMP4 |
| Q8IAZ7\|Q8IAZ7 | Ribosome assembly protein RRB1 |
| Q8IHQ8\|Q8IHQ8 | Ribosome assembly protein 4 |
| Q8IJJ7\|Q8IJJ7 | Ribosomal silencing factor RsfS |
| Q8I2Q6\|Q8I2Q6 | Ribosomal RNA-processing protein 8 OS |
| Q8IB49\|Q8IB49 | Ribosomal RNA small subunit methyltransferase NEP1 |
| Q8IEL9\|Q8IEL9 | Ribosomal RNA methyltransferase |
| Q8IC10\|Q8IC10 | Ribosomal protein S8e |
| Q8IM64\|Q8IM64 | Ribosomal protein S2 |
| Q8I3F6\|Q8I3F6 | Ribosomal protein S16 |
| Q8ID56\|Q8ID56 | Ribosomal protein S1 |
| Q8IJU5\|Q8IJU5 | Ribosomal protein L43 |
| Q8IB54\|Q8IB54 | Ribosomal protein L4 |
| C0H4L5\|C0H4L5 | Ribosomal protein L3 |
| Q8I1W3\|Q8I1W3 | Ribosomal protein L25 |
| C0H4Y5\|C0H4Y5 | Ribosomal protein L21 |
| Q8IJ68\|Q8IJ68 | Ribosomal protein L2 |
| Q8IM50\|Q8IM50 | Ribosomal protein L16 |
| C0H4A6\|C0H4A6 | Ribosomal protein L15 OS |
| O96222\|O96222 | Ribosomal protein L13 |
| O96202\|O96202 | Ribosomal protein L12 |
| Q8I3P7\|Q8I3P7 | Ribosomal large subunit pseudouridylate synthase |
| Q8I3W2\|RPIA | Ribose-5-phosphate isomerase OS |
| Q8IIC4\|Q8IIC4 | Ribonucloprotein OS |
| Q8IJN8\|Q8IJN8 | Ribonucleoside-diphosphate reductase small chain |
| Q8IM38\|Q8IM38 | Ribonucleoside-diphosphate reductase small chain |
| Q8IL94\|Q8IL94 | Ribonucleoside-diphosphate reductase OS |
| C0H4M3\|C0H4M3 | Ribonucleases P/MRP protein subunit POP1 |
| Q8IKI5\|Q8IKI5 | Ribonuclease Z |
| C6KT53\|C6KT53 | Ribonuclease P/MRP protein subunit RPP1 |
| Q8IE34\|Q8IE34 | Ribonuclease P protein subunit RPR2 |
| C6KT75\|C6KT75 | Ribonuclease OS |
| Q8I300\|Q8I300 | Ribonuclease H2 subunit C |
| Q8ILB3\|Q8ILB3 | Ribonuclease H2 subunit B |
| C6KSZ5\|C6KSZ5 | Ribonuclease |
| Q8IJV3\|Q8IJV3 | Ribonuclease |
| Q8IDB3\|Q8IDB3 | Riboflavin kinase OS |
| Q8I484\|Q8I484 | Rhoptry-associated protein 2 OS |
| C0H4M0\|C0H4M0 | Rhoptry-associated membrane antigen OS |
| Q8IBN1\|Q8IBN1 | Rhoptry-associated leucine zipper-like protein 1 OS |
| A0A5K1K9G4\|A0A5K1K9G4 | Rhoptry protein RHOP148 OS |
| Q8IKN2\|Q8IKN2 | Rhoptry protein |
| O96229\|O96229 | Rhoptry neck protein 6 OS |
| C0H4V4\|C0H4V4 | Rhoptry neck protein 5 OS |
| Q8IIK5\|Q8IIK5 | Rhoptry neck protein 4 OS |
| Q8I4R5\|Q8I4R5 | Rhoptry neck protein 3 OS |
| Q8IKV6\|Q8IKV6 | Rhoptry neck protein 2 OS |
| Q8IJM8\|Q8IJM8 | Rhoptry neck protein 12 OS |
| Q8IJS3\|Q8IJS3 | Rhoptry associated adhesin OS |
| Q8I433\|Q8I433 | RHOMBOID-like protein OS |
| A0A5K1K8S0\|A0A5K1K8S0 | RHOMBOID-like protein OS |
| Q8IIM2\|Q8IIM2 | RHOMBOID-like protein OS |
| Q8ILY3\|Q8ILY3 | RHOMBOID-like protein OS |
| Q8I3V7\|Q8I3V7 | Rhomboid protease ROM9 OS |
| A0A5K1K8X1\|A0A5K1K8X1 | Rhomboid protease ROM6 |
| Q8IJX0\|Q8IJX0 | Rho GTPase-activating protein |
| O97302\|RH5IP | Rh5-interacting protein OS |
| Q8IFM5\|RH5 | Reticulocyte-binding protein homolog 5 OS |
| C0H496\|C0H496 | Reticulocyte binding protein homolog 4 OS |
| Q8I1S2\|Q8I1S2 | Repressor of RNA polymerase III transcription MAF1 OS |
| Q9U0J0\|Q9U0J0 | Replication protein A1 |
| Q8I3A1\|Q8I3A1 | Replication protein A1 |
| Q8IIQ1\|Q8IIQ1 | Replication factor C subunit 5 |
| Q8I512\|Q8I512 | Replication factor C subunit 4 |
| Q8IKK4\|Q8IKK4 | Replication factor C subunit 3 |
| O96260\|O96260 | Replication factor C subunit 2 |
| O96271\|O96271 | Replication factor C subunit 1 OS |
| C6S3I6\|C6S3I6 | Replication factor A protein 3 |
| C0H485\|C0H485 | Regulator of initiation factor 2 (eIF2) OS |
| Q8I1Z9\|Q8I1Z9 | Regulator of chromosome condensation |
| C0H4M6\|C0H4M6 | Regulator of chromosome condensation |
| Q8IDD6\|Q8IDD6 | Regulator of chromosome condensation |
| Q8IEP7\|Q8IEP7 | RED-like protein |
| Q8IBA0\|Q8IBA0 | Receptor for activated c kinase OS |
| Q8IDG4\|Q8IDG4 | Reactive oxygen species modulator 1 |
| Q8IHR8\|Q8IHR8 | Ras-related protein Rab-6 OS |
| Q8I274\|Q8I274 | Ras-related protein Rab-5C OS |
| Q76NM7\|Q76NM7 | Ras-related protein Rab-5B OS |
| O96193\|O96193 | Ras-related protein Rab-5A OS |
| Q8I5A9\|Q8I5A9 | Ras-related protein Rab-2 OS |
| A0A5K1K8H7\|A0A5K1K8H7 | Ras-related protein Rab-1B OS |
| Q8I3W9\|Q8I3W9 | Ras-related protein Rab-1A OS |
| Q7K6B0\|Q7K6B0 | Ras-related protein Rab-18 OS |
| C0H5G2\|C0H5G2 | Ras-related protein Rab-11B OS |
| Q76NM4\|Q76NM4 | Ras-related protein Rab-11A OS |
| C0H516\|C0H516 | Ras-related protein RAB |
| O77338\|O77338 | Ras-like G protein OS |
| C6KT08\|C6KT08 | Ras GTPAse |
| Q8I3U8\|Q8I3U8 | RAP protein |
| Q8I3K5\|Q8I3K5 | RAP protein |
| C6KSP6\|C6KSP6 | RAP protein |
| C6KTC4\|C6KTC4 | RAP protein |
| Q8IC11\|Q8IC11 | RAP protein |
| Q8IJF8\|Q8IJF8 | RAP protein |
| Q8IIL8\|Q8IIL8 | RAP protein |
| Q8IIC7\|Q8IIC7 | RAP protein |
| A0A5K1K936\|A0A5K1K936 | RAP protein |
| A0A144A2U7\|A0A144A2U7 | RAP protein |
| Q8IKC6\|Q8IKC6 | RAP protein |
| C6KT54\|C6KT54 | RAP domain-containing protein OS |
| Q8I2U0\|Q8I2U0 | RAP domain-containing protein OS |
| Q8IDW7\|Q8IDW7 | RanBPM and CLTH-like protein |
| Q8I1Y6\|Q8I1Y6 | RanBD1 domain-containing protein OS |
| Q7KQK1\|Q7KQK1 | Raf kinase inhibitor OS |
| Q8IM25\|Q8IM25 | Radical SAM protein |
| Q8I5P9\|Q8I5P9 | Radial spoke head protein 9 |
| Q8I1P4\|Q8I1P4 | Rad51 domain-containing protein OS |
| Q8IL69\|Q8IL69 | Rad21 |
| Q8IJN4\|Q8IJN4 | Rab3 GTPase-activating protein non-catalytic subunit |
| C0H4P1\|C0H4P1 | Rab GTPase activator and protein kinase |
| Q8I501\|Q8I501 | Rab GDP dissociation inhibitor OS |
| Q8I507\|Q8I507 | Queuine tRNA-ribosyltransferase |
| C6KTA4\|C6KTA4 | Pyruvate kinase OS |
| Q8IJ37\|Q8IJ37 | Pyruvate kinase OS |
| Q8IIB8\|Q8IIB8 | Pyruvate dehydrogenase E1 component subunit alpha OS |
| Q8IDC6\|Q8IDC6 | Pyrroline-5-carboxylate reductase |
| C6KT01\|C6KT01 | Pyridoxal kinase OS |
| Q8I4W5\|Q8I4W5 | Pyrid |
| Q8I365\|Q8I365 | Putative tRNA (cytidine(32)/guanosine(34)-2'-O)-methyltransferase OS |
| Q8IDF8\|Q8IDF8 | Putative rRNA methyltransferase OS |
| Q8I3X4\|Q8I3X4 | Purine nucleoside phosphorylase OS |
| Q8IE48\|Q8IE48 | PUB domain-containing protein |
| Q8I5I3\|Q8I5I3 | P-type phospholipid transporter OS |
| O96199\|O96199 | Pseudouridine synthase |
| O77325\|O77325 | PRP19/PSO4 homolog OS |
| Q8IKU3\|Q8IKU3 | Proton-translocating NAD(P)(+) transhydrogenase OS |
| Q8ILY9\|Q8ILY9 | Protein-synthesizing GTPase OS |
| Q8IJR8\|Q8IJR8 | Protein-serine/threonine phosphatase OS |
| Q8IDE5\|Q8IDE5 | Protein-serine/threonine phosphatase OS |
| O77388\|O77388 | Protein YOP1 |
| C6S3G6\|C6S3G6 | Protein YIPF OS |
| Q8IKB7\|Q8IKB7 | Protein YIPF OS |
| Q8IIN1\|Q8IIN1 | Protein tyrosine phosphatase OS |
| Q8I5J7\|Q8I5J7 | Protein TSSC1 |
| Q8IB40\|Q8IB40 | Protein transport protein YIF1 |
| Q8IBY2\|Q8IBY2 | Protein transport protein USE1 |
| C0H4W6\|C0H4W6 | Protein transport protein Sec61 subunit beta OS |
| Q8IDN6\|Q8IDN6 | Protein transport protein SEC61 subunit alpha OS |
| O96221\|O96221 | Protein transport protein SEC31 OS |
| C0H489\|C0H489 | Protein transport protein Sec24B |
| C0H5J6\|C0H5J6 | Protein transport protein Sec24A OS |
| Q8IB60\|Q8IB60 | Protein transport protein SEC23 OS |
| O97323\|O97323 | Protein transport protein SEC22 OS |
| A0A5K1K9E7\|A0A5K1K9E7 | Protein transport protein SEC20 |
| Q8I5B3\|Q8I5B3 | Protein transport protein SEC13 OS |
| Q8IL42\|Q8IL42 | Protein transport protein SEC |
| Q8I1P5\|Q8I1P5 | Protein transport protein GOT1 |
| Q8IIP9\|Q8IIP9 | Protein transport protein BOS1 |
| Q8I576\|Q8I576 | Protein TOC |
| Q8I1Q6\|Q8I1Q6 | Protein SOC1 |
| O96212\|O96212 | Protein SIS1 OS |
| A0A144A1B3\|A0A144A1B3 | Protein SEY1 homolog OS |
| Q8IL19\|Q8IL19 | Protein serine/threonine kinase-1 OS |
| Q8IBT9\|Q8IBT9 | Protein SDA1 |
| Q8IC00\|Q8IC00 | Protein SCO1 |
| Q8I3B7\|Q8I3B7 | Protein RER1 OS |
| C0H4F2\|C0H4F2 | Protein phosphatase PPM9 |
| Q8II13\|Q8II13 | Protein phosphatase PPM8 |
| Q8IEM2\|Q8IEM2 | Protein phosphatase PPM6 |
| Q8IHY0\|Q8IHY0 | Protein phosphatase PPM2 OS |
| Q8IEB9\|Q8IEB9 | Protein phosphatase PP2A regulatory subunit A |
| Q8I487\|Q8I487 | Protein phosphatase OS |
| A0A143ZY63\|A0A143ZY63 | Protein phosphatase OS |
| Q8IJ88\|Q8IJ88 | Protein phosphatase inhibitor 3 OS |
| C0H480\|C0H480 | Protein phosphatase inhibitor 2 OS |
| C6S3E1\|C6S3E1 | Protein phosphatase 1 regulatory subunit 22 OS |
| Q8IKQ0\|Q8IKQ0 | Protein PET11 |
| Q8I478\|Q8I478 | Protein Mpv1 |
| Q8IKD8\|Q8IKD8 | Protein MGET OS |
| C0H584\|C0H584 | Protein MAM3 |
| A0A143ZWE8\|A0A143ZWE8 | Protein MAK16 |
| Q8IB88\|Q8IB88 | Protein KRI1 |
| C0H590\|C0H590 | Protein kish OS |
| C6KTB8\|C6KTB8 | Protein kinase PK4 OS |
| Q8IIP2\|Q8IIP2 | Protein kinase domain-containing protein OS |
| Q8IDW1\|Q8IDW1 | Protein kinase 6 OS |
| Q8I534\|Q8I534 | Protein kinase 2 OS |
| Q7YTF7\|Q7YTF7 | Protein kinase |
| C0H482\|C0H482 | Protein kinase |
| C0H563\|C0H563 | Protein kinase |
| C0H5B8\|C0H5B8 | Protein kinase |
| Q8I2P8\|Q8I2P8 | Protein kinase |
| Q8IEK7\|Q8IEK7 | Protein ISD11 OS |
| Q8I482\|Q8I482 | Protein HIRA OS |
| Q8IL46\|Q8IL46 | Protein geranylgeranyltransferase type II OS |
| Q8I503\|Q8I503 | Protein farnesyltransferase subunit alpha OS |
| O97253\|O97253 | Protein FAM33A OS |
| C6KTB1\|C6KTB1 | Protein DJ-1 OS |
| C0H4Y6\|C0H4Y6 | Protein disulfide-isomerase OS |
| Q8II88\|Q8II88 | Protein disulfide-isomerase |
| Q9U0K8\|Q9U0K8 | Protein CINCH OS |
| Q8I427\|Q8I427 | Protein CAF40 |
| A0A5K1K8R6\|A0A5K1K8R6 | Protein BCP1 |
| Q8IJT2\|Q8IJT2 | Protein ARV1 |
| Q8ILH4\|Q8ILH4 | Protein archease |
| Q8IER5\|Q8IER5 | Protein AMR3 OS |
| Q8I3S7\|Q8I3S7 | Protein AMR2 OS |
| Q8I0U7\|Q8I0U7 | Proteasome subunit beta type-6 |
| Q8I261\|Q8I261 | Proteasome subunit beta OS |
| A0A5K1K7U1\|A0A5K1K7U1 | Proteasome subunit beta OS |
| Q7K6A9\|Q7K6A9 | Proteasome subunit beta OS |
| Q8IJT1\|Q8IJT1 | Proteasome subunit beta OS |
| Q8I6T3\|Q8I6T3 | Proteasome subunit beta OS |
| Q8IKC9\|Q8IKC9 | Proteasome subunit beta OS |
| O77396\|O77396 | Proteasome subunit alpha type-3 |
| Q8IBI3\|Q8IBI3 | Proteasome subunit alpha type OS |
| Q8IDG3\|Q8IDG3 | Proteasome subunit alpha type OS |
| Q8IDG2\|Q8IDG2 | Proteasome subunit alpha type OS |
| A0A5K1K941\|A0A5K1K941 | Proteasome maturation factor UMP1 |
| C6KST3\|C6KST3 | Proteasome endopeptidase complex OS |
| Q8IAR3\|Q8IAR3 | Proteasome endopeptidase complex OS |
| Q8IK90\|Q8IK90 | Proteasome endopeptidase complex OS |
| Q8IBL7\|Q8IBL7 | Proteasome assembly chaperone 4 |
| Q8I374\|Q8I374 | Proteasome activator 28 subunit beta |
| C0H4H7\|C0H4H7 | Protease |
| Q8I317\|Q8I317 | Protease |
| A0A144A0G8\|A0A144A0G8 | Prolyl-tRNA synthetase OS |
| Q8IL23\|Q8IL23 | Prolyl hydroxylase-like protein |
| Q8I2Q5\|Q8I2Q5 | Proline-tRNA ligase |
| Q7KQJ9\|Q7KQJ9 | Proliferating cell nuclear antigen OS |
| A0A5K1K8U9\|A0A5K1K8U9 | Proliferating cell nuclear antigen OS |
| Q8I1S1\|Q8I1S1 | Prohibitin-like protein PHBL |
| Q8IJP8\|Q8IJP8 | Prohibitin OS |
| Q8IBC3\|Q8IBC3 | Prohibitin OS |
| Q8IJ99\|Q8IJ99 | Probable tRNA N6-adenosine threonylcarbamoyltransferase OS |
| Q8I5V5\|Q8I5V5 | Probable cytosolic iron-sulfur protein assembly protein CIAO1 homolog OS |
| Q8I2Z8\|Q8I2Z8 | Probable ATP-dependent 6-phosphofructokinase OS |
| O96272\|O96272 | PRESAN domain-containing protein OS |
| Q8I207\|Q8I207 | PRESAN domain-containing protein OS |
| Q8I206\|Q8I206 | PRESAN domain-containing protein OS |
| Q8IFL7\|Q8IFL7 | PRESAN domain-containing protein OS |
| Q8IFL1\|Q8IFL1 | PRESAN domain-containing protein OS |
| Q8I3F2\|Q8I3F2 | PRESAN domain-containing protein OS |
| Q8IBE9\|Q8IBE9 | PRESAN domain-containing protein OS |
| A0A143ZWT9\|A0A143ZWT9 | PRESAN domain-containing protein OS |
| A0A143ZZX7\|A0A143ZZX7 | PRESAN domain-containing protein OS |
| Q8I2F4\|Q8I2F4 | PRESAN domain-containing protein OS |
| Q8IK18\|Q8IK18 | PRESAN domain-containing protein OS |
| C6S3C7\|C6S3C7 | PRESAN domain-containing protein OS |
| Q8IJN1\|Q8IJN1 | PRESAN domain-containing protein OS |
| Q8IIX5\|Q8IIX5 | PRESAN domain-containing protein OS |
| Q8IHN7\|Q8IHN7 | PRESAN domain-containing protein OS |
| Q8I636\|Q8I636 | PRESAN domain-containing protein OS |
| Q8I4Q8\|Q8I4Q8 | PRESAN domain-containing protein OS |
| Q8I4P9\|Q8I4P9 | PRESAN domain-containing protein OS |
| Q8IK62\|Q8IK62 | PRESAN domain-containing protein OS |
| Q8IK61\|Q8IK61 | PRESAN domain-containing protein OS |
| Q8IK58\|Q8IK58 | PRESAN domain-containing protein OS |
| Q8IK55\|Q8IK55 | PRESAN domain-containing protein OS |
| Q8I278\|Q8I278 | Pre-rRNA-processing protein TSR2 |
| Q8IKE4\|Q8IKE4 | Pre-rRNA-processing protein PNO1 |
| Q8IBQ1\|Q8IBQ1 | Pre-rRNA-processing protein ESF2 |
| Q8I5B2\|Q8I5B2 | Pre-mRNA-splicing regulator |
| Q8IFP1\|Q8IFP1 | Pre-mRNA-splicing helicase BRR2 |
| A0A144A2S8\|A0A144A2S8 | Pre-mRNA-splicing factor SYF1 |
| C6KSU8\|C6KSU8 | Pre-mRNA-splicing factor SLU |
| Q8IDL7\|Q8IDL7 | Pre-mRNA-splicing factor SLU |
| C6S3D1\|C6S3D1 | Pre-mRNA-splicing factor RDS3 |
| A0A144A0M5\|A0A144A0M5 | Pre-mRNA-splicing factor RBM22 |
| O97334\|O97334 | Pre-mRNA-splicing factor PRP46 |
| Q8IKB8\|Q8IKB8 | Pre-mRNA-splicing factor ISY1 |
| Q8IKW1\|Q8IKW1 | Pre-mRNA-splicing factor CWF18 |
| C6KSY5\|C6KSY5 | Pre-mRNA-splicing factor CWF |
| Q8I4W6\|Q8I4W6 | Pre-mRNA-splicing factor CWC26 |
| Q8IL33\|Q8IL33 | Pre-mRNA-splicing factor CWC24 |
| Q8I540\|Q8I540 | Pre-mRNA-splicing factor CWC22 |
| Q8I3V8\|Q8I3V8 | Pre-mRNA-splicing factor CWC2 |
| Q8I1Z2\|Q8I1Z2 | Pre-mRNA-splicing factor CLF1 |
| Q7KQL1\|Q7KQL1 | Pre-mRNA-splicing factor CEF1 |
| Q8I3N5\|Q8I3N5 | Pre-mRNA-splicing factor BUD31 |
| Q8I2X7\|Q8I2X7 | Pre-mRNA-splicing factor ATP-dependent RNA helicase PRP43 |
| Q8IJA4\|Q8IJA4 | Pre-mRNA-splicing factor ATP-dependent RNA helicase PRP22 |
| Q8I5A4\|Q8I5A4 | Pre-mRNA-splicing factor ATP-dependent RNA helicase PRP2 |
| A0A5K1K9B8\|A0A5K1K9B8 | Pre-mRNA-splicing factor ATP-dependent RNA helicase PRP16 OS |
| Q8IM21\|Q8IM21 | Pre-mRNA-splicing factor 38B |
| Q8I2S9\|Q8I2S9 | Pre-mRNA-splicing factor 18 OS |
| Q8I1X5\|Q8I1X5 | Pre-mRNA-processing-splicing factor 8 |
| O96267\|O96267 | Pre-mRNA-processing protein 45 |
| Q8IIR0\|Q8IIR0 | Pre-mRNA-processing factor 6 |
| A0A5K1K8A8\|A0A5K1K8A8 | Pre-mRNA-processing factor 40 |
| Q8I5L1\|Q8I5L1 | Pre-mRNA-processing factor 1 |
| Q8I416\|Q8I416 | Pre-mRNA-processing ATP-dependent RNA helicase PRP5 |
| Q8IE73\|Q8IE73 | PRELI domain-containing protein |
| Q8I378\|Q8I378 | Prefoldin-like protein |
| Q8I3A4\|Q8I3A4 | Prefoldin subunit 4 OS |
| Q8IBR6\|Q8IBR6 | Prefoldin subunit 3 OS |
| Q8ILS7\|Q8ILS7 | Prefoldin subunit 2 |
| Q8IIS7\|Q8IIS7 | Prefoldin |
| Q8IJS7\|Q8IJS7 | PRE-binding protein OS |
| Q8I2W1\|Q8I2W1 | PPPDE peptidase |
| Q8IJX2\|Q8IJX2 | PPPDE peptidase |
| Q8I5E6\|Q8I5E6 | Potassium channel K1 OS |
| Q7KQK2\|Q7KQK2 | Polyubiquitin OS |
| Q8IB50\|Q8IB50 | Polyribonucleotide 5'-hydroxyl-kinase Clp1 |
| C6S3J5\|C6S3J5 | Polyprenol reductase OS |
| C0H484\|C0H484 | Polynucleotide 5'-triphosphatase OS |
| Q8I518\|Q8I518 | Polyadenylation factor subunit 2 |
| Q8IIS9\|Q8IIS9 | Polyadenylate-binding protein-interacting protein 1 |
| Q8I5H4\|Q8I5H4 | Polyadenylate-binding protein OS |
| Q8I2R8\|Q8I2R8 | Polyadenylate-binding protein 2 |
| Q8IL36\|Q8IL36 | Poly(A)-specific ribonuclease PARN |
| C6KT92\|C6KT92 | Poly(A) polymerase OS |
| Q8I233\|Q8I233 | P-loop containing nucleoside triphosphate hydrolase |
| Q8ILY1\|Q8ILY1 | Plastid replication-repair enzyme OS |
| Q8I224\|Q8I224 | Plasmoredoxin OS |
| Q8IAS0\|Q8IAS0 | Plasmepsin X OS |
| Q8IJ71\|Q8IJ71 | Plasmepsin VII OS |
| Q8I6Z5\|Q8I6Z5 | Plasmepsin V OS |
| Q8ILG2\|Q8ILG2 | Plasmepsin IX OS |
| Q8I6V3\|Q8I6V3 | Plasmepsin II OS |
| A0A144A171\|A0A144A171 | Plasmepsin I OS |
| C0H524\|C0H524 | Plasma membrane protein 1 |
| Q8IE26\|Q8IE26 | PITH domain-containing protein |
| Q8IJU4\|Q8IJU4 | PI31 domain-containing protein |
| Q8I253\|Q8I253 | Photosensitized INA-labeled protein PHIL1 OS |
| C6KT76\|C6KT76 | Phosphotransferase OS |
| Q8IE67\|Q8IE67 | Phosphoribosylpyrophosphate synthetase OS |
| A0A143ZZ61\|A0A143ZZ61 | Phosphopyruvate hydratase OS |
| Q8IAZ8\|Q8IAZ8 | Phosphopantothenoylcysteine decarboxylase |
| Q8IJM5\|Q8IJM5 | Phosphomannomutase OS |
| Q8I5L4\|Q8I5L4 | Phospholipid-transporting ATPase OS |
| Q8I310\|Q8I310 | Phospholipid or glycerol acyltransferase |
| Q8IAY0\|Q8IAY0 | Phospholipase DDHD1 |
| C6KTC8\|C6KTC8 | Phospholipase |
| Q8IDV6\|Q8IDV6 | Phosphoinositide-binding protein PH2 OS |
| Q8IC26\|Q8IC26 | Phosphoinositide-binding protein |
| Q8IJR0\|Q8IJR0 | Phosphoinositide phospholipase C OS |
| Q8IIG6\|Q8IIG6 | Phosphoglycerate mutase OS |
| Q8I1V2\|Q8I1V2 | Phosphoglucomutase-2 OS |
| Q8IDQ9\|Q8IDQ9 | Phosphoethanolamine N-methyltransferase OS |
| Q8I0U9\|Q8I0U9 | Phosphoenolpyruvate/phosphate translocator OS |
| Q8IDR1\|Q8IDR1 | Phosphoenolpyruvate carboxykinase (ATP) OS |
| Q8I6Z7\|Q8I6Z7 | Phosphodiesterase OS |
| Q8II63\|Q8II63 | Phosphoacetylglucosamine mutase |
| C0H5K9\|C0H5K9 | Phosphatidylserine synthase |
| Q8I2N0\|Q8I2N0 | Phosphatidylserine decarboxylase proenzyme OS |
| Q8I239\|Q8I239 | Phosphatidylinositol-4-phosphate 5-kinase OS |
| C6KT29\|C6KT29 | Phosphatidylinositol N-acetylglucosaminyltransferase subunit GPI1 OS |
| C0H532\|C0H532 | Phosphatidylinositol N-acetylglucosaminyltransferase OS |
| Q8I2G7\|Q8I2G7 | Phosphatidylinositol N-acetylglucosaminyltransferase OS |
| Q8IHV1\|Q8IHV1 | Phosphatidylinositol N-acetylglucosaminyltransferase OS |
| Q8I406\|Q8I406 | Phosphatidylinositol 4-kinase OS |
| B9ZSJ6\|B9ZSJ6 | Phosphatidylinositol 4-kinase |
| Q8I3V5\|Q8I3V5 | Phosphatidylinositol 3-kinase OS |
| O77353\|O77353 | Phosphatidylinositol 3- and 4-kinase |
| O77317\|O77317 | Phosphatidate phosphatase OS |
| Q8ILZ6\|Q8ILZ6 | Phosphatidate cytidylyltransferase OS |
| Q8IDS7\|Q8IDS7 | Phosphate transporter OS |
| Q8IDD8\|Q8IDD8 | Phosphatase 2A regulatory subunit-related protein |
| C0H4V3\|C0H4V3 | Phosphatase |
| Q8IJX5\|Q8IJX5 | Phosducin-like protein 3 |
| Q8I3U4\|Q8I3U4 | Phosducin-like protein 1 |
| Q8ILF6\|Q8ILF6 | PhIL1 interacting protein PIP3 OS |
| Q8ILF4\|Q8ILF4 | PhIL1 interacting protein PIP2 OS |
| Q8IDE6\|Q8IDE6 | PhIL1 interacting protein PIP1 OS |
| C6KSN5\|C6KSN5 | Phenylalanyl-tRNA synthetase OS |
| Q8I5A1\|Q8I5A1 | Phenylalanyl-tRNA synthetase OS |
| Q8IIW2\|Q8IIW2 | Phenylalanyl-tRNA synthetase beta subunit OS |
| Q8I246\|Q8I246 | Phenylalanine-tRNA ligase OS |
| Q8IJI4\|Q8IJI4 | PHAX domain-containing protein |
| C6S3B1\|C6S3B1 | PH domain-containing protein |
| Q8I3A7\|Q8I3A7 | PH domain-containing protein |
| C6KTE8\|C6KTE8 | Pfmc-2TM Maurer's cleft two transmembrane protein OS |
| A0A144A022\|A0A144A022 | Pescadillo homolog OS |
| Q8IHP2\|Q8IHP2 | Peroxisome assembly protein 22 |
| Q8IJD0\|Q8IJD0 | Peroxiredoxin OS |
| Q8I5T1\|Q8I5T1 | Peripheral plastid protein 1 |
| Q8IAM3\|Q8IAM3 | Periodic tryptophan protein 2 |
| A0A144A0C0\|A0A144A0C0 | Periodic tryptophan protein 1 |
| A0A5K1K8C6\|A0A5K1K8C6 | Perforin-like protein 5 OS |
| Q8I2S3\|Q8I2S3 | Perforin-like protein 3 OS |
| Q8I4V8\|Q8I4V8 | Peptidylprolyl isomerase OS |
| Q76NN7\|Q76NN7 | Peptidyl-prolyl cis-trans isomerase OS |
| Q8I402\|Q8I402 | Peptidyl-prolyl cis-trans isomerase OS |
| Q8I6S4\|Q8I6S4 | Peptidyl-prolyl cis-trans isomerase OS |
| Q8IIK3\|Q8IIK3 | Peptidyl-prolyl cis-trans isomerase OS |
| Q8I621\|Q8I621 | Peptidyl-prolyl cis-trans isomerase OS |
| Q8I5Q4\|Q8I5Q4 | Peptidyl-prolyl cis-trans isomerase OS |
| Q8IIP5\|Q8IIP5 | Peptidyl-prolyl cis-trans isomerase |
| Q8I372\|Q8I372 | Peptide deformylase OS |
| Q8I1W0\|Q8I1W0 | Peptide chain release factor 2 OS |
| Q8IC21\|Q8IC21 | Peptide chain release factor 2 OS |
| Q8ILH8\|Q8ILH8 | Peptide chain release factor 1 OS |
| Q8IJY3\|Q8IJY3 | Peptidase |
| C6S3D0\|C6S3D0 | Peptidase |
| A0A144A108\|A0A144A108 | Pentatricopeptide repeat domain-containing protein |
| Q8IM30\|Q8IM30 | Pentatricopeptide repeat domain-containing protein |
| O77318\|O77318 | PDZ domain-containing protein OS |
| Q8I400\|Q8I400 | PDCD2 domain-containing protein |
| Q8IBT8\|Q8IBT8 | PDCD2 domain-containing protein |
| O96149\|O96149 | PCI domain-containing protein |
| A0A5K1K9G0\|A0A5K1K9G0 | Patatin-like phospholipase |
| Q8IJB9\|Q8IJB9 | Partial CSTF domain-containing protein |
| Q8I2Q0\|Q8I2Q0 | Parasitophorous vacuolar protein 5 |
| Q8IDW5\|Q8IDW5 | Parasitophorous vacuolar protein 3 |
| Q8I2A1\|Q8I2A1 | Parasite-infected erythrocyte surface protein OS |
| Q8ILP4\|Q8ILP4 | Pantothenate kinase 1 |
| Q8I2T0\|Q8I2T0 | p-aminobenzoic acid synthase OS |
| A0A146M427\|A0A146M427 | Palmitoyltransferase OS |
| O97224\|O97224 | Palmitoyltransferase OS |
| Q8I3I3\|Q8I3I3 | Palmitoyltransferase OS |
| C6KSU5\|C6KSU5 | Palmitoyltransferase OS |
| Q8I2J1\|Q8I2J1 | Palmitoyltransferase OS |
| Q8IEA0\|Q8IEA0 | Palmitoyltransferase OS |
| Q8IIF7\|Q8IIF7 | Palmitoyltransferase OS |
| Q8IBV4\|Q8IBV4 | Palmitoyltransferase DHHC4 |
| Q8I557\|Q8I557 | p25-alpha family protein |
| C6KT97\|C6KT97 | Oxidoreductase |
| Q8IHU8\|Q8IHU8 | OTU domain-containing protein |
| Q8I3Y0\|Q8I3Y0 | Orotate phosphoribosyltransferase OS |
| O96237\|O96237 | Origin recognition complex subunit 5 OS |
| Q8IC17\|Q8IC17 | Origin recognition complex subunit 2 OS |
| A0A144A1W6\|A0A144A1W6 | Origin recognition complex subunit 1 OS |
| Q8IJ96\|Q8IJ96 | Ookinete surface protein P28 OS |
| Q8IHS7\|Q8IHS7 | Oocyst capsule protein Cap93 |
| O96130\|O96130 | Octaprenyl pyrophosphate synthase OS |
| Q8IBM9\|Q8IBM9 | Obg-like ATPase 1 OS |
| O97295\|O97295 | O-acyltransferase OS |
| Q8IK99\|Q8IK99 | NUFIP1 domain-containing protein OS |
| Q8I2V1\|Q8I2V1 | NudC domain-containing protein 1 OS |
| Q8I5I4\|Q8I5I4 | Nucleus export protein BRR6 |
| Q8IJP0\|Q8IJP0 | Nucleotidyltransferase |
| A0A144A2R0\|A0A144A2R0 | Nucleotidyltransferase |
| Q8I2W3\|Q8I2W3 | Nucleosome assembly protein OS |
| Q8I608\|Q8I608 | Nucleosome assembly protein OS |
| Q8IE96\|Q8IE96 | Nucleoside-diphosphatase |
| Q8I2A8\|Q8I2A8 | Nucleoside transporter 4 OS |
| Q8IDM6\|Q8IDM6 | Nucleoside transporter 1 OS |
| Q8ID43\|Q8ID43 | Nucleoside diphosphate kinase OS |
| Q8IL08\|Q8IL08 | Nucleoporin NUP313 |
| Q8I398\|Q8I398 | Nucleoporin NUP221 |
| Q8I3B3\|Q8I3B3 | Nucleoporin NUP138 |
| Q8III3\|Q8III3 | Nucleolar protein 56 |
| Q8IJV7\|Q8IJV7 | Nucleolar protein 5 |
| C6S3I4\|C6S3I4 | Nucleolar protein 10 OS |
| Q8IAL3\|Q8IAL3 | Nucleolar protein 10 |
| Q8I3H9\|Q8I3H9 | Nucleolar preribosomal GTPase |
| Q8ILM2\|Q8ILM2 | Nucleolar GTP-binding protein 2 OS |
| C6KSX2\|C6KSX2 | Nucleolar GTP-binding protein 1 OS |
| Q8IED7\|Q8IED7 | Nucleolar complex protein 4 |
| Q8IDT3\|Q8IDT3 | Nucleolar complex protein 2 |
| O96129\|O96129 | Nucleic acid binding protein |
| Q8II42\|Q8II42 | Nucleic acid binding protein |
| Q8ILX1\|Q8ILX1 | Nuclear transport factor 2 |
| Q8I426\|Q8I426 | Nuclear protein localization protein 4 |
| C6KT67\|C6KT67 | Nuclear polyadenylated RNA-binding protein NAB2 |
| Q8IDW4\|Q8IDW4 | Nuclear movement protein |
| A0A5K1K8D5\|A0A5K1K8D5 | Nuclear import protein MOG1 |
| Q8I1T2\|Q8I1T2 | Nuclear cap-binding protein subunit 2 OS |
| Q8IK04\|Q8IK04 | N-terminal acetyltransferase A complex catalytic subunit ARD1 |
| Q8I405\|Q8I405 | Nse4 |
| C0H4U4\|C0H4U4 | NPL domain-containing protein OS |
| Q8ILS4\|Q8ILS4 | NOT family protein |
| O77328\|O77328 | Non-specific serine/threonine protein kinase OS |
| Q8I1N8\|Q8I1N8 | Non-specific serine/threonine protein kinase OS |
| C6KSQ1\|C6KSQ1 | Non-specific serine/threonine protein kinase OS |
| Q8IIE7\|Q8IIE7 | Non-specific serine/threonine protein kinase OS |
| A0A144A2N6\|A0A144A2N6 | Non-specific serine/threonine protein kinase OS |
| Q8IC06\|Q8IC06 | Non-specific serine/threonine protein kinase OS |
| A0A143ZZK9\|A0A143ZZK9 | Non-SERCA-type Ca2+ -transporting P-ATPase OS |
| Q8IKG9\|Q8IKG9 | NOC3p domain-containing protein OS |
| C0H553\|C0H553 | Nitric oxide synthase |
| C0H4N8\|C0H4N8 | NIMA related kinase 4 OS |
| C0H4G2\|C0H4G2 | NIMA related kinase 2 OS |
| Q8I5D5\|Q8I5D5 | NIMA related kinase 1 OS |
| Q8I266\|Q8I266 | Niemann-Pick type C1-related protein OS |
| O97284\|O97284 | Nicotinamidase |
| Q8IBV9\|Q8IBV9 | Negative elongation factor A |
| Q8I4X8\|Q8I4X8 | NEDD8-conjugating enzyme UBC12 |
| C0H4V1\|C0H4V1 | NEDD8-activating enzyme E1 catalytic subunit OS |
| Q8ILK2\|Q8ILK2 | Nascent polypeptide-associated complex subunit beta OS |
| C6KT55\|C6KT55 | Nascent polypeptide-associated complex subunit alpha |
| Q8I4Y9\|Q8I4Y9 | N-alpha-acetyltransferase 15 |
| Q8IKX3\|Q8IKX3 | NADPH-cytochrome P450 reductase |
| Q8IHW9\|Q8IHW9 | NADPH:adrenodoxin oxidoreductase |
| Q8ID33\|Q8ID33 | NADH-cytochrome b5 reductase |
| C0H4A1\|C0H4A1 | NAD(P)-binding protein |
| Q8ILT3\|Q8ILT3 | N-acyl-phosphatidylethanolamine-hydrolyzing phospholipase D |
| Q8I249\|Q8I249 | N-acetyltransferase |
| Q8IL96\|Q8IL96 | N-acetyltransferase |
| C6KT83\|C6KT83 | N-acetylglucosaminylphosphatidylinositol deacetylase OS |
| Q8IAQ4\|Q8IAQ4 | N-acetylglucosaminyldiphosphodolichol N-acetylglucosaminyltransferase OS |
| Q9U0J7\|Q9U0J7 | N6-L-threonylcarbamoyladenine synthase OS |
| A0A5K1K930\|A0A5K1K930 | N6-adenine-specific methylase |
| Q8ILP8\|Q8ILP8 | Myosin-specific chaperone UNC |
| Q8III1\|Q8III1 | Myosin light chain B OS |
| C6KTA8\|C6KTA8 | Myosin light chain |
| Q8IJM4\|Q8IJM4 | Myosin essential light chain ELC OS |
| Q8I4W8\|Q8I4W8 | Myosin A tail domain interacting protein OS |
| C6KSM2\|C6KSM2 | MYND-type zinc finger protein |
| Q8II81\|Q8II81 | Multiprotein-bridging factor 1 |
| A0A144A1F1\|A0A144A1F1 | Multiple RNA-binding domain-containing protein 1 |
| Q8IM19\|Q8IM19 | Multifunctional methyltransferase subunit TRM112 |
| Q8IKZ6\|Q8IKZ6 | Multidrug resistance protein 2 OS |
| Q7K6A5\|Q7K6A5 | Multidrug resistance protein 1 OS |
| O96245\|O96245 | MtN3-like protein OS |
| Q8IBI2\|Q8IBI2 | mTERF domain-containing protein |
| Q8IDY4\|Q8IDY4 | MSP |
| Q8IDY3\|Q8IDY3 | MSP |
| A0A5K1K987\|A0A5K1K987 | MSP |
| C6KT51\|C6KT51 | mRNA-binding protein PUF3 OS |
| Q8I567\|Q8I567 | mRNA methyltransferase |
| Q8I6Z2\|Q8I6Z2 | mRNA (N6-adenosine)-methyltransferase |
| Q8IC20\|Q8IC20 | mRNA (guanine-N( |
| C6S3I2\|C6S3I2 | MORN repeat-containing protein 5 OS |
| Q8IJ93\|Q8IJ93 | MORN repeat-containing protein 1 OS |
| O96186\|O96186 | Monocarboxylate transporter |
| C0H4B4\|C0H4B4 | MOLO1 domain-containing protein |
| Q8IJQ1\|Q8IJQ1 | MO15-related protein kinase OS |
| C0H4C6\|C0H4C6 | MMS19-like protein |
| Q8IDT7\|Q8IDT7 | Mitotic-spindle organizing protein 1 |
| Q8I5A5\|Q8I5A5 | Mitosis protein dim1 |
| Q7KQK7\|Q7KQK7 | Mitogen-activated protein kinase OS |
| Q8I2I2\|Q8I2I2 | Mitochondrial-processing peptidase subunit beta |
| Q8I3N3\|Q8I3N3 | Mitochondrial-processing peptidase subunit alpha |
| Q8IHZ4\|Q8IHZ4 | Mitochondrial ribosomal protein S9 |
| Q8IBR7\|Q8IBR7 | Mitochondrial ribosomal protein S8 |
| Q8IKJ9\|Q8IKJ9 | Mitochondrial ribosomal protein S6-2 |
| A0A146M1R1\|A0A146M1R1 | Mitochondrial ribosomal protein S5 |
| Q8IEJ8\|Q8IEJ8 | Mitochondrial ribosomal protein S35 |
| Q8IL20\|Q8IL20 | Mitochondrial ribosomal protein S29 |
| Q8IJD1\|Q8IJD1 | Mitochondrial ribosomal protein S22 |
| Q8I5T6\|Q8I5T6 | Mitochondrial ribosomal protein S18 |
| Q8I6T8\|Q8I6T8 | Mitochondrial ribosomal protein S15 |
| Q8I6V1\|Q8I6V1 | Mitochondrial ribosomal protein S14 |
| Q8IKT3\|Q8IKT3 | Mitochondrial ribosomal protein S11 |
| A0A5K1K9F4\|A0A5K1K9F4 | Mitochondrial ribosomal protein L49 |
| C6KTA5\|C6KTA5 | Mitochondrial ribosomal protein L46 |
| C6KSP0\|C6KSP0 | Mitochondrial ribosomal protein L41 |
| Q8IIC6\|Q8IIC6 | Mitochondrial ribosomal protein L3 |
| O97270\|O97270 | Mitochondrial ribosomal protein L29/L4 |
| Q8IKR3\|Q8IKR3 | Mitochondrial ribosomal protein L28 |
| Q8I532\|Q8I532 | Mitochondrial ribosomal protein L23 |
| Q8ILN1\|Q8ILN1 | Mitochondrial ribosomal protein L21 |
| Q8IK97\|Q8IK97 | Mitochondrial ribosomal protein L20 |
| Q9NDU6\|Q9NDU6 | Mitochondrial ribosomal protein L2 |
| C6KSU7\|C6KSU7 | Mitochondrial ribosomal protein L19 |
| Q8ILG7\|Q8ILG7 | Mitochondrial ribosomal protein L15 |
| Q8IIQ5\|Q8IIQ5 | Mitochondrial ribosomal protein L11 |
| Q8ILF5\|Q8ILF5 | Mitochondrial ribosomal protein L1 |
| Q8IDS8\|Q8IDS8 | Mitochondrial pyruvate carrier OS |
| Q8I623\|Q8I623 | Mitochondrial phosphate carrier protein OS |
| A0A143ZZH9\|A0A143ZZH9 | Mitochondrial large subunit ribosomal protein |
| A0A5K1K885\|A0A5K1K885 | Mitochondrial intermediate peptidase |
| Q8IBB5\|Q8IBB5 | Mitochondrial inner membrane protein OXA1 |
| Q8IL53\|Q8IL53 | Mitochondrial inner membrane protease ATP23 |
| C6KT11\|C6KT11 | Mitochondrial import receptor subunit TOM40 |
| Q8I3L8\|Q8I3L8 | Mitochondrial import receptor subunit TOM22 |
| Q8IB71\|Q8IB71 | Mitochondrial import receptor subunit TOM |
| Q8ID24\|Q8ID24 | Mitochondrial import inner membrane translocase subunit TIM9 OS |
| Q8IBI8\|Q8IBI8 | Mitochondrial import inner membrane translocase subunit TIM50 OS |
| Q8IIA9\|Q8IIA9 | Mitochondrial import inner membrane translocase subunit TIM44 |
| Q8IDE0\|Q8IDE0 | Mitochondrial import inner membrane translocase subunit TIM23 |
| C6KTB0\|C6KTB0 | Mitochondrial import inner membrane translocase subunit TIM22 OS |
| Q8I3X2\|Q8I3X2 | Mitochondrial import inner membrane translocase subunit TIM16 |
| Q8IBK8\|Q8IBK8 | Mitochondrial import inner membrane translocase subunit TIM14 |
| Q8I5W2\|Q8I5W2 | Mitochondrial import inner membrane translocase subunit TIM10 |
| Q8ILB7\|Q8ILB7 | Mitochondrial import inner membrane translocase subunit TIM1 |
| Q8I500\|Q8I500 | Mitochondrial import inner membrane translocase subunit OS |
| Q8IE63\|Q8IE63 | Mitochondrial fission 1 protein OS |
| C6KSN2\|C6KSN2 | Mitochondrial chaperone BCS1 |
| Q8I254\|Q8I254 | Mitochondrial carrier protein |
| C0H498\|C0H498 | Mitochondrial carrier protein |
| Q8I397\|Q8I397 | Mitochondrial carrier protein |
| Q8I364\|Q8I364 | Mitochondrial carrier protein |
| Q8I513\|Q8I513 | Mitochondrial carrier protein |
| Q8ID23\|Q8ID23 | Mitochondrial carrier protein |
| C6KSU1\|C6KSU1 | Mitochondrial cardiolipin synthase |
| Q8ILB6\|Q8ILB6 | Mitochondrial acidic protein MAM33 |
| Q8ILX3\|Q8ILX3 | Mini-chromosome maintenance complex-binding protein |
| O77394\|O77394 | Microneme associated antigen OS |
| Q8I578\|Q8I578 | Microgamete surface protein MiGS |
| Q8IE75\|Q8IE75 | Micro-fibrillar-associated protein |
| Q8I5F3\|Q8I5F3 | Mic1 domain-containing protein OS |
| Q8ILY0\|Q8ILY0 | MI domain-containing protein OS |
| C0H494\|C0H494 | Methyltransferase |
| Q8IFN7\|Q8IFN7 | Methyltransferase |
| Q8IHT8\|Q8IHT8 | Methyltransferase |
| Q8IET4\|Q8IET4 | Methyltransferase |
| A0A144A2R4\|A0A144A2R4 | Methyltransferase |
| Q8I716\|Q8I716 | Methionine-tRNA ligase OS |
| Q8IJ60\|Q8IJ60 | Methionine-tRNA ligase OS |
| Q8I3J2\|Q8I3J2 | Methionine aminopeptidase OS |
| Q8ILB8\|Q8ILB8 | Methionine aminopeptidase 2 OS |
| Q8IAP0\|Q8IAP0 | Methionine aminopeptidase 1c |
| Q8IJV0\|Q8IJV0 | Metalloprotease |
| Q8IBL6\|Q8IBL6 | Metallo-hydrolase/oxidoreductase |
| Q8ILT4\|MCA3 | Metacaspase-3 OS |
| Q8IIV4\|Q8IIV4 | Metabolite/drug transporter |
| Q8I2B3\|Q8I2B3 | Merozoite-associated tryptophan-rich antigen OS |
| Q8IJB7\|Q8IJB7 | Merozoite TRAP-like protein OS |
| Q8IJQ4\|Q8IJQ4 | Merozoite surface protein MSA180 OS |
| A0A144A026\|A0A144A026 | Merozoite surface protein 9 OS |
| Q8IJ54\|Q8IJ54 | Merozoite surface protein 6 OS |
| Q7KWJ3\|Q7KWJ3 | Merozoite surface protein 5 OS |
| Q7KWJ2\|Q7KWJ2 | Merozoite surface protein 4 OS |
| Q8IJ55\|Q8IJ55 | Merozoite surface protein 3 OS |
| Q8IJ48\|Q8IJ48 | Merozoite surface protein 11 OS |
| C6KT44\|C6KT44 | Merozoite surface protein 10 OS |
| Q8IDX8\|Q8IDX8 | Merozoite surface protein |
| Q8I0U8\|Q8I0U8 | Merozoite surface antigens OS |
| A0A143ZX41\|A0A143ZX41 | Merozoite surface antigen 2 OS |
| Q8I2Y3\|Q8I2Y3 | Merozoite organizing protein OS |
| C0H4G1\|C0H4G1 | MerC domain-containing protein |
| C0H4B1\|C0H4B1 | Memo-like protein OS |
| Q8IDG8\|Q8IDG8 | Membrane associated histidine-rich protein 2 OS |
| C0H5L9\|C0H5L9 | Membrane associated histidine-rich protein 1 OS |
| C6S3D8\|C6S3D8 | Meiotic recombination protein SPO11-2 |
| Q8IB05\|Q8IB05 | Meiotic recombination protein DMC1 |
| C6S3J7\|C6S3J7 | Meiotic nuclear division protein 1 |
| Q9U0H7\|Q9U0H7 | Mediator of RNA polymerase II transcription subunit 8 |
| Q8IKE0\|Q8IKE0 | Mediator of RNA polymerase II transcription subunit 6 OS |
| Q8IKI6\|Q8IKI6 | Mediator of RNA polymerase II transcription subunit 4 |
| Q8IK88\|Q8IK88 | Mediator of RNA polymerase II transcription subunit 31 OS |
| Q8IIA0\|Q8IIA0 | Mediator of RNA polymerase II transcription subunit 21 |
| Q8IKK5\|Q8IKK5 | Mediator of RNA polymerase II transcription subunit 20 |
| Q8IBZ6\|Q8IBZ6 | Mediator of RNA polymerase II transcription subunit 14 OS |
| C0H4C7\|C0H4C7 | Mediator of RNA polymerase II transcription subunit 11 |
| Q8IC13\|Q8IC13 | Mediator of RNA polymerase II transcription subunit 10 |
| Q8I3Q8\|Q8I3Q8 | Mediator of RNA polymerase II transcription subunit 1 |
| Q8IB55\|Q8IB55 | Mediator of RNA polymerase II transcription subunit |
| Q8IER6\|Q8IER6 | MCL1 domain-containing protein |
| Q8IDS3\|Q8IDS3 | Mannosyltransferase OS |
| Q8ILP1\|Q8ILP1 | Mannose-1-phosphate guanyltransferase |
| Q8I6Z9\|Q8I6Z9 | Malonyl CoA-acyl carrier protein transacylase OS |
| Q8I5V8\|Q8I5V8 | Mago-binding protein |
| A0A143ZWJ6\|A0A143ZWJ6 | Magnesium transporter OS |
| Q8I386\|Q8I386 | Maf-like protein |
| Q8I5C5\|Q8I5C5 | Macrophage migration inhibitory factor OS |
| Q8IEH1\|Q8IEH1 | MACRO domain-containing protein |
| Q8IKQ7\|Q8IKQ7 | MA3 domain-containing protein |
| Q8IEK1\|Q8IEK1 | M1-family alanyl aminopeptidase OS |
| Q8IL11\|Q8IL11 | M1 |
| Q8ILS8\|Q8ILS8 | Lysyl-tRNA synthetase OS |
| Q8I2F3\|Q8I2F3 | Lysophospholipase |
| Q8IK68\|Q8IK68 | Lysophospholipase |
| Q8IDJ8\|Q8IDJ8 | Lysine-tRNA ligase OS |
| C6KSW3\|C6KSW3 | Lsm12 |
| C6KT25\|C6KT25 | L-lactate dehydrogenase OS |
| Q76NM3\|Q76NM3 | L-lactate dehydrogenase OS |
| Q8I632\|Q8I632 | Liver stage associated protein 1 OS |
| A0A143ZZD7\|A0A143ZZD7 | Liver stage antigen 1 OS |
| Q8IEG9\|Q8IEG9 | Lipoate-protein ligase OS |
| Q8I2S0\|Q8I2S0 | Lipoate-protein ligase 2 OS |
| Q8I5X2\|Q8I5X2 | LIMP protein |
| C6KT64\|C6KT64 | Leucyl-tRNA synthetase OS |
| Q8IBB3\|Q8IBB3 | Leucyl-tRNA synthetase OS |
| C6KSW6\|C6KSW6 | Leucine-rich repeat protein OS |
| Q8IID1\|Q8IID1 | Leucine-rich repeat protein OS |
| A0A5K1K981\|A0A5K1K981 | Leucine-rich repeat protein OS |
| A0A144A431\|A0A144A431 | Leucine-rich repeat protein OS |
| Q8IKF4\|Q8IKF4 | Leucine-rich repeat protein OS |
| Q8IL72\|Q8IL72 | Leucine carboxyl methyltransferase 1 OS |
| A0A143ZVJ8\|A0A143ZVJ8 | LEM3/CDC50 family protein |
| Q8IJB1\|Q8IJB1 | LEM3/CDC50 family protein |
| Q8II31\|Q8II31 | LEM3/CDC50 family protein |
| Q8IKW0\|Q8IKW0 | LCCL domain-containing protein OS |
| Q8ILF2\|Q8ILF2 | Large subunit GTPase 1 |
| Q8IKQ3\|Q8IKQ3 | Large ribosomal subunit nuclear export factor |
| O96169\|O96169 | KRR1 small subunit processome component OS |
| Q9TY99\|Q9TY99 | Knob-associated histidine-rich protein OS |
| C0H492\|C0H492 | KLRAQ domain-containing protein OS |
| A0A5K1K999\|A0A5K1K999 | Kinetochore protein SPC25 |
| O77390\|O77390 | Kinetochore protein NUF2 |
| C6KT03\|C6KT03 | Kinetochore protein NDC80 |
| Q8I4Y0\|Q8I4Y0 | Kinesin-13 |
| Q8I4X5\|Q8I4X5 | Kinesin |
| Q8I3W0\|Q8I3W0 | Kinase OS |
| Q8IDQ2\|Q8IDQ2 | Kelch protein K13 OS |
| Q8I5S1\|Q8I5S1 | Kelch domain-containing protein |
| Q8IKF6\|Q8IKF6 | Kelch domain-containing protein |
| Q8IIA6\|Q8IIA6 | Kelch domain-containing protein |
| Q8I3M5\|Q8I3M5 | Karyopherin beta OS |
| Q8IHU4\|Q8IHU4 | J domain-containing protein OS |
| Q8IDZ9\|Q8IDZ9 | Isoleucyl-tRNA synthetase OS |
| Q8I6T2\|Q8I6T2 | Isocitrate dehydrogenase [NADP] OS |
| Q8IKT4\|Q8IKT4 | Iron-sulfur cluster assembly protein OS |
| Q8I3N6\|Q8I3N6 | Iron-sulfur assembly protein OS |
| O96161\|O96161 | Iron-sulfur assembly protein |
| Q8IDH3\|Q8IDH3 | Intron-binding protein aquarius |
| Q8IC15\|Q8IC15 | Inositol-phosphate phosphatase |
| Q8I2U5\|Q8I2U5 | Inosine-5'-monophosphate dehydrogenase OS |
| Q8IKC7\|Q8IKC7 | Inorganic anion exchanger |
| A0A5K1K8W3\|A0A5K1K8W3 | Inner membrane complex suture component |
| Q8IKM6\|Q8IKM6 | Inner membrane complex sub-compartment protein 3 OS |
| Q8IJT5\|Q8IJT5 | Inner membrane complex sub-compartment protein 1 OS |
| Q8I3N7\|Q8I3N7 | Inner membrane complex protein OS |
| A0A5K1K8Y1\|A0A5K1K8Y1 | Inner membrane complex protein OS |
| Q8IJB5\|Q8IJB5 | Inner membrane complex protein 1m |
| Q8IDS1\|Q8IDS1 | Inner membrane complex protein 1k |
| Q8IB69\|Q8IB69 | Inner membrane complex protein 1i |
| Q8I3K7\|Q8I3K7 | Inner membrane complex protein 1g |
| O97229\|O97229 | Inner membrane complex protein 1e |
| Q8IK01\|Q8IK01 | Inner membrane complex protein 1c |
| Q8IKZ9\|Q8IKZ9 | Inner membrane complex protein |
| A0A5K1K8R3\|A0A5K1K8R3 | Indole-3-glycerol-phosphate synthase OS |
| Q8IAY9\|Q8IAY9 | Importin subunit beta |
| Q8IAW0\|Q8IAW0 | Importin subunit alpha OS |
| Q8I2I8\|Q8I2I8 | Importin alpha re-exporter |
| C0H4L1\|C0H4L1 | Importin- |
| Q8IJS1\|Q8IJS1 | Hypoxanthine phosphoribosyltransferase OS |
| Q8I5V3\|Q8I5V3 | Hydroxymethylbilane synthase OS |
| Q8I2N9\|Q8I2N9 | HSP90 co-chaperone p23 OS |
| Q8IKU1\|Q8IKU1 | HSP90 co-chaperone p23 OS |
| Q8IL88\|Q8IL88 | HSP40 |
| C0H473\|C0H473 | HSP20-like chaperone |
| Q8IKY6\|Q8IKY6 | HSP20-like chaperone |
| Q8IKB0\|Q8IKB0 | HSP20-like chaperone |
| A0A144A2J9\|A0A144A2J9 | Hsp |
| Q8I3J0\|Q8I3J0 | Hsc |
| Q8ILD4\|Q8ILD4 | HP12 protein homolog |
| Q8I585\|Q8I585 | Homocysteine S-methyltransferase |
| Q8I1N7\|Q8I1N7 | Holo-[acyl-carrier-protein] synthase |
| Q8IIL1\|Q8IIL1 | Histone-lysine N-methyltransferase SET |
| Q8IDE8\|Q8IDE8 | Histone-lysine N-methyltransferase |
| A0A5K1K956\|A0A5K1K956 | Histone-arginine methyltransferase CARM1 |
| Q8I3R9\|Q8I3R9 | Histone RNA hairpin-binding protein |
| Q8IIV2\|Q8IIV2 | Histone H4 OS |
| Q8IDZ1\|Q8IDZ1 | Histone H3-like centromeric protein CSE4 OS |
| C6KSV0\|C6KSV0 | Histone H3 OS |
| C6KT19\|C6KT19 | Histone H3 OS |
| Q8IBV7\|Q8IBV7 | Histone H2B OS |
| Q8IIV1\|Q8IIV1 | Histone H2B OS |
| O97320\|O97320 | Histone H2A OS |
| C6KT18\|C6KT18 | Histone H2A OS |
| Q7K6A1\|Q7K6A1 | Histone deacetylase OS |
| Q8IJW3\|Q8IJW3 | Histone deacetylase 2 OS |
| A0A144A4T0\|A0A144A4T0 | Histone deacetylase |
| Q8I5H2\|Q8I5H2 | Histone chaperone ASF1 |
| C0H4T2\|C0H4T2 | Histone acetyltransferase subunit NuA4 |
| C0H4A7\|C0H4A7 | Histone acetyltransferase OS |
| Q8III2\|Q8III2 | Histone acetyltransferase OS |
| Q8IB67\|Q8IB67 | Histone acetyltransferase GCN5 OS |
| Q8IL22\|Q8IL22 | Histidine-tRNA ligase OS |
| C0H5M9\|C0H5M9 | Histidine-rich protein III OS |
| Q8I395\|Q8I395 | High molecular weight rhoptry protein 3 OS |
| C0H571\|C0H571 | High molecular weight rhoptry protein 2 OS |
| Q8IB14\|Q8IB14 | High mobility group protein B2 OS |
| Q8I616\|Q8I616 | High mobility group protein B1 OS |
| Q7KWJ5\|Q7KWJ5 | Hexose transporter OS |
| Q8I5K4\|Q8I5K4 | Heterochromatin protein 1 OS |
| Q8IJ78\|Q8IJ78 | Heptatricopeptide repeat-containing protein HPR1 |
| O77348\|O77348 | Heptatricopeptide repeat-containing protein |
| Q8I3N2\|Q8I3N2 | Heptatricopeptide repeat-containing protein |
| Q8I3H1\|Q8I3H1 | Heptatricopeptide repeat-containing protein |
| C6KST1\|C6KST1 | Heptatricopeptide repeat-containing protein |
| C6KT28\|C6KT28 | Heptatricopeptide repeat-containing protein |
| Q8I321\|Q8I321 | Heptatricopeptide repeat-containing protein |
| Q8I2Z9\|Q8I2Z9 | Heptatricopeptide repeat-containing protein |
| Q8I2R5\|Q8I2R5 | Heptatricopeptide repeat-containing protein |
| C0H575\|C0H575 | Heptatricopeptide repeat-containing protein |
| Q8IJX7\|Q8IJX7 | Heptatricopeptide repeat-containing protein |
| Q8IJU6\|Q8IJU6 | Heptatricopeptide repeat-containing protein |
| Q8IHU5\|Q8IHU5 | Heptatricopeptide repeat-containing protein |
| Q8I5J8\|Q8I5J8 | Heptatricopeptide repeat-containing protein |
| C0H5B5\|C0H5B5 | Heptatricopeptide repeat-containing protein |
| A0A5K1K937\|A0A5K1K937 | Heptatricopeptide repeat-containing protein |
| Q8IL39\|Q8IL39 | Heptatricopeptide repeat-containing protein |
| Q8IAY8\|Q8IAY8 | Heptatricopeptide repeat and RAP domain-containing protein |
| Q8IJA7\|Q8IJA7 | Heptatricopeptide repeat and RAP domain-containing protein |
| Q8I5F2\|Q8I5F2 | Heptatricopeptide repeat and RAP domain-containing protein |
| Q8I289\|Q8I289 | Heptatricopeptide repeat and RAP domain-containing protein |
| Q8IKS4\|Q8IKS4 | Hemolysin III OS |
| Q8IJS6\|Q8IJS6 | Heme oxygenase OS |
| Q8I0W6\|Q8I0W6 | Heme O synthase OS |
| A0A144A6G1\|A0A144A6G1 | Heme detoxification protein OS |
| Q8IB94\|Q8IB94 | HECT-like E3 ubiquitin ligase |
| Q7KQK3\|Q7KQK3 | Heat shock protein DNAJ homolog Pfj4 OS |
| Q8IC05\|Q8IC05 | Heat shock protein 90 OS |
| Q8III6\|Q8III6 | Heat shock protein 90 |
| Q8IL32\|Q8IL32 | Heat shock protein 90 |
| Q8IC04\|Q8IC04 | Heat shock protein 86 family protein OS |
| Q8IJN9\|Q8IJN9 | Heat shock protein 60 OS |
| Q8I489\|Q8I489 | Heat shock protein 40 |
| Q8IC01\|Q8IC01 | Heat shock protein 110 OS |
| Q8IIJ8\|Q8IIJ8 | Heat shock protein 101 OS |
| Q8IB24\|Q8IB24 | Heat shock protein |
| Q8I2X4\|Q8I2X4 | Heat shock protein |
| Q8II24\|Q8II24 | Heat shock protein |
| Q8IIF8\|Q8IIF8 | Heat shock factor-binding protein 1 OS |
| Q8I479\|Q8I479 | HCNGP-like protein OS |
| Q8IJ74\|Q8IJ74 | Haloacid dehalogenase-like hydrolase OS |
| Q8I5F6\|Q8I5F6 | Haloacid dehalogenase-like hydrolase |
| Q8I5Z3\|Q8I5Z3 | HAD domain ookinete protein |
| Q8I700\|Q8I700 | H/ACA ribonucleoprotein complex subunit OS |
| Q8ILS0\|Q8ILS0 | H/ACA ribonucleoprotein complex subunit 4 |
| Q8I570\|Q8I570 | H(+)-exporting diphosphatase OS |
| Q8IKR1\|Q8IKR1 | H(+)-exporting diphosphatase OS |
| Q8I2M1\|Q8I2M1 | Guanylate kinase OS |
| Q8IIK2\|Q8IIK2 | Guanine nucleotide-exchange factor SEC12 OS |
| Q8I418\|Q8I418 | Guanidine nucleotide exchange factor OS |
| Q8I720\|Q8I720 | GTP-RNA guanylyltransferase OS |
| Q8IBU4\|Q8IBU4 | GTP-binding translation elongation factor tu family protein |
| Q8IB77\|Q8IB77 | GTP-binding protein Obg2 |
| A0A144A1B2\|A0A144A1B2 | GTP-binding protein Obg1 OS |
| A0A144A297\|A0A144A297 | GTP-binding protein EngA OS |
| C0H4D6\|C0H4D6 | GTP-binding protein |
| C0H5I9\|C0H5I9 | GTP-binding protein |
| Q8ILA0\|Q8ILA0 | GTP-binding protein |
| Q8IKP0\|Q8IKP0 | GTP-binding protein |
| Q8IKL1\|Q8IKL1 | GTP-binding protein |
| C6KSS6\|C6KSS6 | GTP-binding protein |
| Q8IIM7\|Q8IIM7 | GTP-binding protein |
| Q8IL49\|Q8IL49 | GTP-binding protein |
| Q7KQK6\|Q7KQK6 | GTP-binding nuclear protein OS |
| Q8I435\|Q8I435 | GTPase-activating protein |
| Q8I3A9\|Q8I3A9 | GTPase-activating protein |
| Q8I379\|Q8I379 | GTPase-activating protein |
| Q8IIL9\|Q8IIL9 | GTPase-activating protein |
| Q8IKA7\|Q8IKA7 | GTPase-activating protein |
| Q8IAP4\|Q8IAP4 | GTPase-activating protein |
| Q8ILA6\|Q8ILA6 | GTPase Era |
| A0A143ZZW7\|A0A143ZZW7 | GTPase |
| Q8IDL8\|Q8IDL8 | GTPase |
| Q8IIB6\|Q8IIB6 | GrpE protein homolog OS |
| C6KSS2\|C6KSS2 | G-protein associated signal transduction protein |
| Q8IDK1\|Q8IDK1 | GPN-loop GTPase 3 OS |
| Q8I2X6\|Q8I2X6 | GPN-loop GTPase 2 OS |
| Q8I630\|Q8I630 | GPN-loop GTPase 1 OS |
| C6KSZ4\|C6KSZ4 | GPI-anchored wall transfer protein 1 |
| Q8IBB9\|Q8IBB9 | GPI-anchored micronemal antigen OS |
| Q8II76\|Q8II76 | GPI-anchor transamidase |
| A0A5K1K957\|A0A5K1K957 | GPI transamidase subunit PIG-U |
| Q8IIE5\|Q8IIE5 | GPI transamidase component GPI16 |
| Q8I4V9\|Q8I4V9 | GPI mannosyltransferase 2 OS |
| Q8I5U1\|Q8I5U1 | GPI mannosyltransferase 1 OS |
| Q8II19\|Q8II19 | GPI inositol-deacylase OS |
| Q8I5R4\|Q8I5R4 | GPI ethanolamine phosphate transferase 3 |
| Q8IBW3\|Q8IBW3 | GPCR-like receptor SR25 OS |
| Q8I3F4\|Q8I3F4 | G-patch domain-containing protein OS |
| C6KSP5\|C6KSP5 | Glyoxalase I-like protein GILP OS |
| Q8IIM5\|Q8IIM5 | Glyoxalase I OS |
| A0A144A366\|A0A144A366 | Glycylpeptide N-tetradecanoyltransferase OS |
| C0H5L4\|C0H5L4 | Glycosylphosphatidylinositol anchor attachment 1 protein |
| Q8I2Z4\|Q8I2Z4 | Glycolipid transfer protein |
| O77344\|O77344 | Glycogen synthase kinase 3 OS |
| A0A5K1K9F9\|A0A5K1K9F9 | Glyco |
| Q8II35\|Q8II35 | Glycine cleavage system H protein OS |
| Q8IM31\|Q8IM31 | Glycerophosphodiester phosphodiesterase OS |
| Q8IIL4\|Q8IIL4 | Glycerol-3-phosphate dehydrogenase [NAD(+)] OS |
| Q8I5P5\|Q8I5P5 | Glycerol-3-phosphate dehydrogenase [NAD(+)] OS |
| Q8I5S7\|Q8I5S7 | Glycerol-3-phosphate 1-O-acyltransferase OS |
| Q8IKK7\|Q8IKK7 | Glyceraldehyde-3-phosphate dehydrogenase OS |
| Q8I3Y4\|Q8I3Y4 | Glutathione synthetase OS |
| A0A144A1J6\|A0A144A1J6 | Glutathione S-transferase OS |
| A0A144A323\|A0A144A323 | Glutathione reductase OS |
| Q8I5T2\|Q8I5T2 | Glutathione peroxidase OS |
| C6KSR7\|C6KSR7 | Glutaredoxin-like protein OS |
| Q9NLB2\|Q9NLB2 | Glutaredoxin OS |
| C6KTC3\|C6KTC3 | Glutamyl-tRNA(Gln) amidotransferase subunit B OS |
| Q8I1S6\|Q8I1S6 | Glutamyl-tRNA(Gln) amidotransferase subunit A OS |
| Q8IDK7\|Q8IDK7 | Glutamyl-tRNA synthetase OS |
| Q8IDD3\|Q8IDD3 | Glutamyl-tRNA synthetase OS |
| Q8IE10\|Q8IE10 | Glutamine-tRNA ligase OS |
| Q8IJF3\|Q8IJF3 | Glutamine-fructose-6-phosphate transaminase (isomerizing) OS |
| Q8IJR9\|Q8IJR9 | Glutamine amidotransferase OS |
| A0A144A0A4\|A0A144A0A4 | Glutaminase OS |
| Q8IJ56\|Q8IJ56 | Glutamate-rich protein GLURP OS |
| Q8ILF7\|Q8ILF7 | Glutamate dehydrogenase OS |
| Q8ILA4\|Q8ILA4 | Glucose-6-phosphate isomerase OS |
| A0A144A6N0\|A0A144A6N0 | Glucose-6-phosphate dehydrogenase-6-phosphogluconolactonase OS |
| Q8I4Z0\|Q8I4Z0 | Glucose inhibited division protein a homolog |
| C6KTC5\|C6KTC5 | Glucosamine 6-phosphate N-acetyltransferase OS |
| Q8I5I8\|Q8I5I8 | Glideosome-associated protein 45 OS |
| Q8I3V1\|Q8I3V1 | Glideosome-associated protein 40 |
| A0A5K1K8J3\|A0A5K1K8J3 | Glideosome-associated connector OS |
| Q8IM26\|Q8IM26 | Glideosome associated protein with multiple membrane spans 3 OS |
| Q8IFN1\|Q8IFN1 | Glideosome associated protein with multiple membrane spans 2 OS |
| Q8IE80\|Q8IE80 | Glideosome associated protein with multiple membrane spans 1 OS |
| Q8IJ83\|Q8IJ83 | GlcNAc-PI synthesis protein OS |
| O97321\|O97321 | GlcNAc-1-P transferase OS |
| Q8I304\|Q8I304 | GINS complex subunit Psf3 |
| C6KSM5\|C6KSM5 | Geranylgeranyl transferase type-2 subunit beta OS |
| C6S3K4\|C6S3K4 | Geranylgeranyl transferase type-2 subunit beta OS |
| Q8IEG6\|Q8IEG6 | General transcription factor IIH subunit OS |
| Q8I4Y8\|Q8I4Y8 | General transcription factor IIH subunit 4 OS |
| Q8I5U5\|Q8I5U5 | General transcription factor 3C polypeptide 5 |
| Q8I2H7\|Q8I2H7 | General transcription and DNA repair factor IIH helicase subunit XPD OS |
| Q8IAX4\|Q8IAX4 | GDP-mannose 4 |
| A0A143ZXB3\|A0A143ZXB3 | GDP-L-fucose synthase OS |
| O96200\|O96200 | GDP-fructose:GMP antiporter |
| Q8IJ27\|Q8IJ27 | GDP dissociation inhibitor |
| Q8IEU3\|Q8IEU3 | GBPH2 protein OS |
| Q8I2I3\|Q8I2I3 | Gamma-tubulin complex component |
| Q8I2W4\|Q8I2W4 | Gamma-ECS OS |
| Q8I2G4\|Q8I2G4 | Gametocytogenesis-implicated protein OS |
| Q8IAP7\|Q8IAP7 | Gamete release protein |
| Q8IEU2\|Q8IEU2 | Gamete antigen 2 |
| Q8I2L4\|Q8I2L4 | G2 protein |
| Q8IKN0\|Q8IKN0 | FYVE and coiled-coil domain-containing protein OS |
| Q8I2N6\|Q8I2N6 | Fumarate hydratase OS |
| Q8I285\|Q8I285 | FtsJ domain-containing protein OS |
| A0A144A3T1\|A0A144A3T1 | Fructose-bisphosphate aldolase OS |
| C6KT30\|C6KT30 | FPL domain-containing protein OS |
| O77389\|O77389 | Formate-nitrite transporter OS |
| C0H4F1\|C0H4F1 | FoP domain-containing protein |
| Q8IAN2\|Q8IAN2 | Filament assembling protein |
| Q8IEN7\|Q8IEN7 | FHA domain-containing protein |
| Q8I355\|Q8I355 | FHA domain protein |
| C6KT71\|C6KT71 | FeSOD OS |
| Q8IIW9\|Q8IIW9 | FeS cluster assembly protein SufD OS |
| Q8II78\|Q8II78 | Fe-S cluster assembly protein |
| A0A5K1K994\|A0A5K1K994 | Fe-S assembly protein IscX |
| Q8ILW0\|Q8ILW0 | FeS assembly ATPase SufC OS |
| C6KT68\|FENR | Ferredoxin-NADP reductase |
| Q8IED5\|FER | Ferredoxin |
| C0H4S2\|C0H4S2 | Ferlin-like protein |
| Q8IKS2\|Q8IKS2 | Ferlin |
| Q8I5J6\|Q8I5J6 | FbpA domain protein |
| A0A144A1E5\|A0A144A1E5 | F-box only protein 9 homolog |
| C6KSY8\|C6KSY8 | F-box domain-containing protein OS |
| C6KT37\|C6KT37 | F-box domain-containing protein OS |
| Q8IM12\|Q8IM12 | FANCJ-like helicase |
| Q8I333\|Q8I333 | Falstatin OS |
| Q76NL8\|Q76NL8 | Falcilysin OS |
| Q8IAZ0\|Q8IAZ0 | FAD-dependent monooxygenase |
| Q8IL56\|Q8IL56 | FACT complex subunit SSRP1 OS |
| Q8I3T4\|Q8I3T4 | FACT complex subunit OS |
| O77312\|O77312 | Exportin-1 |
| C0H530\|C0H530 | Exportin- |
| Q8IBR9\|Q8IBR9 | Exported serine/threonine protein kinase OS |
| O97308\|O97308 | Exported protein family 4 OS |
| C6KSL4\|C6KSL4 | Exported protein family 3 OS |
| O97462\|O97462 | Exported protein family 1 OS |
| A0A143ZWP5\|A0A143ZWP5 | Exported protein family 1 OS |
| Q8IKC8\|Q8IKC8 | Exported protein 2 OS |
| Q8IIF0\|Q8IIF0 | Exported protein 1 OS |
| Q8IK20\|Q8IK20 | Exported lipase 2 OS |
| Q8IKX8\|Q8IKX8 | Exosome complex exonuclease RRP6 OS |
| Q8IDB6\|Q8IDB6 | Exosome complex exonuclease RRP44 OS |
| Q8ID62\|Q8ID62 | Exosome complex component RRP45 |
| A0A5K1K8M9\|A0A5K1K8M9 | Exosome complex component RRP42 |
| Q8ILI7\|Q8ILI7 | Exosome complex component RRP41 OS |
| Q8IEP5\|Q8IEP5 | Exosome complex component RRP40 |
| Q9U0I4\|Q9U0I4 | Exosome complex component RRP4 OS |
| O96177\|O96177 | Exosome complex component MTR3 |
| Q8IBQ2\|Q8IBQ2 | Exosome complex component CSL4 |
| C0H520\|C0H520 | Exoribonuclease II OS |
| Q8I358\|Q8I358 | Exoribonuclease |
| A0A5K1K8K9\|A0A5K1K8K9 | Exoribonuclease |
| Q8IKP4\|Q8IKP4 | Exonuclease V |
| Q8IB75\|Q8IB75 | Exonuclease |
| A0A143ZZN7\|A0A143ZZN7 | Exonuclease |
| A0A5K1K919\|A0A5K1K919 | Exonuclease |
| Q8IE00\|Q8IE00 | Eukaryotic translation initiation factor 6 OS |
| Q8I603\|Q8I603 | Eukaryotic translation initiation factor 5A OS |
| Q8I5X8\|Q8I5X8 | Eukaryotic translation initiation factor 5 |
| O97266\|O97266 | Eukaryotic translation initiation factor 4E OS |
| Q8IHT2\|Q8IHT2 | Eukaryotic translation initiation factor 4C OS |
| Q8I1Q5\|Q8I1Q5 | Eukaryotic translation initiation factor 3 subunit M OS |
| Q9NFE6\|Q9NFE6 | Eukaryotic translation initiation factor 3 subunit K OS |
| Q8IBT2\|Q8IBT2 | Eukaryotic translation initiation factor 3 subunit I OS |
| Q8IAZ3\|Q8IAZ3 | Eukaryotic translation initiation factor 3 subunit G OS |
| Q8I2X0\|Q8I2X0 | Eukaryotic translation initiation factor 3 subunit F |
| Q8I3I5\|Q8I3I5 | Eukaryotic translation initiation factor 3 subunit E OS |
| Q8IJW4\|Q8IJW4 | Eukaryotic translation initiation factor 3 subunit D OS |
| Q8I5Y3\|Q8I5Y3 | Eukaryotic translation initiation factor 3 subunit C OS |
| Q8I3T1\|Q8I3T1 | Eukaryotic translation initiation factor 3 subunit B OS |
| Q8I5S6\|Q8I5S6 | Eukaryotic translation initiation factor 3 subunit A OS |
| Q8IJL2\|Q8IJL2 | Eukaryotic translation initiation factor 2A OS |
| Q8IJT9\|Q8IJT9 | Eukaryotic translation initiation factor 2 subunit beta OS |
| Q8IBH7\|Q8IBH7 | Eukaryotic translation initiation factor 2 subunit alpha OS |
| C0H5B1\|C0H5B1 | Eukaryotic translation initation factor 4 gamma OS |
| O96203\|O96203 | Eukaryotic peptide chain release factor subunit 1 OS |
| Q8IFN9\|Q8IFN9 | Eukaryotic initiation factor 4A-III |
| Q8IKF0\|Q8IKF0 | Eukaryotic initiation factor 4A OS |
| Q8IDM2\|Q8IDM2 | Ethanolamine-phosphate cytidylyltransferase OS |
| Q8IIB7\|Q8IIB7 | Ethanolamine kinase OS |
| Q8IJ22\|Q8IJ22 | Esterase |
| Q8I4R0\|Q8I4R0 | Esterase |
| Q8IM74\|Q8IM74 | Esterase |
| Q8IIR2\|Q8IIR2 | Essential nuclear protein 1 |
| Q8IC35\|Q8IC35 | Erythrocyte membrane-associated antigen OS |
| O96110\|O96110 | Erythrocyte membrane protein 1 (PfEMP1) |
| A0A143ZXL1\|A0A143ZXL1 | Erythrocyte membrane protein 1 (PfEMP1) |
| Q9NFB4\|Q9NFB4 | Erythrocyte membrane protein 1 |
| Q8I218\|Q8I218 | Erythrocyte membrane protein 1 |
| Q9U0G6\|Q9U0G6 | Erythrocyte membrane protein 1 |
| Q8IFQ5\|Q8IFQ5 | Erythrocyte membrane protein 1 |
| Q8IFQ4\|Q8IFQ4 | Erythrocyte membrane protein 1 |
| Q8IBX0\|Q8IBX0 | Erythrocyte membrane protein 1 |
| Q8IBW9\|Q8IBW9 | Erythrocyte membrane protein 1 |
| Q8IAS3\|Q8IAS3 | Erythrocyte membrane protein 1 |
| Q8IAS7\|Q8IAS7 | Erythrocyte membrane protein 1 |
| Q8I2E4\|Q8I2E4 | Erythrocyte membrane protein 1 |
| Q8I519\|Q8I519 | Erythrocyte membrane protein 1 |
| Q8I409\|Q8I409 | ERCC4 domain-containing protein |
| O96136\|O96136 | ERCC1 nucleotide excision repair protein |
| Q8ILM8\|Q8ILM8 | ERAD-associated E3 ubiquitin-protein ligase HRD1 OS |
| Q8IHW7\|Q8IHW7 | ER membrane protein complex subunit 8 |
| C0H4A3\|C0H4A3 | ER membrane protein complex subunit 6 OS |
| Q8I5R2\|Q8I5R2 | ER membrane protein complex subunit 6 OS |
| Q9NLB0\|Q9NLB0 | ER membrane protein complex subunit 5 |
| Q8ILB0\|Q8ILB0 | ER membrane protein complex subunit 4 OS |
| Q8IDA3\|Q8IDA3 | ER membrane protein complex subunit 3 OS |
| Q8ILZ5\|Q8ILZ5 | ER membrane protein complex subunit 2 OS |
| Q8IAU7\|Q8IAU7 | ER membrane protein complex subunit 1 OS |
| Q8IEL0\|Q8IEL0 | ER membrane protein complex subunit |
| Q8IE22\|Q8IE22 | ER lumen protein retaining receptor 1 |
| Q8I4X4\|Q8I4X4 | Epsin-like protein |
| C6KSZ2\|C6KSZ2 | Enoyl-acyl carrier reductase OS |
| Q8IJ30\|Q8IJ30 | Enhancer of rudimentary homolog OS |
| Q8I0V4\|Q8I0V4 | Endoplasmin |
| Q8IIR7\|Q8IIR7 | Endoplasmic reticulum-resident calcium binding protein OS |
| Q8IIC3\|Q8IIC3 | Endoplasmic reticulum oxidoreductin |
| C0H5H0\|C0H5H0 | Endoplasmic reticulum chaperone GRP1 |
| A0A143ZZC6\|A0A143ZZC6 | Endonuclease/exonuclease/phosphatase family protein |
| Q8IEI6\|Q8IEI6 | EMP1-trafficking protein OS |
| Q8I5E0\|Q8I5E0 | Elongator complex protein 3 |
| Q8I251\|Q8I251 | Elongation of fatty acids protein OS |
| C6KSQ7\|C6KSQ7 | Elongation of fatty acids protein OS |
| Q8IE20\|Q8IE20 | Elongation factor Tu OS |
| Q8IDL6\|Q8IDL6 | Elongation factor Tu |
| C6KSM4\|C6KSM4 | Elongation factor G OS |
| Q8I592\|Q8I592 | Elongation factor G |
| Q8IKW5\|Q8IKW5 | Elongation factor 2 OS |
| A0A5K1K967\|A0A5K1K967 | Elongation factor 1-gamma |
| O97319\|O97319 | Elongation factor 1-delta |
| Q8I320\|Q8I320 | Elongation factor 1-beta OS |
| Q8I0P6\|Q8I0P6 | Elongation factor 1-alpha OS |
| Q8I257\|Q8I257 | ELM2 domain-containing protein OS |
| Q8I3Y9\|Q8I3Y9 | EKC/KEOPS complex subunit CGI121 OS |
| Q8IBB6\|Q8IBB6 | eIF-2B GDP-GTP exchange factor subunit alpha OS |
| C6KT69\|C6KT69 | EGF-like membrane protein |
| Q8I318\|Q8I318 | EFP domain-containing protein OS |
| Q8IJF4\|Q8IJF4 | EF-hand calcium-binding domain-containing protein |
| Q8IKJ8\|Q8IKJ8 | EF-hand calcium-binding domain-containing protein |
| Q8I3R2\|Q8I3R2 | EELM2 domain-containing protein |
| Q8I2F9\|Q8I2F9 | Early transcribed membrane protein OS |
| A0A5K1K7X4\|A0A5K1K7X4 | Early transcribed membrane protein 5 OS |
| Q8IFM9\|Q8IFM9 | Early transcribed membrane protein 4 OS |
| O96128\|O96128 | Early transcribed membrane protein 2 OS |
| Q7KQM5\|Q7KQM5 | Early transcribed membrane protein 14.1 OS |
| Q8IIX3\|Q8IIX3 | Early transcribed membrane protein 11.2 OS |
| Q7KQK8\|Q7KQK8 | Early transcribed membrane protein 11.1 OS |
| Q8IJM9\|Q8IJM9 | Early transcribed membrane protein 10.3 OS |
| Q8IK21\|Q8IK21 | Early transcribed membrane protein 10.1 OS |
| O77367\|O77367 | E3 ubiquitin-protein ligase RBX1 |
| O77347\|O77347 | E3 ubiquitin-protein ligase OS |
| C0H4K6\|C0H4K6 | E3 ubiquitin-protein ligase OS |
| Q8IJZ4\|Q8IJZ4 | E3 ubiquitin-protein ligase |
| O77387\|O77387 | E3 ubiquitin-protein ligase |
| A0A5K1K9G1\|A0A5K1K9G1 | E3 SUMO-protein ligase PIAS |
| Q8IKW3\|Q8IKW3 | E3 SUMO-protein ligase NSE2 |
| Q8I657\|Q8I657 | E2F-associated phosphoprotein |
| Q8IDD9\|Q8IDD9 | E2 ubiquitin-conjugating enzyme OS |
| Q8ILB9\|Q8ILB9 | Dynein-related AAA-type ATPase |
| Q8IIM3\|Q8IIM3 | Dynein light chain Tctex-type |
| C6S3D2\|C6S3D2 | Dynein light chain roadblock OS |
| C6S3A9\|C6S3A9 | Dynein light chain OS |
| Q8IBG2\|Q8IBG2 | Dynein light chain OS |
| Q8I5R9\|Q8I5R9 | Dynein light chain OS |
| Q8IDD2\|Q8IDD2 | Dynein light chain OS |
| Q8IB59\|Q8IB59 | Dynein light chain 1 OS |
| C0H568\|C0H568 | Dynein light chain |
| C6S3G7\|C6S3G7 | Dynein light chain |
| A0A143ZYP5\|A0A143ZYP5 | Dynein heavy chain |
| Q8IJE8\|Q8IJE8 | Dynein |
| Q8IJ32\|Q8IJ32 | Dynamin-like protein OS |
| Q8IHR4\|Q8IHR4 | Dynamin-like protein OS |
| Q8I5M3\|Q8I5M3 | Dynamin-like protein |
| Q8IBL8\|Q8IBL8 | Dynactin subunit 5 |
| Q8I419\|Q8I419 | DUF66 |
| Q8I5W1\|Q8I5W1 | DUF4460 domain-containing protein OS |
| C6KTE1\|C6KTE1 | DUF4205 domain-containing protein OS |
| Q8I546\|Q8I546 | DUF4149 domain-containing protein OS |
| Q8I1T7\|Q8I1T7 | DUF2431 domain-containing protein OS |
| C0H5E6\|C0H5E6 | DUF12 |
| O77334\|O77334 | Dual specificity protein phosphatase OS |
| Q8I4S1\|Q8I4S1 | dTMP kinase OS |
| Q8IBT1\|Q8IBT1 | Drug/metabolite transporter DMT2 OS |
| Q8I4Z1\|Q8I4Z1 | Double C2-like domain-containing protein OS |
| Q8IJT8\|Q8IJT8 | Dolichyl-phosphate-mannose-protein mannosyltransferase |
| Q8IIK0\|Q8IIK0 | Dolichyl-diphosphooligosaccharide-protein glycotransferase OS |
| C6S3L2\|C6S3L2 | Dolichyl-diphosphooligosaccharide-protein glycosyltransferase subunit OST5 OS |
| C0H4P5\|C0H4P5 | Dolichyl-diphosphooligosaccharide-protein glycosyltransferase subunit OST2 OS |
| O77351\|O77351 | Dolichyl-diphosphooligosaccharide-protein glycosyltransferase subunit 1 OS |
| Q8I2V7\|Q8I2V7 | Dolichyl-diphosphooligosaccharide-protein glycosyltransferase 48 kDa subunit OS |
| Q8IHU9\|Q8IHU9 | Dolichol-phosphate mannosyltransferase subunit 1 OS |
| Q8ILP7\|Q8ILP7 | DNL-type zinc finger protein OS |
| O96122\|O96122 | DnaJ protein |
| O96276\|O96276 | DnaJ protein |
| Q8I3N0\|Q8I3N0 | DnaJ protein |
| C6KT47\|C6KT47 | DnaJ protein |
| Q8IAQ5\|Q8IAQ5 | DnaJ protein |
| Q8IB72\|Q8IB72 | DnaJ protein |
| Q8I2W2\|Q8I2W2 | DnaJ protein |
| A0A143ZZE4\|A0A143ZZE4 | DnaJ protein |
| Q8IHZ6\|Q8IHZ6 | DnaJ protein |
| Q8IEP3\|Q8IEP3 | DnaJ protein |
| Q8IDD5\|Q8IDD5 | DnaJ protein |
| A0A144A181\|A0A144A181 | DnaJ protein |
| Q8ILY2\|Q8ILY2 | DnaJ protein |
| Q8ILV6\|Q8ILV6 | DnaJ protein |
| C6S3I0\|C6S3I0 | DnaJ protein |
| Q8IKA6\|Q8IKA6 | DnaJ protein |
| Q8IIR6\|Q8IIR6 | DnaJ homolog subfamily C member 16 OS |
| Q8ILU4\|Q8ILU4 | DNA-directed RNA polymerases I and III subunit RPAC2 |
| Q8IHT3\|Q8IHT3 | DNA-directed RNA polymerases I and III subunit RPAC1 |
| O77315\|O77315 | DNA-directed RNA polymerases I |
| Q8IC08\|Q8IC08 | DNA-directed RNA polymerases I |
| Q8I5R8\|Q8I5R8 | DNA-directed RNA polymerases I |
| Q8ID59\|Q8ID59 | DNA-directed RNA polymerases I |
| Q8IDR2\|Q8IDR2 | DNA-directed RNA polymerases I |
| O96159\|O96159 | DNA-directed RNA polymerase subunit OS |
| O77375\|O77375 | DNA-directed RNA polymerase subunit OS |
| Q8IE49\|Q8IE49 | DNA-directed RNA polymerase subunit OS |
| O96236\|O96236 | DNA-directed RNA polymerase subunit beta OS |
| Q8II17\|Q8II17 | DNA-directed RNA polymerase subunit beta OS |
| Q8I5X9\|Q8I5X9 | DNA-directed RNA polymerase subunit beta OS |
| Q8IEN9\|Q8IEN9 | DNA-directed RNA polymerase subunit alpha |
| Q8I410\|Q8I410 | DNA-directed RNA polymerase OS |
| Q8IIB0\|Q8IIB0 | DNA-directed RNA polymerase OS |
| Q8IIV5\|Q8IIV5 | DNA-directed RNA polymerase III subunit RPC8 |
| A0A144A2J0\|A0A144A2J0 | DNA-directed RNA polymerase III subunit RPC6 OS |
| Q8IKP3\|Q8IKP3 | DNA-directed RNA polymerase III subunit RPC5 |
| Q8IKK2\|Q8IKK2 | DNA-directed RNA polymerase III subunit RPC4 |
| Q8I241\|Q8I241 | DNA-directed RNA polymerase II subunit RPB9 |
| Q8IM54\|Q8IM54 | DNA-directed RNA polymerase II subunit RPB4 |
| Q8I2S6\|Q8I2S6 | DNA-directed RNA polymerase II subunit RPB3 |
| Q8IJC9\|Q8IJC9 | DNA-directed RNA polymerase II subunit RPB |
| O96150\|O96150 | DNA-directed RNA polymerase II 16 kDa subunit |
| Q8IKB1\|Q8IKB1 | DNA-directed RNA polymerase |
| O96139\|O96139 | DNA-directed DNA polymerase OS |
| C6KTD8\|C6KTD8 | DNA-directed DNA polymerase OS |
| Q8I5N9\|Q8I5N9 | DNA-binding chaperone |
| Q8IJU3\|Q8IJU3 | DNA2/NAM |
| Q8IKG2\|Q8IKG2 | DNA/RNA-binding protein KIN1 |
| A0A5K1K8Y8\|A0A5K1K8Y8 | DNA/RNA-binding protein Alba 4 OS |
| Q8IJX8\|Q8IJX8 | DNA/RNA-binding protein Alba 3 OS |
| Q8IDN4\|Q8IDN4 | DNA/RNA-binding protein Alba 2 OS |
| Q8IAX8\|Q8IAX8 | DNA/RNA-binding protein Alba 1 OS |
| Q8I617\|Q8I617 | DNA/RNA-binding protein |
| O97240\|O97240 | DNA-(apurinic or apyrimidinic site) endonuclease OS |
| Q8IDM7\|Q8IDM7 | DNA topoisomerase OS |
| Q8I528\|Q8I528 | DNA topoisomerase 2 OS |
| Q8ILC8\|Q8ILC8 | DNA topoisomerase 2 OS |
| Q8I0X3\|Q8I0X3 | DNA topoisomerase (ATP-hydrolyzing) OS |
| Q8I3J5\|Q8I3J5 | DNA replication licensing factor MCM3 |
| Q8ILR7\|Q8ILR7 | DNA replication licensing factor MCM2 OS |
| Q8IC16\|Q8IC16 | DNA replication licensing factor MCM |
| O77383\|O77383 | DNA replication complex GINS protein |
| Q8IIS8\|Q8IIS8 | DNA repair protein RAD51 homolog OS |
| A0A143ZWE7\|A0A143ZWE7 | DNA repair protein RAD14 |
| Q8ID22\|Q8ID22 | DNA repair endonuclease XPF |
| A0A144A659\|A0A144A659 | DNA primase OS |
| C0H531\|C0H531 | DNA primase large subunit OS |
| Q9U0H1\|Q9U0H1 | DNA polymerase OS |
| Q7KQL4\|Q7KQL4 | DNA polymerase OS |
| Q8I579\|Q8I579 | DNA polymerase II subunit 2 OS |
| O77321\|O77321 | DNA polymerase delta small subunit |
| Q8IKK3\|Q8IKK3 | DNA polymerase alpha subunit B OS |
| C6KT89\|C6KT89 | DNA polymerase 1 |
| Q8IBJ3\|Q8IBJ3 | DNA mismatch repair protein PMS1 |
| Q8I447\|Q8I447 | DNA mismatch repair protein OS |
| Q8IIJ0\|Q8IIJ0 | DNA mismatch repair protein MLH OS |
| Q8IES4\|Q8IES4 | DNA ligase OS |
| Q8IAR1\|Q8IAR1 | DNA helicase PSH3 OS |
| Q8I5T4\|Q8I5T4 | DNA helicase OS |
| Q8IEE5\|Q8IEE5 | DNA helicase OS |
| Q8IDF0\|Q8IDF0 | DNA helicase OS |
| Q8IJ31\|Q8IJ31 | DNA helicase OS |
| Q8I5E7\|Q8I5E7 | DNA helicase 60 OS |
| Q8IM03\|Q8IM03 | DNA damage-inducible protein 1 |
| Q8ILP0\|Q8ILP0 | DIX domain-containing protein |
| Q8I3M7\|Q8I3M7 | Divalent metal transporter |
| C6KSS8\|C6KSS8 | Diphthine methyltransferase |
| Q8IJV5\|Q8IJV5 | Diphthine methyl ester synthase OS |
| Q8I5J0\|Q8I5J0 | Diphthamide synthase OS |
| Q8I5N0\|Q8I5N0 | Diphthamide biosynthesis protein 3 |
| Q8ILG9\|Q8ILG9 | Diphthamide biosynthesis protein 2 |
| A0A144A2G5\|A0A144A2G5 | Dipeptidyl aminopeptidase 1 OS |
| C0H5K7\|C0H5K7 | DIP13 homolog |
| Q8I5A0\|Q8I5A0 | Dihydrolipoyl dehydrogenase OS |
| Q8IJJ4\|Q8IJJ4 | Dihydrolipoamide acetyltransferase component of pyruvate dehydrogenase complex OS |
| Q8IE69\|Q8IE69 | Dihydrofolate synthase/folylpolyglutamate synthase OS |
| Q8IB73\|Q8IB73 | Dicarboxylate/tricarboxylate carrier OS |
| Q8ILP6\|Q8ILP6 | Diadenosine tetraphosphate synthetase OS |
| Q8IKC5\|Q8IKC5 | Diacylglycerol kinase OS |
| O97275\|O97275 | Derlin OS |
| Q8IJ82\|Q8IJ82 | Derlin OS |
| Q8IKF2\|Q8IKF2 | Derlin OS |
| Q8IL34\|Q8IL34 | Dephospho-CoA kinase |
| Q8II92\|Q8II92 | Deoxyuridine 5'-triphosphate nucleotidohydrolase OS |
| Q8I0W8\|Q8I0W8 | Deoxyribodipyrimidine photo-lyase |
| Q8ILW8\|Q8ILW8 | Deoxyhypusine synthase OS |
| Q8I701\|Q8I701 | Deoxyhypusine hydroxylase OS |
| Q8IL68\|Q8IL68 | Delta-aminolevulinic acid dehydratase OS |
| Q8I5K9\|Q8I5K9 | Debranching enzyme-associated ribonuclease |
| Q8IIT7\|Q8IIT7 | DEAD/DEAH box helicase |
| Q8IE72\|Q8IE72 | DEAD box helicase |
| Q8IEH9\|Q8IEH9 | DEAD box helicase |
| Q9U0H4\|Q9U0H4 | DEAD box ATP-dependent RNA helicase |
| Q9U0J4\|Q9U0J4 | DDB1- and CUL4-associated factor 13 OS |
| Q8IKV0\|Q8IKV0 | DCB domain-containing protein OS |
| Q8IIS0\|DTD | D-aminoacyl-tRNA deacylase OS |
| Q8IC39\|Q8IC39 | Cytosolic iron-sulfur assembly component 2 |
| Q8I344\|Q8I344 | Cytosolic Fe-S cluster assembly factor NBP35 |
| C6KSY3\|C6KSY3 | Cytosolic Fe-S cluster assembly factor NAR1 |
| C0H4M2\|C0H4M2 | Cytoskeleton associated protein |
| Q8IL60\|Q8IL60 | Cytoplasmic tRNA 2-thiolation protein 2 |
| Q8I6U9\|Q8I6U9 | Cytochrome c1 |
| O97246\|O97246 | Cytochrome c oxidase subunit ApiCOX35 |
| Q8I2Z5\|Q8I2Z5 | Cytochrome c oxidase subunit ApiCOX30 |
| Q8IL73\|Q8IL73 | Cytochrome c oxidase subunit ApiCOX26 |
| Q8IKI8\|Q8IKI8 | Cytochrome c oxidase subunit ApiCOX25 |
| A0A5K1K922\|A0A5K1K922 | Cytochrome c oxidase subunit ApiCOX24 |
| Q8IM67\|Q8IM67 | Cytochrome c oxidase subunit ApiCOX19 |
| Q8I3N1\|Q8I3N1 | Cytochrome c oxidase subunit ApiCOX18 |
| A0A5K1K8V5\|A0A5K1K8V5 | Cytochrome c oxidase subunit ApiCOX14 |
| Q8I2M9\|Q8I2M9 | Cytochrome c oxidase subunit 6B |
| Q8I2N1\|Q8I2N1 | Cytochrome c oxidase subunit 5B |
| A0A5K1K9B3\|A0A5K1K9B3 | Cytochrome c oxidase subunit 2 |
| Q8I6V2\|Q8I6V2 | Cytochrome c oxidase subunit 2 |
| Q8IJE6\|Q8IJE6 | Cytochrome c oxidase copper chaperone |
| Q8I627\|Q8I627 | Cytochrome c oxidase assembly protein COX19 |
| Q8ILB4\|Q8ILB4 | Cytochrome c oxidase assembly protein COX15 |
| C6S3H9\|C6S3H9 | Cytochrome c oxidase assembly protein COX14 |
| Q8IK85\|Q8IK85 | Cytochrome c oxidase assembly protein COX11 |
| Q8IB89\|Q8IB89 | Cytochrome c oxidase assembly factor 5 |
| Q8I609\|Q8I609 | Cytochrome c heme lyase OS |
| Q8I5H1\|Q8I5H1 | Cytochrome c heme lyase OS |
| Q8I6T6\|Q8I6T6 | Cytochrome c |
| Q8IM53\|Q8IM53 | Cytochrome c |
| Q8IL75\|Q8IL75 | Cytochrome b-c1 complex subunit Rieske |
| C0H4H6\|C0H4H6 | Cytochrome b-c1 complex subunit 9 |
| Q8ILJ5\|Q8ILJ5 | Cytochrome b-c1 complex subunit 6 OS |
| Q8IJS2\|Q8IJS2 | Cytochrome b-c1 complex subunit |
| C0H542\|C0H542 | Cytochrome b5-like heme/steroid binding protein |
| Q8I599\|Q8I599 | Cytochrome b5 |
| Q8I2G2\|Q8I2G2 | Cytoadherence linked asexual protein 9 OS |
| Q8I5Z9\|Q8I5Z9 | Cytidine deaminase |
| Q8IJP3\|Q8IJP3 | Cysteinyl-tRNA synthetase OS |
| Q8IM47\|Q8IM47 | Cysteine-rich small secreted protein CSS |
| A0A143ZVI9\|A0A143ZVI9 | Cysteine-rich secretory protein |
| Q8IFM8\|CYRPA | Cysteine-rich protective antigen OS |
| C6S3G5\|C6S3G5 | Cysteine-rich PDZ-binding protein OS |
| Q8I6U4\|Q8I6U4 | Cysteine proteinase falcipain 2a OS |
| Q8I6V0\|Q8I6V0 | Cysteine proteinase falcipain 1 OS |
| O96155\|O96155 | Cysteine desulfuration protein SufE OS |
| Q8IBT4\|Q8IBT4 | Cysteine desulfurase OS |
| Q8IBI5\|Q8IBI5 | Cysteine desulfurase OS |
| Q8I283\|Q8I283 | Cyclin-dependent kinases regulatory subunit OS |
| Q8I2S1\|Q8I2S1 | Cyclin-dependent kinases regulatory subunit OS |
| Q76NM8\|Q76NM8 | Cyclin OS |
| Q8IAM4\|Q8IAM4 | Cyclin N-terminal domain-containing protein OS |
| Q8IKK0\|Q8IKK0 | Cyclin 1 OS |
| C0H483\|C0H483 | Cyclic amine resistance locus protein OS |
| Q8I288\|Q8I288 | Cyclase-associated protein OS |
| Q8I2D9\|Q8I2D9 | CX3CL1-binding protein 1 OS |
| Q8IKW9\|Q8IKW9 | cwf21 domain-containing protein OS |
| C6KTD3\|C6KTD3 | Cullin-like protein |
| Q8IAU5\|Q8IAU5 | Cullin-1 |
| Q8IDB7\|Q8IDB7 | CUGBP Elav-like family member 1 OS |
| Q8ILZ3\|Q8ILZ3 | CTP synthase OS |
| Q8I0W1\|Q8I0W1 | CSTF domain-containing protein |
| Q8I4T2\|Q8I4T2 | CSC1-like protein |
| O77391\|O77391 | CS domain-containing protein OS |
| Q8IAW7\|Q8IAW7 | CS domain protein |
| Q8IKY1\|Q8IKY1 | Crossover junction endonuclease MUS81 |
| C6KTA0\|C6KTA0 | CRAL/TRIO domain-containing protein |
| Q8I2U6\|Q8I2U6 | CRAL/TRIO domain-containing protein |
| Q8II87\|Q8II87 | CRAL/TRIO domain-containing protein |
| C6KT79\|C6KT79 | CPW-WPC family protein OS |
| C6KTD5\|C6KTD5 | CPW-WPC family protein OS |
| C0H5G0\|C0H5G0 | CPW-WPC family protein OS |
| O77380\|O77380 | CPSF (Cleavage and polyadenylation specific factor) |
| Q8I3X5\|Q8I3X5 | CPSF |
| A0A143ZXB4\|A0A143ZXB4 | COX assembly mitochondrial protein OS |
| Q8IHU1\|Q8IHU1 | Coproporphyrinogen oxidase OS |
| Q8ILN2\|Q8ILN2 | Copper transporter OS |
| Q8IL79\|Q8IL79 | Copper transporter OS |
| A0A144A0I2\|A0A144A0I2 | COPI associated protein |
| Q8IKI7\|Q8IKI7 | Conserved oligomeric Golgi complex subunit 6 OS |
| C6KSZ9\|C6KSZ9 | Conserved oligomeric Golgi complex subunit 4 |
| Q8IDX3\|Q8IDX3 | Conserved oligomeric Golgi complex subunit 3 |
| C0H598\|C0H598 | Condensin complex subunit 2 OS |
| C6S3H2\|C6S3H2 | Condensin complex subunit 1 |
| Q8II59\|Q8II59 | Component of oligomeric Golgi complex 2 OS |
| Q8I248\|Q8I248 | Cold-shock protein |
| C0H4Y8\|C0H4Y8 | Coiled-coil domain-containing protein 124 |
| C0H4S7\|C0H4S7 | Coenzyme Q-binding protein COQ10 homolog |
| Q8I1T3\|Q8I1T3 | Coatomer subunit zeta OS |
| Q8IHR6\|Q8IHR6 | Coatomer subunit gamma OS |
| Q8II16\|Q8II16 | Coatomer subunit delta OS |
| Q8ILG6\|Q8ILG6 | Coatomer subunit beta |
| C6KSR5\|C6KSR5 | Coatomer alpha subunit |
| Q8IBU9\|Q8IBU9 | c-Myc-binding protein |
| Q8I562\|Q8I562 | Clustered-asparagine-rich protein OS |
| Q8IEH7\|Q8IEH7 | Clu domain-containing protein OS |
| Q8II53\|Q8II53 | Cleft lip and palate transmembrane protein 1-like protein OS |
| C6KT45\|C6KT45 | Cleavage stimulation factor subunit 1 |
| Q8I0V9\|Q8I0V9 | Cleavage and polyadenylation specificity factor subunit 5 OS |
| Q8ILQ1\|Q8ILQ1 | Cleavage and polyadenylation specificity factor subunit 4 |
| Q8IL83\|Q8IL83 | Cleavage and polyadenylation specificity factor subunit 3 |
| Q8IB25\|Q8IB25 | Cleavage and polyadenylation specificity factor subunit 2 OS |
| O77371\|O77371 | Cleavage and polyadenylation specificity factor |
| Q8IJA3\|Q8IJA3 | Claudin-like apicomplexan microneme protein |
| Q8ILA9\|Q8ILA9 | Clathrin light chain OS |
| Q8I5L6\|Q8I5L6 | Clathrin heavy chain OS |
| Q8IKY3\|Q8IKY3 | CLASP domain-containing protein |
| C6KST9\|C6KST9 | Citrate synthase-like protein |
| Q7K740\|Q7K740 | Circumsporozoite protein OS |
| Q8I2J9\|Q8I2J9 | Cilia- and flagella-associated protein 299 OS |
| Q8IHV7\|Q8IHV7 | CID domain-containing protein OS |
| Q8IJG6\|Q8IJG6 | Chromodomain-helicase-DNA-binding protein 1 homolog |
| Q8IIW0\|Q8IIW0 | Chromatin remodeling protein OS |
| Q8IE52\|Q8IE52 | Chromatin assembly factor 1 subunit B |
| Q8I238\|Q8I238 | Chromatin assembly factor 1 protein WD40 domain |
| Q8ILD0\|Q8ILD0 | Chromatin assembly factor 1 P55 subunit |
| C6KT66\|C6KT66 | Chorismate synthase OS |
| Q8IJI6\|Q8IJI6 | Chorein |
| Q8IEE9\|Q8IEE9 | Choline-phosphate cytidylyltransferase OS |
| C6KTB9\|C6KTB9 | Choline/ethanolaminephosphotransferase |
| Q8IM71\|Q8IM71 | Choline kinase OS |
| Q8I4R4\|Q8I4R4 | Chitinase OS |
| Q8I6S7\|Q8I6S7 | Chitinase |
| Q8I573\|Q8I573 | CHCH domain-containing protein OS |
| Q9U0J2\|Q9U0J2 | Chaperone protein DnaJ OS |
| Q8IB03\|Q8IB03 | Chaperone protein ClpB1 OS |
| C0H546\|C0H546 | Chaperone |
| Q8I719\|Q8I719 | cGMP-dependent protein kinase OS |
| Q8IC02\|Q8IC02 | Cg8 protein OS |
| Q8IBZ5\|Q8IBZ5 | Cg |
| C6KSN6\|C6KSN6 | Centrosomal protein CEP |
| Q8I714\|Q8I714 | Centrin-4 OS |
| Q8IJC7\|Q8IJC7 | Centrin-3 OS |
| Q8IL07\|Q8IL07 | Centrin-2 OS |
| Q8I272\|Q8I272 | Centrin-1 OS |
| Q8I5P1\|Q8I5P1 | Cell traversal protein for ookinetes and sporozoites OS |
| C6KT34\|C6KT34 | Cell division cycle protein 48 homolog |
| Q8I3H2\|Q8I3H2 | Cell cycle regulator protein |
| Q8I5L0\|Q8I5L0 | Cell cycle associated protein |
| A0A5K1K996\|A0A5K1K996 | CDT1-like protein |
| C0H5B7\|C0H5B7 | CDP-diacylglycerol-inositol 3-phosphatidyltransferase OS |
| Q8IB37\|Q8IB37 | CDP-diacylglycerol-glycerol-3-phosphate 3-phosphatidyltransferase OS |
| Q8I3Y3\|Q8I3Y3 | CDK-activating kinase assembly factor MAT1 OS |
| Q9NDU7\|Q9NDU7 | CDGSH iron-sulfur domain-containing protein |
| Q8IJH6\|Q8IJH6 | CDGSH iron-sulfur domain-containing protein |
| C6KSZ6\|C6KSZ6 | Cdc2-related protein kinase 5 OS |
| O77385\|O77385 | Cdc2-related protein kinase 4 OS |
| Q8I1T4\|Q8I1T4 | Cdc2-related protein kinase 3 OS |
| O97247\|O97247 | CCT-beta OS |
| Q8II43\|Q8II43 | CCT-alpha OS |
| A0A143ZX72\|A0A143ZX72 | CCR4-NOT transcription complex subunit 5 |
| Q8I569\|Q8I569 | CCR4-NOT transcription complex subunit 4 |
| Q8ILC3\|Q8ILC3 | CCR4-associated factor 16 |
| C0H4T9\|C0H4T9 | CCR4-associated factor 1 OS |
| Q8IEN4\|Q8IEN4 | CCAAT-binding transcription factor |
| Q8I461\|Q8I461 | Cation transporting P-ATPase OS |
| Q8IBH9\|Q8IBH9 | Cation transporting ATPase |
| Q8I1Y2\|Q8I1Y2 | Cathepsin J OS |
| Q8I0V1\|Q8I0V1 | Cathepsin J OS |
| Q8IIW5\|Q8IIW5 | Casein kinase II subunit beta OS |
| Q8IIR9\|Q8IIR9 | Casein kinase 2 |
| A0A144A308\|A0A144A308 | Casein kinase 1 OS |
| Q8IHW5\|Q8IHW5 | Carbonic anhydrase OS |
| Q8IEN3\|Q8IEN3 | Carbamoyl phosphate synthetase OS |
| Q7KQK0\|Q7KQK0 | cAMP-dependent protein kinase regulatory subunit OS |
| Q7K6A0\|Q7K6A0 | cAMP-dependent protein kinase catalytic subunit OS |
| A0A5K1K8Q4\|A0A5K1K8Q4 | Calpain OS |
| C0H4A2\|C0H4A2 | Calmodulin-like protein OS |
| Q8IL29\|Q8IL29 | Calmodulin-like protein OS |
| A0A144A1Y0\|A0A144A1Y0 | Calmodulin OS |
| Q8ILR3\|Q8ILR3 | Calmodulin |
| Q8I542\|Q8I542 | Calcyclin-binding protein OS |
| C0H586\|C0H586 | Calcyclin-binding protein |
| Q76NN8\|Q76NN8 | Calcium-transporting ATPase OS |
| A0A5K1K8H0\|A0A5K1K8H0 | Calcium-dependent protein kinase 5 OS |
| A0A143ZWW4\|A0A143ZWW4 | Calcium-dependent protein kinase 1 OS |
| Q8IID2\|Q8IID2 | Calcium-dependent protein kinase |
| C6KSQ2\|C6KSQ2 | Calcium-binding protein |
| Q8IKV9\|Q8IKV9 | Calcineurin subunit B OS |
| Q8IAN8\|Q8IAN8 | Cactin homolog |
| Q8IHP6\|Q8IHP6 | CAAX farnesyltransferase subunit beta OS |
| C0H487\|C0H487 | C2H2-type domain-containing protein OS |
| A0A143ZYA8\|A0A143ZYA8 | C2 domain-containing protein OS |
| A0A144A4I8\|A0A144A4I8 | BTB/POZ domain-containing protein |
| O97305\|O97305 | BSD domain-containing protein OS |
| Q8I5S4\|Q8I5S4 | Bromodomain protein 2 |
| Q8IJ72\|Q8IJ72 | Bromodomain protein 1 OS |
| Q8IB26\|Q8IB26 | BRIX domain |
| A0A5K1K8R9\|A0A5K1K8R9 | BRCT domain-containing protein OS |
| Q8I3V0\|Q8I3V0 | BolA-like protein |
| Q8I0V6\|Q8I0V6 | Blood stage antigen 41-3 OS |
| C0H4F3\|C0H4F3 | Bis(5'-nucleosyl)-tetraphosphatase [asymmetrical] OS |
| Q8IKN1\|Q8IKN1 | Biotin-protein ligase 2 OS |
| Q8II79\|Q8II79 | Bifunctional farnesyl/geranylgeranyl diphosphate synthase OS |
| Q8I1R6\|Q8I1R6 | Bifunctional dihydrofolate reductase-thymidylate synthase OS |
| Q8IJT3\|Q8IJT3 | BET1-like protein |
| A0A143ZYA4\|A0A143ZYA4 | BEM46-like protein |
| Q8IIC2\|Q8IIC2 | BEACH domain-containing protein |
| Q8I446\|Q8I446 | BCNT-C domain-containing protein OS |
| Q8I4U9\|Q8I4U9 | Bax inhibitor 1 |
| Q8IKN3\|Q8IKN3 | Bax inhibitor 1 |
| C0H4K4\|C0H4K4 | Basal complex transmembrane protein 2 OS |
| C6KSW1\|C6KSW1 | Basal complex transmembrane protein 1 OS |
| Q8ILA2\|Q8ILA2 | Basal complex protein BCP1 OS |
| Q8I3A2\|Q8I3A2 | Bacterial histone-like protein OS |
| Q8IJK2\|Q8IJK2 | Autophagy-related protein OS |
| A0A143ZWA3\|A0A143ZWA3 | Autophagy-related protein 3 |
| Q8IJR6\|Q8IJR6 | Autophagy-related protein 18 OS |
| A0A143ZXF2\|A0A143ZXF2 | Autophagy-related protein 11 |
| Q8ILG0\|Q8ILG0 | Autophagy protein 5 OS |
| A0A144A1E4\|A0A144A1E4 | Atypical protein kinase |
| Q8IIH1\|Q8IIH1 | ATP-dependent zinc metalloprotease FTSH |
| Q8IKI9\|Q8IKI9 | ATP-dependent zinc metalloprotease FTSH |
| Q9TY94\|Q9TY94 | ATP-dependent RNA helicase UAP56 OS |
| Q8IJI8\|Q8IJI8 | ATP-dependent RNA helicase ROK1 |
| Q8IL21\|Q8IL21 | ATP-dependent RNA helicase MAK5 |
| C6KTE4\|C6KTE4 | ATP-dependent RNA helicase HAS1 OS |
| Q8IB47\|Q8IB47 | ATP-dependent RNA helicase DHX36 |
| Q8IET8\|Q8IET8 | ATP-dependent RNA helicase DHR1 |
| Q8I3B4\|Q8I3B4 | ATP-dependent RNA helicase DDX60 |
| Q8I1X9\|Q8I1X9 | ATP-dependent RNA helicase DDX51 |
| Q8IL13\|Q8IL13 | ATP-dependent RNA helicase DDX5 |
| A0A5K1K8X8\|A0A5K1K8X8 | ATP-dependent RNA helicase DDX41 |
| O96264\|O96264 | ATP-dependent RNA helicase DDX4 |
| A0A5K1K868\|A0A5K1K868 | ATP-dependent RNA helicase DDX23 |
| Q8I459\|Q8I459 | ATP-dependent RNA helicase DDX2 |
| Q8I511\|Q8I511 | ATP-dependent RNA helicase DBP9 |
| Q8IEP2\|Q8IEP2 | ATP-dependent RNA helicase DBP6 |
| Q8IKP1\|Q8IKP1 | ATP-dependent RNA helicase DBP5 OS |
| C6S3H8\|C6S3H8 | ATP-dependent RNA helicase DBP4 |
| Q8IBA2\|Q8IBA2 | ATP-dependent RNA helicase DBP10 |
| Q8IBN8\|Q8IBN8 | ATP-dependent RNA helicase DBP |
| C6KSM1\|C6KSM1 | ATP-dependent RNA helicase |
| Q8I5B6\|Q8I5B6 | ATP-dependent protease subunit ClpQ OS |
| Q8I377\|Q8I377 | ATP-dependent protease ATPase subunit ClpY OS |
| Q8ILU7\|Q8ILU7 | ATP-dependent protease |
| A0A144A0Z6\|A0A144A0Z6 | ATP-dependent DNA/RNA helicase PSH2 OS |
| Q8IM28\|Q8IM28 | ATP-dependent Clp protease regulatory subunit ClpC OS |
| O97252\|O97252 | ATP-dependent Clp protease proteolytic subunit OS |
| Q8IL98\|Q8IL98 | ATP-dependent Clp protease proteolytic subunit OS |
| Q8IEB2\|Q8IEB2 | ATP-dependent Clp protease adapter protein ClpS OS |
| C6KTA3\|C6KTA3 | ATPase OS |
| Q8I469\|Q8I469 | ATPase |
| Q8IIN7\|Q8IIN7 | ATPase |
| Q8IKK6\|Q8IKK6 | ATPase |
| Q8I267\|Q8I267 | ATP synthase-associated protein |
| Q9NLB1\|Q9NLB1 | ATP synthase-associated protein |
| C6KSV8\|C6KSV8 | ATP synthase-associated protein |
| C6KT41\|C6KT41 | ATP synthase-associated protein |
| Q8IAZ1\|Q8IAZ1 | ATP synthase-associated protein |
| C0H4X8\|C0H4X8 | ATP synthase-associated protein |
| Q8I2H5\|Q8I2H5 | ATP synthase-associated protein |
| Q8IJG2\|Q8IJG2 | ATP synthase-associated protein |
| A0A144A102\|A0A144A102 | ATP synthase-associated protein |
| Q8IHT7\|Q8IHT7 | ATP synthase-associated protein |
| Q8IET5\|Q8IET5 | ATP synthase-associated protein |
| A0A5K1K943\|A0A5K1K943 | ATP synthase-associated protein |
| C6S3H7\|C6S3H7 | ATP synthase-associated protein |
| Q8IEL5\|Q8IEL5 | ATP synthase subunit O |
| Q8I6T7\|Q8I6T7 | ATP synthase subunit gamma OS |
| Q8IBU5\|Q8IBU5 | ATP synthase subunit epsilon |
| Q8IHP4\|Q8IHP4 | ATP synthase subunit delta |
| C0H4L0\|C0H4L0 | ATP synthase subunit C |
| Q8I0V2\|Q8I0V2 | ATP synthase subunit beta OS |
| O96252\|O96252 | ATP synthase subunit alpha OS |
| C6S3D4\|C6S3D4 | ATP synthase mitochondrial F1 complex assembly factor 2 |
| Q8I5V1\|Q8I5V1 | ATP synthase mitochondrial F1 complex assembly factor 1 |
| O77349\|O77349 | ATP synthase F0 subunit d-like protein |
| Q8IIB2\|Q8IIB2 | ATP synthase F0 subunit b-like protein |
| Q8IBR0\|Q8IBR0 | ATP synthase F0 subunit a-like protein |
| Q8IIA3\|Q8IIA3 | ATG12-activating enzyme E1 ATG |
| Q8I2B1\|Q8I2B1 | Aspartyl-tRNA synthetase OS |
| Q8I3W4\|Q8I3W4 | Aspartate-tRNA ligase |
| A0A5K1K910\|A0A5K1K910 | Aspartate carbamoyltransferase OS |
| O96198\|O96198 | Asparagine-tRNA ligase OS |
| Q8I408\|Q8I408 | Asparagine-tRNA ligase OS |
| Q8I5A3\|Q8I5A3 | Asparagine-rich protein |
| C0H4A5\|C0H4A5 | Armadillo-domain containing rhoptry protein OS |
| Q8I5M2\|Q8I5M2 | Arginyl-tRNA synthetase OS |
| Q8I313\|Q8I313 | Arginine-tRNA ligase OS |
| Q8II36\|Q8II36 | Aquaglyceroporin OS |
| Q8IKY5\|Q8IKY5 | Appr-1-p processing domain protein OS |
| Q8I359\|Q8I359 | Apoptosis-related protein OS |
| C0H4E4\|C0H4E4 | Apicortin |
| Q8I3H4\|Q8I3H4 | Apicoplast TIC22 protein OS |
| Q8IIU2\|Q8IIU2 | Apicoplast ribosomal protein S15 |
| Q8IHZ0\|Q8IHZ0 | Apicoplast ribosomal protein S14p/S29e |
| Q8IKM3\|Q8IKM3 | Apicoplast ribosomal protein S10 |
| C0H4T5\|C0H4T5 | Apicoplast ribosomal protein L33 |
| Q8I5W7\|Q8I5W7 | Apicoplast ribosomal protein L29 |
| Q8ILH3\|Q8ILH3 | Apicoplast ribosomal protein L15 |
| Q8IJJ0\|Q8IJJ0 | Apicoplast integral membrane protein |
| Q8IHS0\|Q8IHS0 | Apicoplast import protein Tic20 |
| Q8I3Z0\|Q8I3Z0 | Apical rhoptry neck protein OS |
| Q8I1P1\|Q8I1P1 | Apical merozoite protein OS |
| Q7KQK5\|Q7KQK5 | Apical membrane antigen 1 OS |
| Q8IFN2\|Q8IFN2 | Apical asparagine-rich protein AARP OS |
| Q8IIH2\|Q8IIH2 | AP-4 complex subunit mu |
| Q8I3A8\|Q8I3A8 | AP-4 complex subunit epsilon |
| Q8IL63\|Q8IL63 | AP-3 complex subunit mu |
| C0H4T0\|C0H4T0 | AP-3 complex subunit delta |
| C6KSX7\|C6KSX7 | AP-3 complex subunit beta |
| C6KSN9\|C6KSN9 | AP2 domain transcription factor OS |
| C6KT65\|C6KT65 | AP2 domain transcription factor AP2Tel OS |
| Q8IKY0\|Q8IKY0 | AP2 domain transcription factor AP2-O5 |
| A0A5K1K8Z4\|A0A5K1K8Z4 | AP2 domain transcription factor AP2-O4 |
| Q8IHT5\|Q8IHT5 | AP2 domain transcription factor AP2-O |
| Q8IJW6\|Q8IJW6 | AP2 domain transcription factor AP2-I OS |
| C6KSY0\|C6KSY0 | AP2 domain transcription factor |
| Q8IAM1\|Q8IAM1 | AP2 domain transcription factor |
| Q8I2H4\|Q8I2H4 | AP2 domain transcription factor |
| Q8IIS4\|Q8IIS4 | AP2 domain transcription factor |
| Q8IIK9\|Q8IIK9 | AP2 domain transcription factor |
| Q8I531\|Q8I531 | AP2 domain transcription factor |
| Q8IHX2\|Q8IHX2 | AP2 domain transcription factor |
| Q8IER4\|Q8IER4 | AP2 domain transcription factor |
| Q8I5M5\|Q8I5M5 | AP-2 complex subunit mu |
| Q8IEK4\|Q8IEK4 | AP-1 complex subunit mu-1 OS |
| Q8IKS3\|Q8IKS3 | AP-1 complex subunit gamma |
| O96254\|O96254 | AP complex subunit sigma OS |
| Q8IFN5\|Q8IFN5 | AP complex subunit sigma OS |
| Q8III7\|Q8III7 | AP complex subunit sigma OS |
| A0A144A372\|A0A144A372 | AP complex subunit sigma OS |
| Q8I3I6\|Q8I3I6 | AP complex subunit beta OS |
| Q8IBF8\|Q8IBF8 | AP complex subunit beta OS |
| Q8IJ28\|Q8IJ28 | Antigen UB05 OS |
| C6KTA7\|C6KTA7 | Ankyrin-repeat protein |
| Q8IJU0\|Q8IJU0 | Ankyrin-repeat protein |
| Q8ILY7\|Q8ILY7 | Ankyrin-repeat protein |
| C6KT81\|C6KT81 | Anaphase-promoting complex subunit 11 OS |
| Q8I5N2\|Q8I5N2 | Anaphase-promoting complex subunit 10 |
| Q8IBH6\|Q8IBH6 | Anaphase-promoting complex subunit 1 OS |
| Q8I460\|Q8I460 | AN1-type zinc finger protein |
| Q8IJV2\|Q8IJV2 | AMP-binding domain-containing protein OS |
| A0A144A2H0\|A0A144A2H0 | Aminopeptidase P OS |
| C0H5G8\|C0H5G8 | Aminomethyltransferase |
| Q8IKV4\|Q8IKV4 | Aminomethyltransferase |
| Q9U0L4\|Q9U0L4 | Aminoacyl-tRNA hydrolase OS |
| C6KSV1\|C6KSV1 | Aminoacyl-tRNA hydrolase OS |
| C6KTD0\|C6KTD0 | Amino acid transporter AAT1 OS |
| Q8I5A6\|Q8I5A6 | Amino acid transporter |
| Q8IEK5\|Q8IEK5 | Alternative splicing regulator |
| Q8IIG9\|Q8IIG9 | Alternative splicing factor ASF-1 |
| Q8I0X0\|Q8I0X0 | Alpha-soluble NSF attachment protein |
| Q8IIG3\|Q8IIG3 | Alpha/beta hydrolase fold domain containing protein |
| Q8I1Z1\|Q8I1Z1 | Alpha/beta hydrolase |
| Q8IBH1\|Q8IBH1 | Alpha/beta hydrolase |
| Q8IB95\|Q8IB95 | Alpha/beta hydrolase |
| C6S3G2\|C6S3G2 | Alpha/beta hydrolase |
| Q8ILZ4\|Q8ILZ4 | Alpha/beta hydrolase |
| Q8IB97\|Q8IB97 | Alkyl transferase OS |
| Q8IM05\|Q8IM05 | Aldo-keto reductase |
| Q8ID61\|Q8ID61 | Aldehyde reductase |
| Q8ID31\|Q8ID31 | Alanine-tRNA ligase OS |
| Q8I1X7\|Q8I1X7 | Ag-1 blood stage membrane protein homolog OS |
| C0H4F6\|C0H4F6 | AFG1-like ATPase |
| Q8I5R0\|Q8I5R0 | Adrenodoxin-type ferredoxin |
| A0A143ZY58\|A0A143ZY58 | ADP-ribosylation factor OS |
| Q8I4Y5\|Q8I4Y5 | ADP-ribosylation factor GTPase-activating protein OS |
| Q8IEF2\|Q8IEF2 | ADP-ribosylation factor |
| Q8IDA7\|Q8IDA7 | ADP-ribosylation factor |
| Q8IL50\|Q8IL50 | ADP-ribosylation factor |
| Q8IJ34\|Q8IJ34 | ADP/ATP transporter on adenylate translocase OS |
| Q8IJY9\|Q8IJY9 | ADP/ATP carrier protein |
| C0H4R1\|C0H4R1 | Adenylyl cyclase beta OS |
| Q7KWJ4\|Q7KWJ4 | Adenylosuccinate lyase OS |
| C0H582\|C0H582 | Adenylate kinase-like protein 2 OS |
| Q8IJV6\|Q8IJV6 | Adenylate kinase OS |
| Q8IB06\|Q8IB06 | Adenylate kinase 2 OS |
| Q8IJA9\|Q8IJA9 | Adenosine deaminase OS |
| C6KSQ0\|C6KSQ0 | Acylphosphatase-like domain-containing protein OS |
| O97330\|O97330 | Acyl-CoA synthetase OS |
| Q8I3L4\|Q8I3L4 | Acyl-CoA synthetase OS |
| Q8I535\|Q8I535 | Acyl-CoA synthetase OS |
| Q7KQL7\|Q7KQL7 | Acyl-CoA synthetase OS |
| Q8IK57\|Q8IK57 | Acyl-CoA binding protein OS |
| Q8IAT5\|Q8IAT5 | Acyl-CoA binding protein |
| Q8I1U2\|Q8I1U2 | Acylated pleckstrin-homology domain-containing protein |
| Q7KWJ1\|Q7KWJ1 | Acyl carrier protein OS |
| Q8I0V5\|Q8I0V5 | Acyl carrier protein |
| O97256\|O97256 | Activator of Hsp90 ATPase |
| Q8IIH9\|Q8IIH9 | Actin-related protein 2/3 complex subunit 1 |
| Q8IBQ9\|Q8IBQ9 | Actin-related protein |
| Q8ILM5\|Q8ILM5 | Actin-related protein |
| Q8IIW6\|Q8IIW6 | Actin-like protein |
| A0A143ZXR3\|A0A143ZXR3 | Actin-like protein |
| A0A144A3N9\|A0A144A3N9 | Actin II OS |
| A0A144A1R5\|A0A144A1R5 | Actin I OS |
| Q8IDR8\|Q8IDR8 | Aconitate hydratase OS |
| Q8I2X3\|Q8I2X3 | Acid phosphatase OS |
| C6KTB4\|C6KTB4 | Acetyl-CoA synthetase |
| Q8IKE1\|Q8IKE1 | Acetyl-CoA carboxylase OS |
| Q8IKW7\|Q8IKW7 | Acetyl-CoA acetyltransferase |
| Q8IAT7\|Q8IAT7 | ABC1 family |
| O97278\|O97278 | ABC transporter I family member 1 |
| Q8IAX3\|Q8IAX3 | ABC transporter F family member 1 OS |
| Q8I6Z4\|Q8I6Z4 | ABC transporter E family member 1 |
| Q8IDH9\|Q8IDH9 | ABC transporter B family member 6 |
| Q8IER1\|Q8IER1 | AAR2 protein |
| C0H4M1\|C0H4M1 | AAA family ATPase |
| Q8IAN5\|Q8IAN5 | AAA family ATPase |
| C0H4S0\|C0H4S0 | AAA family ATPase |
| Q8IAX9\|Q8IAX9 | AAA family ATPase |
| Q8ILW7\|Q8ILW7 | AAA family ATPase |
| C6KTB6\|C6KTB6 | 6-pyruvoyltetrahydropterin synthase OS |
| Q8IKT2\|Q8IKT2 | 6-phosphogluconate dehydrogenase |
| Q8IAU3\|Q8IAU3 | 6-hydroxymethyl- |
| Q8IKH0\|Q8IKH0 | 60S ribosome subunit biogenesis protein NIP |
| Q8I441\|Q8I441 | 60S ribosomal subunit protein L24 |
| Q8IE85\|Q8IE85 | 60S ribosomal protein L6 |
| A0A5K1K8V8\|A0A5K1K8V8 | 60S ribosomal protein L6 |
| Q8ILL3\|Q8ILL3 | 60S ribosomal protein L5 |
| Q8I431\|Q8I431 | 60S ribosomal protein L4 OS |
| C0H4H3\|C0H4H3 | 60S ribosomal protein L39 OS |
| Q8II62\|Q8II62 | 60S ribosomal protein L38 OS |
| Q8I713\|Q8I713 | 60S ribosomal protein L36 OS |
| Q8IHT9\|Q8IHT9 | 60S ribosomal protein L35ae |
| Q8IIB4\|Q8IIB4 | 60S ribosomal protein L35 |
| Q8IBY4\|Q8IBY4 | 60S ribosomal protein L34 OS |
| Q8I3B0\|Q8I3B0 | 60S ribosomal protein L32 OS |
| Q8I463\|Q8I463 | 60S ribosomal protein L31 OS |
| Q8IJK8\|Q8IJK8 | 60S ribosomal protein L30e |
| Q8IJC6\|Q8IJC6 | 60S ribosomal protein L3 OS |
| A0A143ZYX3\|A0A143ZYX3 | 60S ribosomal protein L3 |
| Q8IHU0\|Q8IHU0 | 60S ribosomal protein L28 OS |
| O77364\|O77364 | 60S ribosomal protein L26 |
| Q8IEM3\|Q8IEM3 | 60S ribosomal protein L24 |
| Q8IE82\|Q8IE82 | 60S ribosomal protein L23 OS |
| Q8IE09\|Q8IE09 | 60S ribosomal protein L23 |
| Q8IB51\|Q8IB51 | 60S ribosomal protein L22 |
| Q8ILK3\|Q8ILK3 | 60S ribosomal protein L21 OS |
| Q8I3T9\|Q8I3T9 | 60S ribosomal protein L2 OS |
| C6KT23\|C6KT23 | 60S ribosomal protein L2 |
| Q8IKM5\|Q8IKM5 | 60S ribosomal protein L2 |
| C6KSY6\|C6KSY6 | 60S ribosomal protein L19 OS |
| Q8IDS6\|Q8IDS6 | 60S ribosomal protein L18a OS |
| C0H5G3\|C0H5G3 | 60S ribosomal protein L18-2 |
| Q8ILE8\|Q8ILE8 | 60S ribosomal protein L14 |
| Q8IAX6\|Q8IAX6 | 60S ribosomal protein L13 OS |
| Q8IJZ7\|Q8IJZ7 | 60S ribosomal protein L13 |
| Q8I3T8\|Q8I3T8 | 60S ribosomal protein L12 |
| Q8IBQ6\|Q8IBQ6 | 60S ribosomal protein L11a |
| Q8ILV2\|Q8ILV2 | 60S ribosomal protein L10 |
| Q8IDI5\|Q8IDI5 | 60S ribosomal protein L1 |
| Q8IL58\|Q8IL58 | 60S ribosomal protein L1 |
| O97250\|O97250 | 60S ribosomal protein L |
| Q8I1P0\|Q8I1P0 | 60S ribosomal protein L |
| C0H5I4\|C0H5I4 | 60S ribosomal protein L |
| Q8ILL2\|Q8ILL2 | 60S ribosomal protein L |
| Q8IBG6\|Q8IBG6 | 60S ribosomal export protein NMD3 |
| Q8IIX0\|Q8IIX0 | 60S acidic ribosomal protein P1 |
| Q8II61\|Q8II61 | 60S acidic ribosomal protein P0 |
| Q8I0V3\|Q8I0V3 | 60 kDa chaperonin |
| Q8I4X1\|Q8I4X1 | 5-aminolevulinate synthase |
| Q9NLA7\|Q9NLA7 | 50S ribosomal protein L9 |
| A0A5K1K8K5\|A0A5K1K8K5 | 50S ribosomal protein L9 |
| Q8I373\|Q8I373 | 50S ribosomal protein L35 |
| C6S3B5\|C6S3B5 | 50S ribosomal protein L33 |
| C0H543\|C0H543 | 50S ribosomal protein L3 |
| Q8I4X7\|Q8I4X7 | 50S ribosomal protein L3 |
| C0H4C3\|C0H4C3 | 50S ribosomal protein L28 |
| C6KSP8\|C6KSP8 | 50S ribosomal protein L24 |
| Q8I5H8\|Q8I5H8 | 50S ribosomal protein L24 |
| Q8IJT7\|Q8IJT7 | 50S ribosomal protein L22 |
| Q8IKG3\|Q8IKG3 | 50S ribosomal protein L22 |
| Q8II37\|Q8II37 | 50S ribosomal protein L2 |
| C0H4F0\|C0H4F0 | 50S ribosomal protein L14 |
| Q8I3L9\|Q8I3L9 | 50S ribosomal protein L12 |
| Q8I1U9\|Q8I1U9 | 50S ribosomal protein L10 |
| C0H4F9\|C0H4F9 | 50S ribosomal protein L1 |
| Q8IBY1\|Q8IBY1 | 50S ribosomal protein L1 |
| Q8I5U9\|Q8I5U9 | 50S ribosomal protein L1 |
| C6KSS3\|C6KSS3 | 4-hydroxybenzoate polyprenyltransferase |
| Q8IJH7\|Q8IJH7 | 4-hydroxy-3-methylbut-2-en-1-yl diphosphate synthase (Ferredoxin) |
| Q8I470\|Q8I470 | 4-diphosphocytidyl-2-C-methyl-D-erythritol kinase |
| A0A143ZZG2\|A0A143ZZG2 | 40S ribosomal protein SA |
| Q8I3R0\|Q8I3R0 | 40S ribosomal protein S9 |
| Q8IM10\|Q8IM10 | 40S ribosomal protein S8 |
| Q8IDR9\|Q8IDR9 | 40S ribosomal protein S6 |
| Q8IL02\|Q8IL02 | 40S ribosomal protein S5 |
| Q8IBN5\|Q8IBN5 | 40S ribosomal protein S5 |
| Q8IIU8\|Q8IIU8 | 40S ribosomal protein S4 |
| A0A144A0U2\|A0A144A0U2 | 40S ribosomal protein S30 |
| Q8IKH8\|Q8IKH8 | 40S ribosomal protein S3 |
| C0H4K8\|C0H4K8 | 40S ribosomal protein S29 |
| Q8IKL9\|Q8IKL9 | 40S ribosomal protein S28 |
| O96258\|O96258 | 40S ribosomal protein S26 |
| Q8ILN8\|Q8ILN8 | 40S ribosomal protein S25 |
| Q8I3R6\|Q8I3R6 | 40S ribosomal protein S24 |
| O97248\|O97248 | 40S ribosomal protein S23 |
| Q8IHS5\|Q8IHS5 | 40S ribosomal protein S21 |
| Q8IK02\|Q8IK02 | 40S ribosomal protein S20e |
| Q8IEN2\|Q8IEN2 | 40S ribosomal protein S2 |
| Q8IFP2\|Q8IFP2 | 40S ribosomal protein S19 |
| C0H5C2\|C0H5C2 | 40S ribosomal protein S19 |
| Q8IIA2\|Q8IIA2 | 40S ribosomal protein S18 |
| Q8IAX5\|Q8IAX5 | 40S ribosomal protein S16 |
| O77395\|O77395 | 40S ribosomal protein S15A |
| Q8IDB0\|Q8IDB0 | 40S ribosomal protein S15 |
| A0A5K1K925\|A0A5K1K925 | 40S ribosomal protein S11 |
| O77381\|O77381 | 40S ribosomal protein S11 |
| Q8IBQ5\|Q8IBQ5 | 40S ribosomal protein S10 |
| Q8I502\|Q8I502 | 40S ribosomal protein S1 |
| Q8IET7\|Q8IET7 | 40S ribosomal protein S |
| Q8II05\|Q8II05 | 3-oxo-5-alpha-steroid 4-dehydrogenase |
| Q8IJM7\|Q8IJM7 | 3-hydroxyisobutyryl-CoA hydrolase |
| Q8I523\|Q8I523 | 3-hydroxyisobutyryl-CoA hydrolase |
| Q8ILW1\|Q8ILW1 | 30S ribosomal protein S9 |
| Q9U0G9\|Q9U0G9 | 30S ribosomal protein S12 |
| Q8I0X1\|Q8I0X1 | 2-oxoisovalerate dehydrogenase subunit beta |
| Q8IEJ6\|Q8IEJ6 | 2-oxoisovalerate dehydrogenase subunit alpha |
| O96145\|O96145 | 2-methoxy-6-polyprenyl-1 |
| Q8I273\|Q8I273 | 2-C-methyl-D-erythritol 4-phosphate cytidylyltransferase |
| A0A143ZWM3\|A0A143ZWM3 | 2-C-methyl-D-erythritol 2 |
| Q8IJA0\|Q8IJA0 | 26S proteasome regulatory subunit RPN9 |
| Q8I323\|Q8I323 | 26S proteasome regulatory subunit RPN8 |
| Q8IM66\|Q8IM66 | 26S proteasome regulatory subunit RPN6 |
| A0A5K1K9F3\|A0A5K1K9F3 | 26S proteasome regulatory subunit RPN3 |
| Q8IKH3\|Q8IKH3 | 26S proteasome regulatory subunit RPN2 |
| Q8ILV5\|Q8ILV5 | 26S proteasome regulatory subunit RPN13 |
| O77345\|O77345 | 26S proteasome regulatory subunit RPN12 |
| Q8ID28\|Q8ID28 | 26S proteasome regulatory subunit RPN11 |
| Q8IAR6\|Q8IAR6 | 26S proteasome regulatory subunit RPN10 |
| O96153\|O96153 | 26S proteasome regulatory subunit RPN1 |
| Q8II71\|Q8II71 | 26S proteasome regulatory subunit RPN |
| Q8IJM0\|Q8IJM0 | 26S proteasome regulatory subunit p55 |
| O77379\|O77379 | 26S proteasome non-ATPase regulatory subunit 9 |
| Q8I4U5\|Q8I4U5 | 26S protease regulatory subunit 8 |
| Q8I1V1\|Q8I1V1 | 26S protease regulatory subunit 6B |
| Q8II60\|Q8II60 | 26S protease regulatory subunit 6A |
| Q8IJW0\|Q8IJW0 | 26S protease regulatory subunit 4 |
| Q8IEQ1\|Q8IEQ1 | 26S protease regulatory subunit 10B |
| Q8IEK3\|Q8IEK3 | 26S protease regulatory subunit |
| Q8IDZ8\|Q8IDZ8 | 20 kDa chaperonin |
| Q8ILV7\|Q8ILV7 | 2-(3-amino-3-carboxypropyl)histidine synthase subunit 1 |
| Q8IDW0\|Q8IDW0 | 1-deoxy-D-xylulose-5-phosphate synthase |
| A0A144A2T4\|A0A144A2T4 | 1-deoxy-D-xylulose-5-phosphate reductoisomerase |
| O97232\|O97232 | 1-cys-glutaredoxin-like protein-1 |
| Q8IL28\|Q8IL28 | 1-acyl-sn-glycerol-3-phosphate acyltransferase |
| Q8I5K8\|Q8I5K8 | 18S rRNA aminocarboxypropyltransferase |
| C0H4F7\|C0H4F7 | 18S rRNA |
| C0H4V6\|C0H4V6 | 14-3-3 protein |
| A0A5K1K948\|A0A5K1K948 | 14-3-3 protein |
| Q8I5Q3\|Q8I5Q3 | 10 kDa chaperonin |
| Q8I6T4\|Q8I6T4 | (3R)-hydroxymyristoyl-[acyl-carrier-protein] dehydratase |

**Table S2**. *P. falciparum* proteins, non-homologous to human

| Protein ID |
| --- |
| Q8I535\|Q8I535_PLAF7 |
| C0H4F9\|C0H4F9_PLAF7 |
| C6KST9\|C6KST9_PLAF7 |
| Q8IK01\|Q8IK01_PLAF7 |
| Q8IBB3\|Q8IBB3_PLAF7 |
| O77354\|O77354_PLAF7 |
| Q8ILW7\|Q8ILW7_PLAF7 |
| Q8I599\|Q8I599_PLAF7 |
| O77375\|O77375_PLAF7 |
| Q8I257\|Q8I257_PLAF7 |
| Q8IE49\|Q8IE49_PLAF7 |
| C6KTC3\|C6KTC3_PLAF7 |
| C0H573\|C0H573_PLAF7 |
| Q8IIQ2\|Q8IIQ2_PLAF7 |
| Q8I273\|Q8I273_PLAF7 |
| Q8I3J0\|Q8I3J0_PLAF7 |
| Q8I5D3\|Q8I5D3_PLAF7 |
| Q8IM51\|Q8IM51_PLAF7 |
| A0A5K1K7X4\|A0A5K1K7X4_PLAF7 |
| Q8I0V0\|SUB1_PLAF7 |
| Q8IAX3\|Q8IAX3_PLAF7 |
| Q8II78\|Q8II78_PLAF7 |
| Q8IJU3\|Q8IJU3_PLAF7 |
| Q8I624\|Q8I624_PLAF7 |
| Q8IKD6\|Q8IKD6_PLAF7 |
| Q8IEN7\|Q8IEN7_PLAF7 |
| Q8I3G2\|Q8I3G2_PLAF7 |
| O97241\|O97241_PLAF7 |
| C0H4F3\|C0H4F3_PLAF7 |
| Q8IBL8\|Q8IBL8_PLAF7 |
| Q8IKU1\|Q8IKU1_PLAF7 |
| Q8IM19\|Q8IM19_PLAF7 |
| Q8I3H6\|Q8I3H6_PLAF7 |
| Q8IJX0\|Q8IJX0_PLAF7 |
| Q9NLA7\|Q9NLA7_PLAF7 |
| Q8IB49\|Q8IB49_PLAF7 |
| C0H5L9\|C0H5L9_PLAF7 |
| Q8ID37\|Q8ID37_PLAF7 |
| C0H5B7\|C0H5B7_PLAF7 |
| A0A143ZYR6\|A0A143ZYR6_PLAF7 |
| Q9U0H1\|Q9U0H1_PLAF7 |
| Q8I318\|Q8I318_PLAF7 |
| A0A5K1K940\|A0A5K1K940_PLAF7 |
| Q8IM27\|Q8IM27_PLAF7 |
| Q8IDG3\|Q8IDG3_PLAF7 |
| Q8IL50\|Q8IL50_PLAF7 |
| Q8IK27\|Q8IK27_PLAF7 |
| Q8ILZ6\|Q8ILZ6_PLAF7 |
| O97259\|O97259_PLAF7 |
| Q8I410\|Q8I410_PLAF7 |
| Q8I2N2\|Q8I2N2_PLAF7 |
| Q8I2M5\|Q8I2M5_PLAF7 |
| Q8I409\|Q8I409_PLAF7 |
| O96193\|O96193_PLAF7 |
| C0H5M9\|C0H5M9_PLAF7 |
| Q8IDA7\|Q8IDA7_PLAF7 |
| Q8I5L4\|Q8I5L4_PLAF7 |
| Q8I3A7\|Q8I3A7_PLAF7 |
| Q8IJZ9\|Q8IJZ9_PLAF7 |
| Q8I275\|Q8I275_PLAF7 |
| Q8IBN2\|Q8IBN2_PLAF7 |
| Q8IIN1\|Q8IIN1_PLAF7 |
| Q9NDU7\|Q9NDU7_PLAF7 |
| Q8IFN7\|Q8IFN7_PLAF7 |
| A0A144A0A4\|A0A144A0A4_PLAF7 |
| Q8I1Z2\|Q8I1Z2_PLAF7 |
| Q8ILG7\|Q8ILG7_PLAF7 |
| Q8IAM4\|Q8IAM4_PLAF7 |
| O97325\|O97325_PLAF7 |
| O97310\|O97310_PLAF7 |
| Q8IAR3\|Q8IAR3_PLAF7 |
| Q8I082\|Q8I082_PLAF7 |
| Q8IBL6\|Q8IBL6_PLAF7 |
| A0A5K1K8K5\|A0A5K1K8K5_PLAF7 |
| O96173\|O96173_PLAF7 |
| C0H557\|C0H557_PLAF7 |
| Q8I482\|Q8I482_PLAF7 |
| Q8I487\|Q8I487_PLAF7 |
| Q8ILZ5\|Q8ILZ5_PLAF7 |
| Q8I262\|Q8I262_PLAF7 |
| Q8IKN3\|Q8IKN3_PLAF7 |
| Q8I1P4\|Q8I1P4_PLAF7 |
| Q8IEL0\|Q8IEL0_PLAF7 |
| Q8IM71\|Q8IM71_PLAF7 |
| Q8IKC5\|Q8IKC5_PLAF7 |
| A0A143ZZM5\|A0A143ZZM5_PLAF7 |
| Q8IIQ1\|Q8IIQ1_PLAF7 |
| Q8IKK5\|Q8IKK5_PLAF7 |
| Q8IIU3\|Q8IIU3_PLAF7 |
| Q8ID85\|Q8ID85_PLAF7 |
| Q8I630\|Q8I630_PLAF7 |
| Q8I2R6\|Q8I2R6_PLAF7 |
| Q8IEE9\|Q8IEE9_PLAF7 |
| C6KSY3\|C6KSY3_PLAF7 |
| Q8IL19\|Q8IL19_PLAF7 |
| A0A5K1K972\|A0A5K1K972_PLAF7 |
| Q8I2X6\|Q8I2X6_PLAF7 |
| C6KTB9\|C6KTB9_PLAF7 |
| Q8IHZ8\|Q8IHZ8_PLAF7 |
| Q8IAS0\|Q8IAS0_PLAF7 |
| Q8IHR9\|Q8IHR9_PLAF7 |
| Q8IM31\|Q8IM31_PLAF7 |
| Q8I4Z1\|Q8I4Z1_PLAF7 |
| C6KSZ2\|C6KSZ2_PLAF7 |
| Q8IKA6\|Q8IKA6_PLAF7 |
| Q8IBA8\|Q8IBA8_PLAF7 |
| Q8ILY0\|Q8ILY0_PLAF7 |
| Q9U5L9\|Q9U5L9_PLAF7 |
| C0H515\|C0H515_PLAF7 |
| A0A144A2N6\|A0A144A2N6_PLAF7 |
| Q8IKT8\|Q8IKT8_PLAF7 |
| Q8IJU6\|Q8IJU6_PLAF7 |
| Q8IEP4\|Q8IEP4_PLAF7 |
| Q8ILM9\|Q8ILM9_PLAF7 |
| Q9U0H6\|Q9U0H6_PLAF7 |
| A0A5K1K8X3\|A0A5K1K8X3_PLAF7 |
| Q8ILF2\|Q8ILF2_PLAF7 |
| Q8IBK9\|Q8IBK9_PLAF7 |
| Q8I0U9\|Q8I0U9_PLAF7 |
| Q8IB44\|Q8IB44_PLAF7 |
| Q8I6U4\|Q8I6U4_PLAF7 |
| Q8IDB3\|Q8IDB3_PLAF7 |
| Q8I1Y2\|Q8I1Y2_PLAF7 |
| Q8IL60\|Q8IL60_PLAF7 |
| Q8I3A9\|Q8I3A9_PLAF7 |
| A0A5K1K8T3\|A0A5K1K8T3_PLAF7 |
| Q8I1N7\|Q8I1N7_PLAF7 |
| Q8ILX0\|Q8ILX0_PLAF7 |
| Q8IDD8\|Q8IDD8_PLAF7 |
| Q8I4X9\|Q8I4X9_PLAF7 |
| C6KSS9\|C6KSS9_PLAF7 |
| Q8I5S7\|Q8I5S7_PLAF7 |
| Q8I451\|Q8I451_PLAF7 |
| Q8IJ89\|Q8IJ89_PLAF7 |
| Q8I425\|Q8I425_PLAF7 |
| Q8ILV8\|Q8ILV8_PLAF7 |
| Q8I5T0\|Q8I5T0_PLAF7 |
| Q8IEL8\|Q8IEL8_PLAF7 |
| Q8IJI4\|Q8IJI4_PLAF7 |
| A0A144A0A3\|A0A144A0A3_PLAF7 |
| Q8I5D4\|Q8I5D4_PLAF7 |
| Q8I2S2\|Q8I2S2_PLAF7 |
| O96253\|O96253_PLAF7 |
| Q8IJB9\|Q8IJB9_PLAF7 |
| Q8IDZ2\|Q8IDZ2_PLAF7 |
| O77371\|O77371_PLAF7 |
| Q8IIT8\|Q8IIT8_PLAF7 |
| Q8IBN1\|Q8IBN1_PLAF7 |
| C0H4Y4\|C0H4Y4_PLAF7 |
| C0H5A5\|C0H5A5_PLAF7 |
| Q8I418\|Q8I418_PLAF7 |
| Q8IJW3\|Q8IJW3_PLAF7 |
| A0A5K1K9G0\|A0A5K1K9G0_PLAF7 |
| Q8I5N9\|Q8I5N9_PLAF7 |
| Q8IC38\|Q8IC38_PLAF7 |
| Q8I0U8\|Q8I0U8_PLAF7 |
| A0A143ZWQ6\|A0A143ZWQ6_PLAF7 |
| Q8IHW8\|Q8IHW8_PLAF7 |
| Q8I422\|Q8I422_PLAF7 |
| A0A5K1K8I1\|A0A5K1K8I1_PLAF7 |
| Q8I5I3\|Q8I5I3_PLAF7 |
| O96256\|O96256_PLAF7 |
| Q8I413\|Q8I413_PLAF7 |
| Q8IBV5\|Q8IBV5_PLAF7 |
| Q8I4T6\|Q8I4T6_PLAF7 |
| Q8IL67\|Q8IL67_PLAF7 |
| C6KT65\|C6KT65_PLAF7 |
| Q8IDZ6\|Q8IDZ6_PLAF7 |
| Q8IAZ0\|Q8IAZ0_PLAF7 |
| Q8I5E6\|Q8I5E6_PLAF7 |
| Q8II99\|Q8II99_PLAF7 |
| C6KSN6\|C6KSN6_PLAF7 |
| C0H539\|C0H539_PLAF7 |
| Q8IKV0\|Q8IKV0_PLAF7 |
| O77380\|O77380_PLAF7 |
| C6KTB8\|C6KTB8_PLAF7 |
| C0H4C6\|C0H4C6_PLAF7 |
| Q8II57\|Q8II57_PLAF7 |
| A0A143ZVL6\|A0A143ZVL6_PLAF7 |
| C0H4P2\|C0H4P2_PLAF7 |
| Q8IBZ6\|Q8IBZ6_PLAF7 |
| Q8IHU4\|Q8IHU4_PLAF7 |

Methyl isolimonate acetate (**1**):  ^1^H NMR (400 MHz, DMSO-*d*_6_); δ 0.86 (3H, s, Me-8), 1.03 (3H, s, Me-4β), 1.27 (3H, s, Me-4α), 1.54 (3H, s, Me-13), 1.93 (3H, s, acetate Me), 2.76 (1H, s, H-15), 3.62 (1H, d, J = 11 Hz, H-19), 3.71 (1H, d, J = 11 Hz, H-19), 3.90(3H,s,Meester), 4.13 (1H, m, H-I), 5.22 (IH, s, H-17), 6.51 (IH, s, H-22), 7.66 (1H, s, H-23), 7.72 (1H, s, H-21). DEPT-Q NMR (100 MHz, DMSO-*d*_6_): δ 16.3 (Me-8), 16.3 (C-11), 20.4 (acetate Me), 21.4 (Me-4β), 22.6 (Me-13), 26.6 (C-12), 29.8 (Me-4α),33.3 (C-9), 36.2 (C-2), 37.0 (C-6), 43.2 (C-13), 48.6 (C-5), 50.5 (Meester), 50.3 (C-10), 52.1 (C-8), 58.0 (C-15), 67.4 (C-19), 69.8 (C-14), 70.9 (C-1), 77.4 (C-17), 84.5(C-4), 110.2 (C-22), 120.0 (C-20), 141.7 (C-23),143.5 (C-21), 167.1 (C-16), 169.1 (C-3), 169.4 (Meester), 207.9 (C-7). HRESIMS *m/z* 545.2384 [M + H]^+^ (calc. for C_29_H_37_O_10_, 545.2387).

**
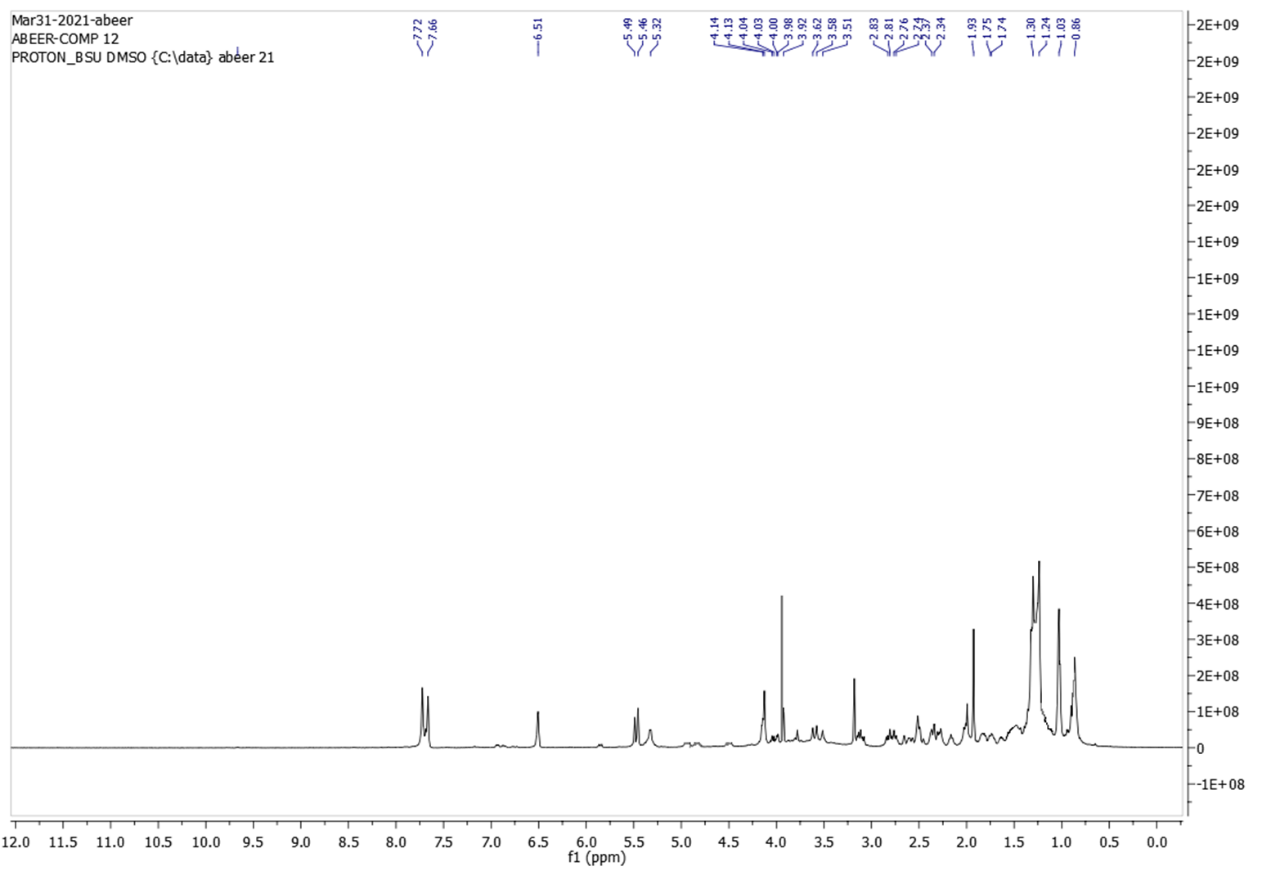
**

**Figure S1.** ^1^H NMR spectrum of compound **1** measured in DMSO-*d_6_* at 400 MHz.


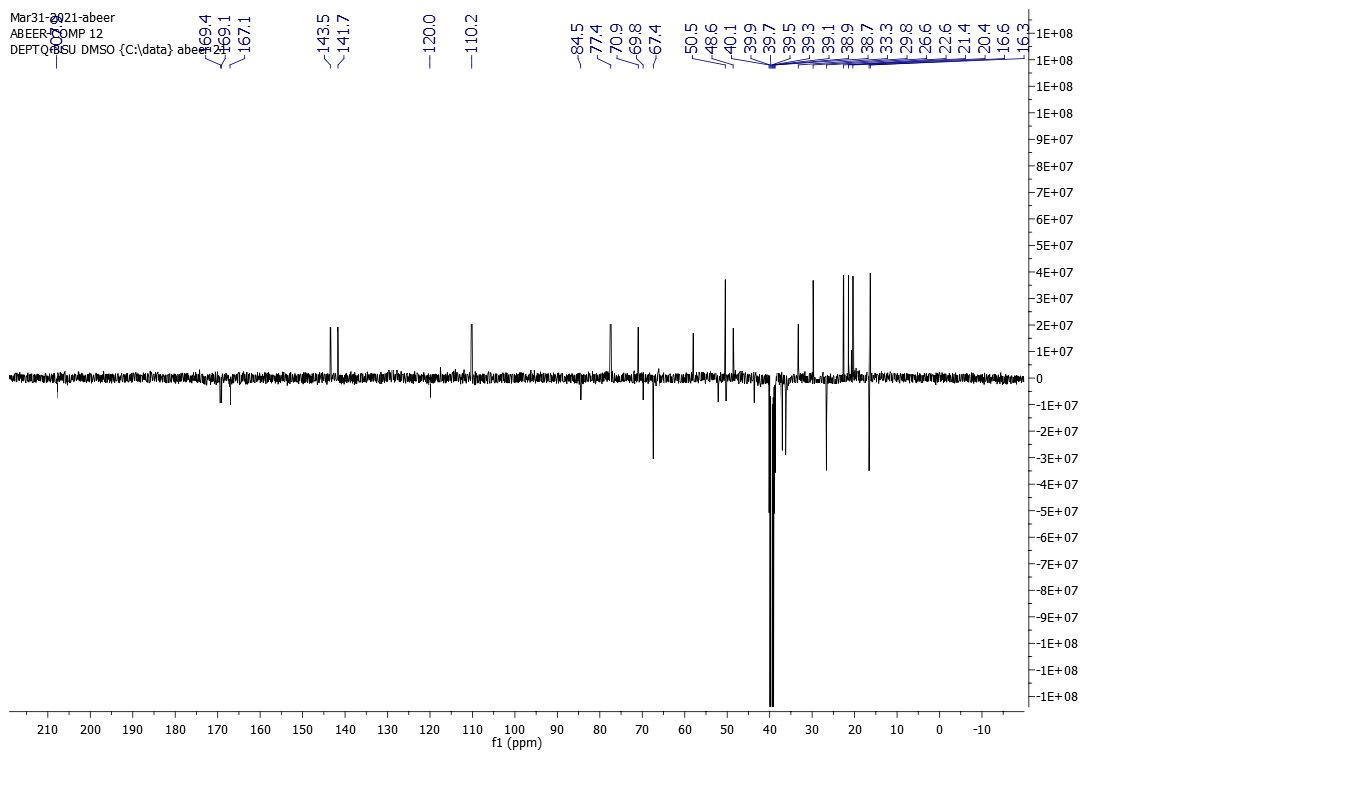


**Figure S2.** DEPT-Q NMR spectrum of compound **1** measured in DMSO-*d_6_* at 100 MHz.

Limonin (**2**): ^1^H‑NMR (400 MHz, DMSO- *d*_6_): δ (ppm) = 1.00 (s, 3H, 14-C H_3_), 1.02 (s, 3H, 22-C H_3_), 1.11 (s, 3H, 15-CH_3_), 1.19 (s, 3H, 21-C H_3_), 1.25 (m, 1H, 12-C H_2_), 1.72 (m, 2H, 12-C H_2_, 13-C H_2_), 1.82 (m, 1H, 13-H), 2.27 (dd, ^3^*J* = 3 Hz, ^3^*J* = 15 Hz, 1H, 4-C H_2_), 2.45 (dd, ^3^*J* = 3 Hz, ^3^*J* = 16 Hz, 1H, 3-H), 2.55 (dd, ^3^*J* = 3 Hz, ^3^*J* = 13 Hz, 1H, 1-H), 2.61 (dd, ^3^*J* = 4 Hz, ^3^*J* = 16 Hz, 1H, 18-C H_2_),  2.75 ((dd), 1H, 18-CH_2_), 3.12 (m, 1H, 4-C H_2_), 4.11 (m, 2H, 8-H, 17-H), 4.49 (m, 1H, 20-C H_2_), 4.90 (m, 1H, 20-CH_2_), 5.47 (s, 1 H, 10-H), 6.50 (m, 1H, 4´-H), 7.65 (t, ^3^*J* = 3 Hz, 1H, 3´-H), 7.72 (m, 1H, 2´H) . –^13^C-NMR (150 MHz, DMSO- *d*_6_): δ (ppm) = 17.0 (C14- CH_3_ ), 19.6 (C15-CH _3_), 21.4 (C22-C H_3_), 29.2 (C12), 29.7 (C21-CH _3_), 35.7 (C18), 36.2 (C4), 37.6 (C11), 45.2 (C2), 46.4 (C1), 50.2 (C6), 53.7 (C8), 57.9.4 (C3), 64.8 (C20), 66.7 (C8), 77.4 (C10), 78.4 (C17), 79.5 (C16), 110.2 (C4´), 120.3 (C1´), 141.7 (C2´), 143.3 (C3´), 167.3 (C9), 170.2 (C19), 208.0 (C5). – MS (ESI^+^): *m/z*(%) = 470 (100) [ M + H] ^+^. – El. Anal. for C _26_H_30_ O_8_, calcd. (%): C: 66.37; H: 6.43, found (%): C: 66.41, H: 6.79.


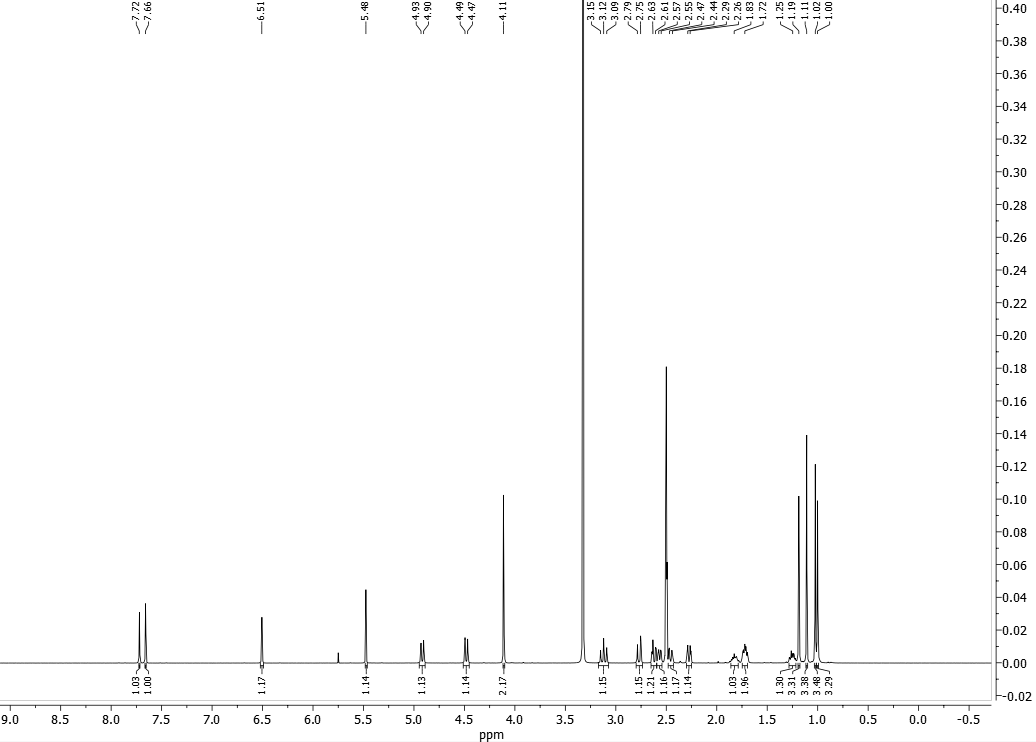


**Figure S3.** ^1^H NMR spectrum of compound **2** measured in DMSO-*d_6_* at 400 MHz.


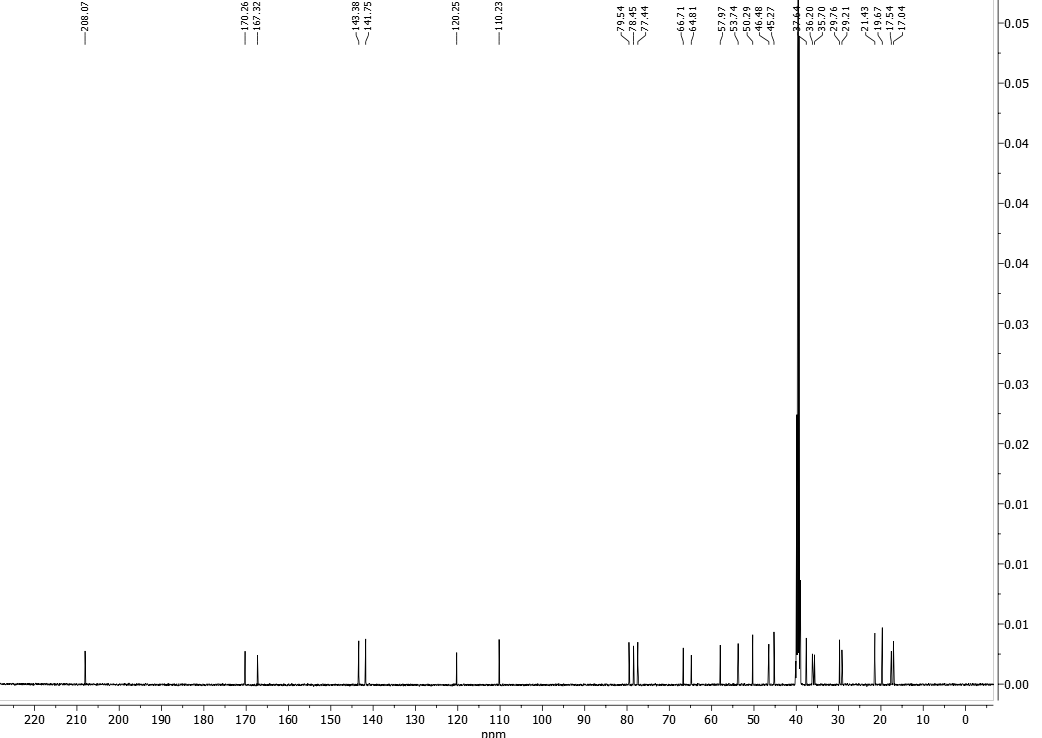


**Figure S4.** ^13^C NMR spectrum of compound **2** measured in DMSO-*d_6_* at 100 MHz.


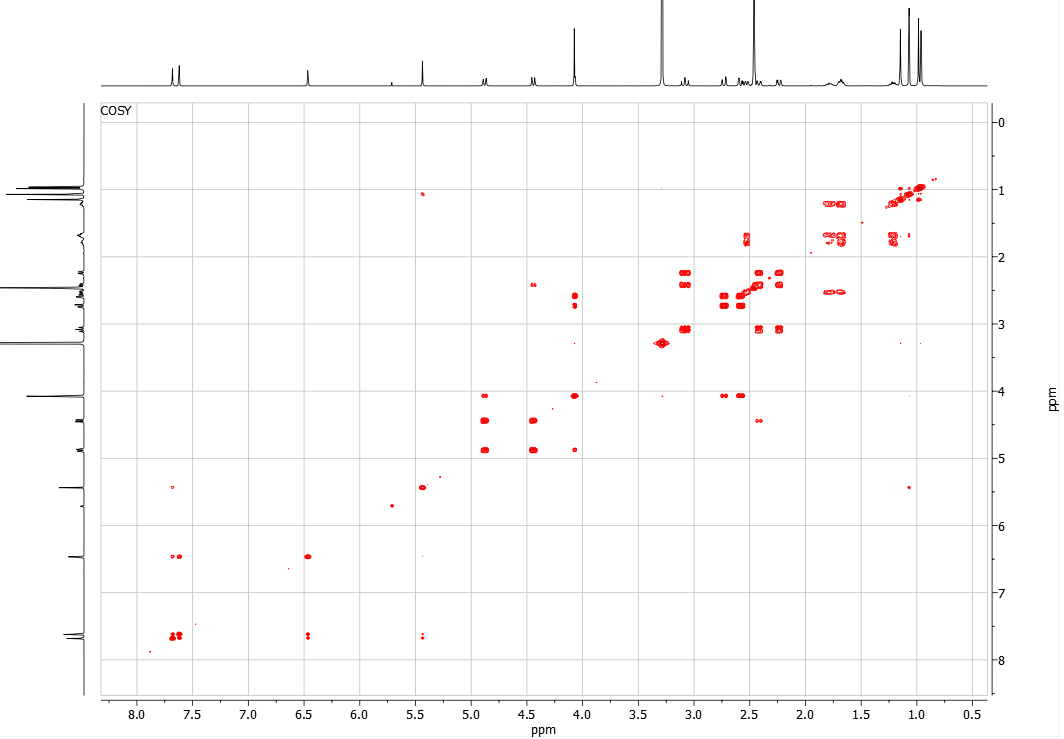


**Figure S5.** COSY NMR spectrum of compound **2** measured in DMSO-*d_6_* at 400 MHz.


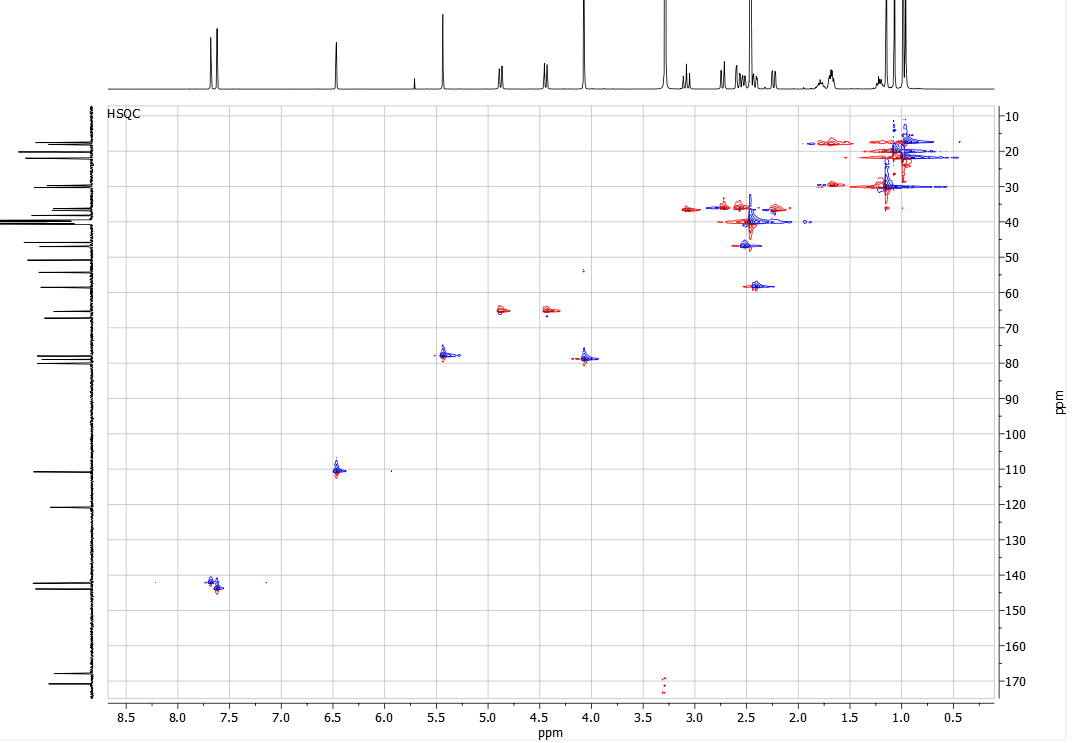


**Figure S6.** HSQC NMR spectrum of compound **2** measured in DMSO-*d_6_* at 400 MHz.


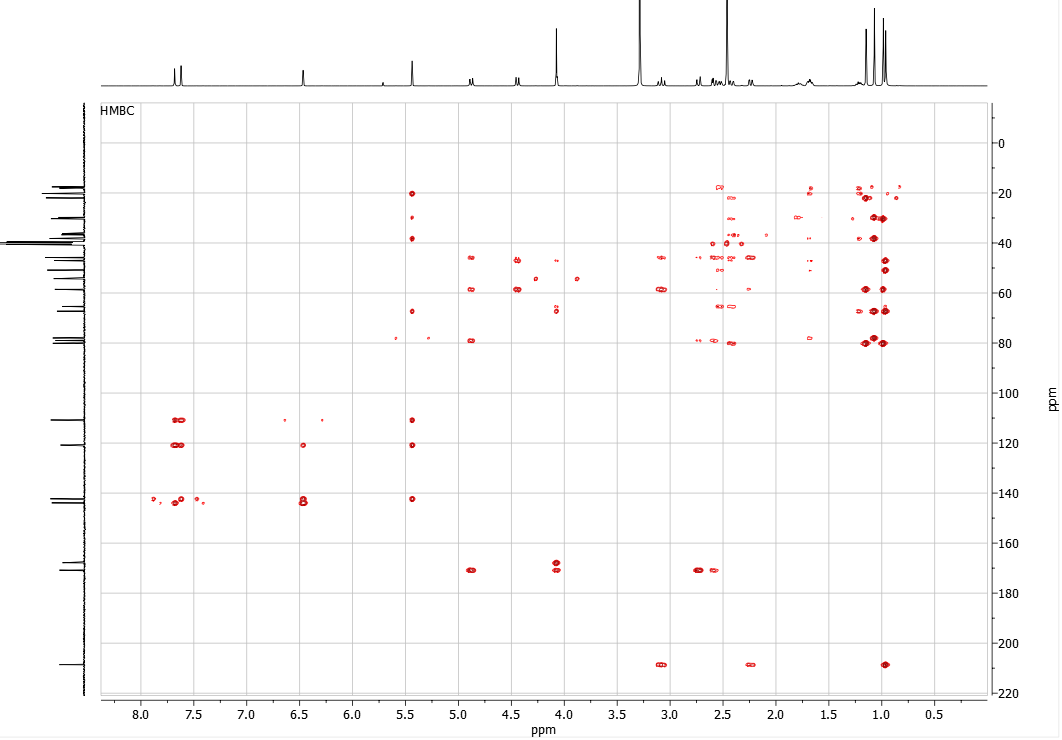


**Figure S7.** HMBC NMR spectrum of compound **2** measured in DMSO-*d_6_* at 400 MHz.

Luteolin (**3**):  ^1^H NMR (400 MHz, Chloroform) δ 6.95 (*s*, 1H, 2'-CH), 6.77 (*d*, *J* = 8 Hz, 1H, 6'-CH), 6.64 (*d*, *J* = 8 Hz, 1H, 5'-CH), 6.47 (s, 1H, 3-CH), 6.22 (s, 1H, 8-CH), 6.10 (s, 1H, 6-CH). DEPT-Q NMR (100 MHz, Chloroform): *δ*_c_ 181.7 (C-4), 164.3 (C-7), 163.4 (C-2), 160.9 (C-5), 157.6 (C-9), 149.0 (C-4'), 145.7 (C-3'), 121.4 (C-1'), 119.0 (C-6'), 116.1 (C-5'), 112.7 (C-2'), 103.6 (C-10), 103.1 (C-3), 98.6 (C-6), 94.4 (C-8).


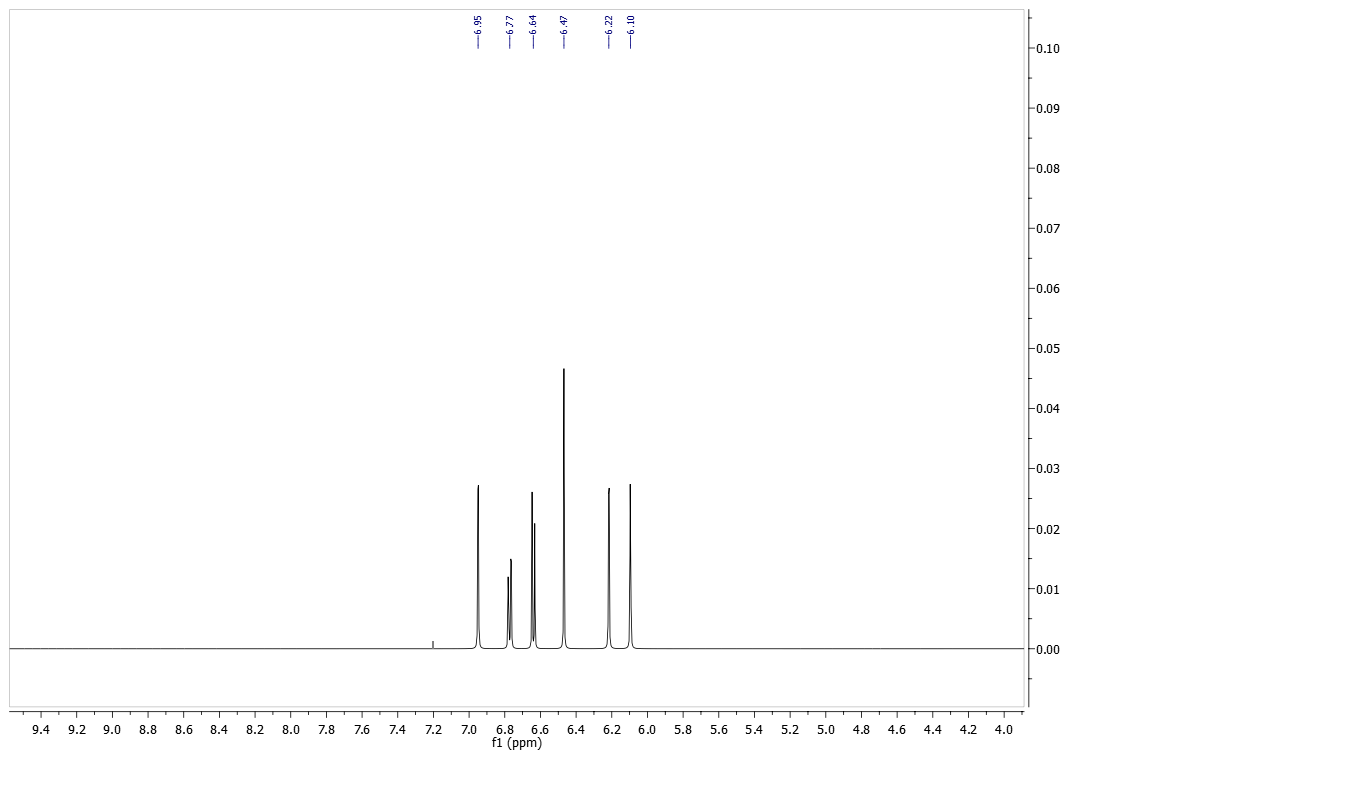


**Figure S8.** 1H NMR spectrum of compound **3** measured in CDCl_3_-*d* at 400 MHz.


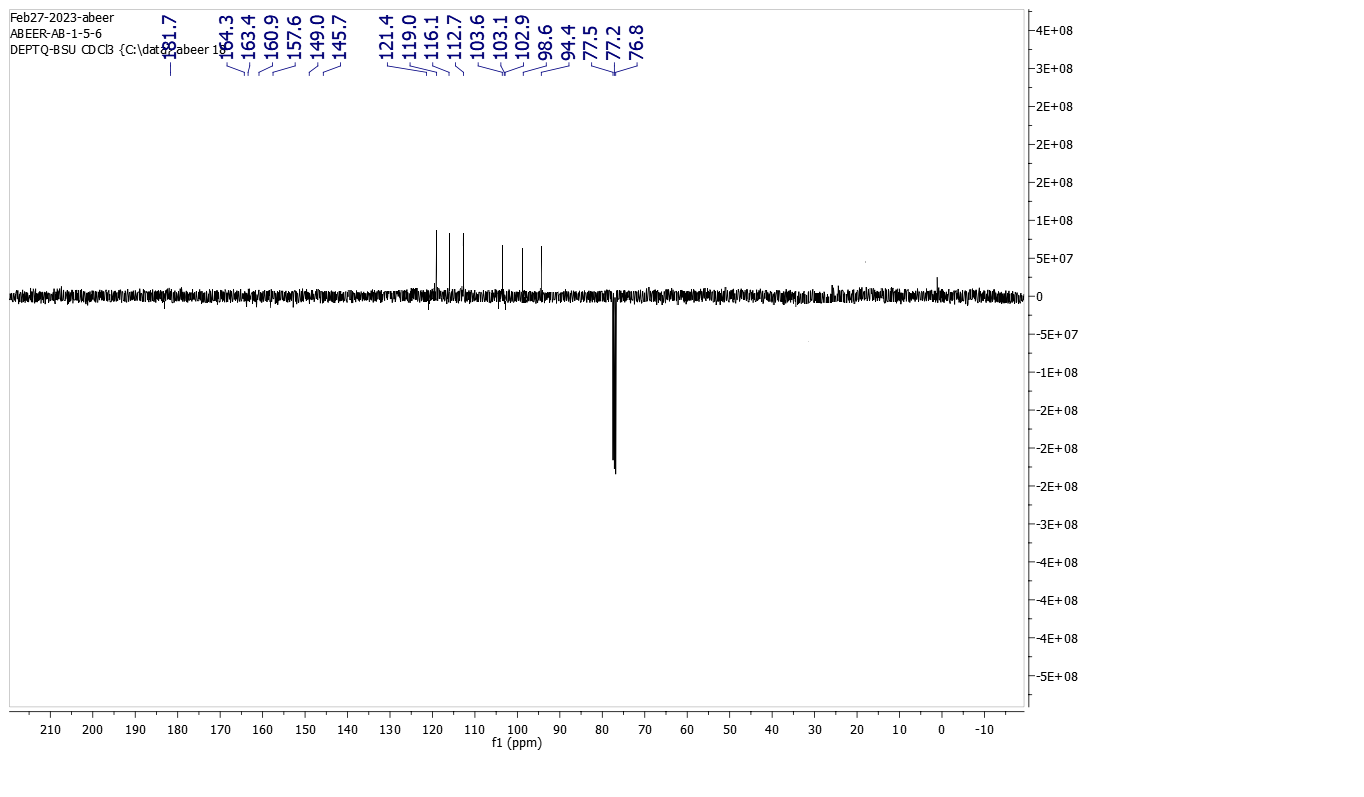


**Figure S9.** ^13^C NMR spectrum of compound **3** measured in CDCl_3_-*d* at 100 MHz.

3'-Hydroxy-genkwanin (**4**):  ^1^H NMR (400 MHz, Chloroform) δ 7.01 (*d*, *J* = 8 Hz, 1H, 6'-CH), 6.98 (*s*, 1H, 2'-CH), 6.72 (*d*, *J* = 8 Hz, 1H, 5'-CH), 6.55 (s, 1H, 3-CH), 6.31 (s, 1H, 8-CH), 6.16 (s, 1H, 6-CH), 3.82 (s, 3H, 7-OCH_3_). DEPT-Q NMR (100 MHz, Chloroform): *δ*_c_ 182.2 (C-4), 164.3 (C-7), 164.1 (C-2), 161.0 (C-5), 157.1 (C-9), 152.1 (C-4'), 148.9 (C-3'), 122.1 (C-1'), 120.6 (C-6'), 116.4 (C-5'), 110.9 (C-2'), 104.0 (C-10), 103.6 (C-3), 99.2 (C-6), 94.4 (C-8), 55.9 (-OCH_3_).


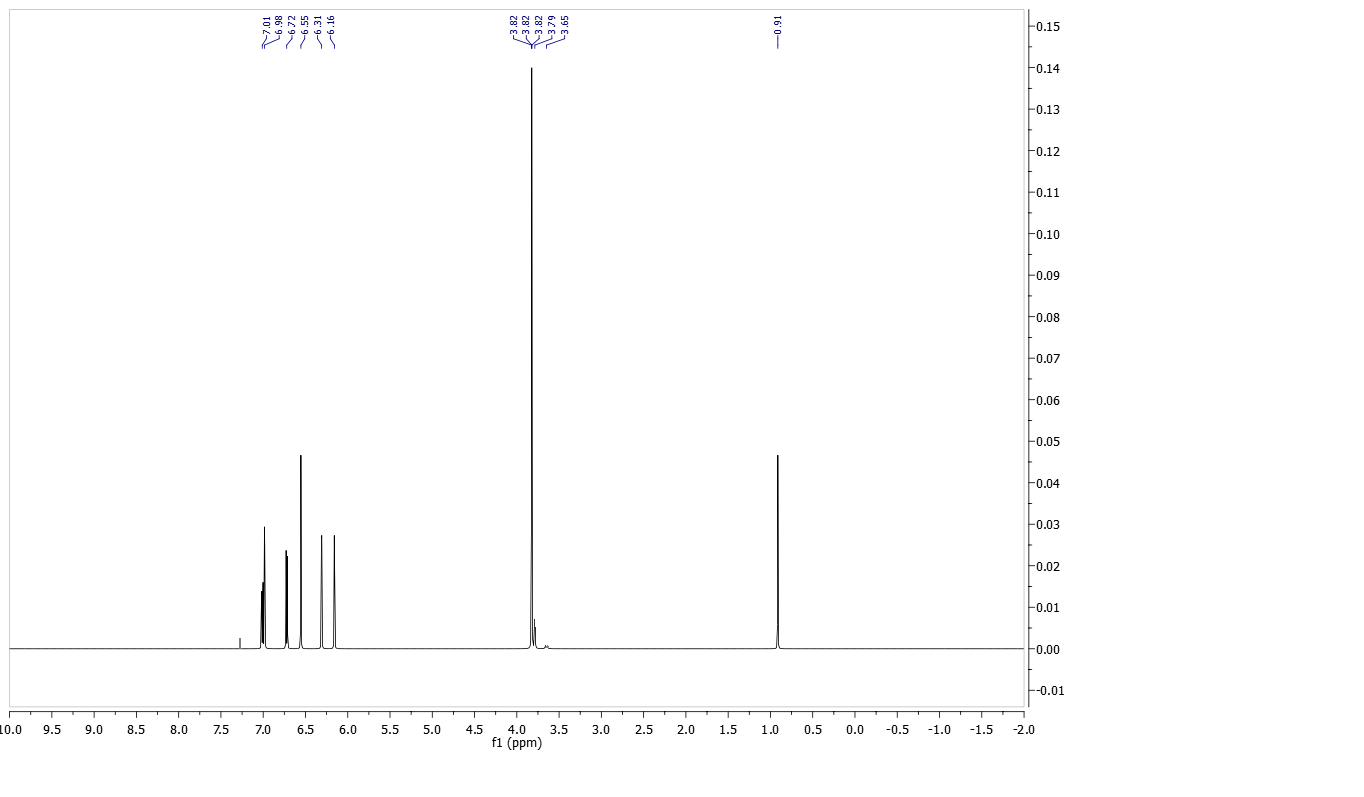


**Figure S10.** ^1^H NMR spectrum of compound **4** measured in CDCl_3_-*d* at 400 MHz.


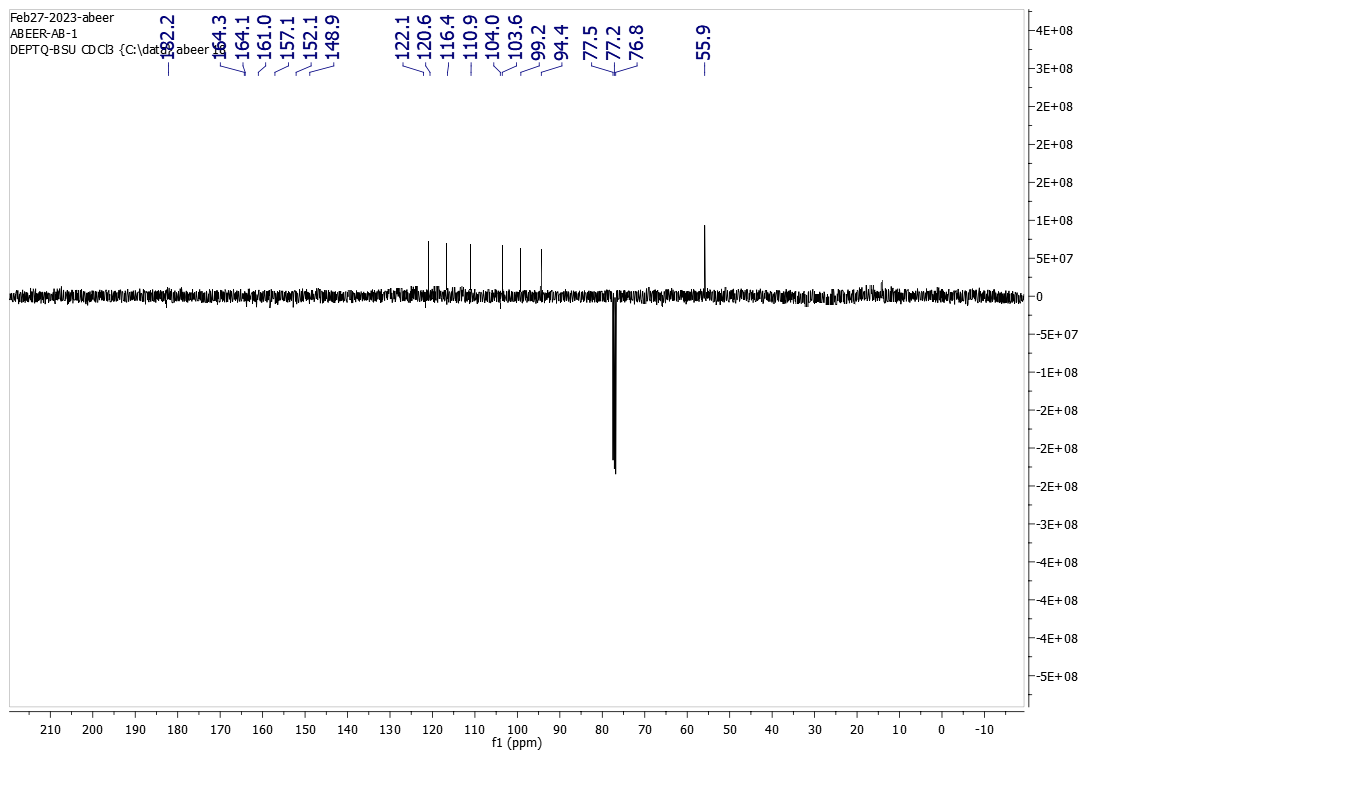


**Figure S11.** ^13^C NMR spectrum of compound **4** measured in CDCl_3_-*d* at 100 MHz.

Myricetin (**5**): ^1^H NMR (400 MHz, Chloroform) δ 6.47 (*s*, 2H, 2', 6'-CH), 6.21 (s, 1H, 8-CH), 6.10 (s, 1H, 6-CH). DEPT-Q NMR (100 MHz, Chloroform): *δ*_c_ 175.8 (C-4), 164.5 (C-7), 161.0 (C-5), 157.1 (C-9), 146.2 (C-2), 145.6 (C-3′, 5′), 135.8 (C-3), 135.1 (C-4′), 120.3 (C-1′), 107.0 (C-2′, 6′), 102.6 (C-10), 98.4 (C-6), 92.8 (C-8).


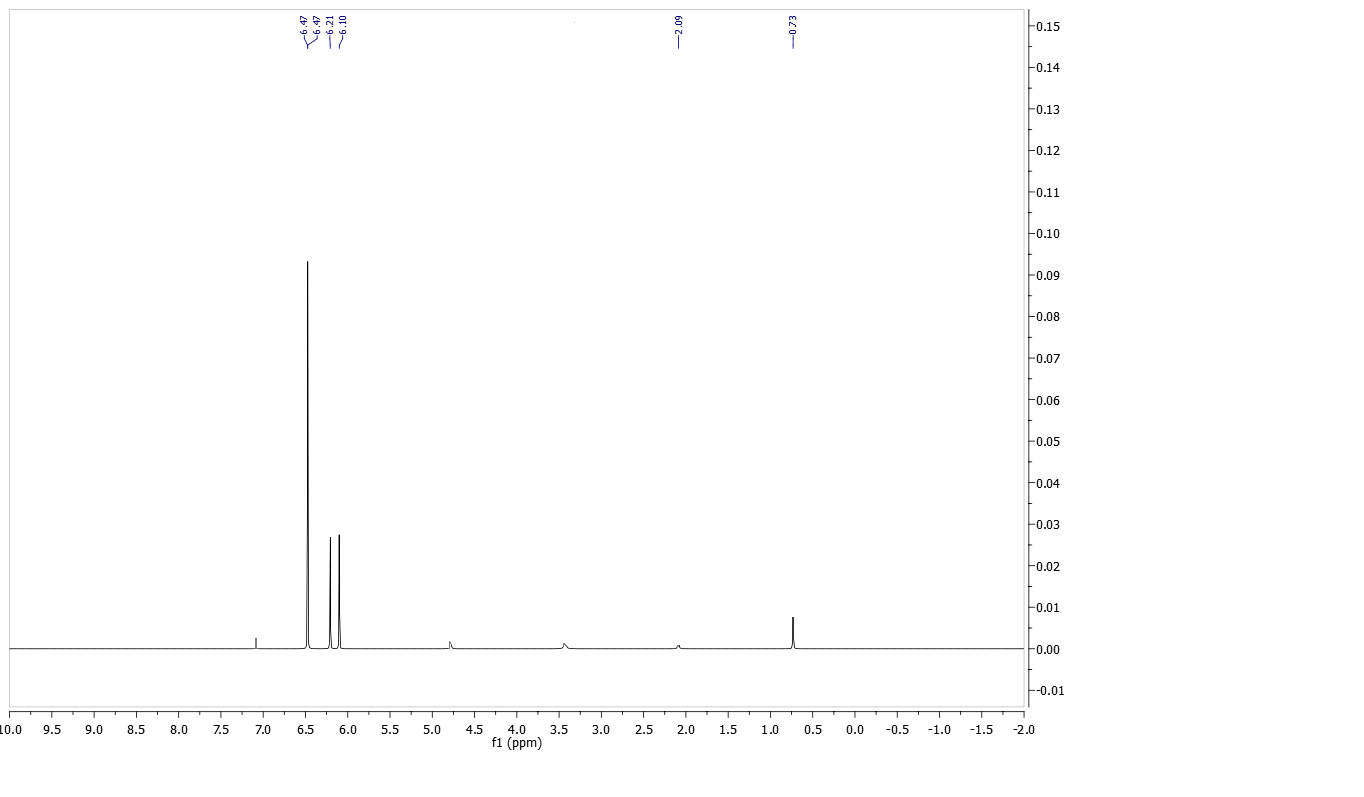


**Figure S12.** ^1^H NMR spectrum of compound **5** measured in CDCl_3_-*d* at 400 MHz.


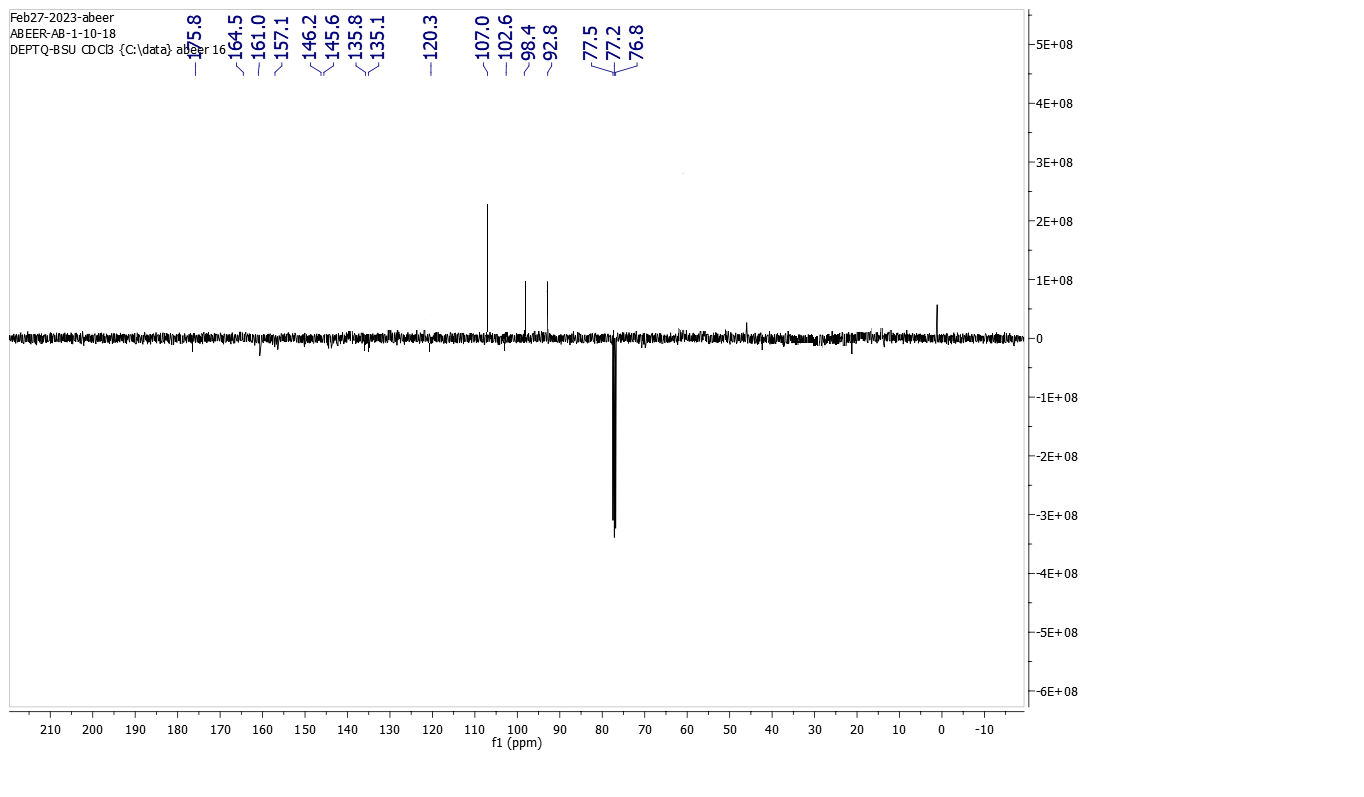


**Figure S13.** ^13^C NMR spectrum of compound **5** measured in CDCl_3_-*d* at 100 MHz.

Europetin (**6**):  ^1^H NMR (400 MHz, Chloroform) δ 6.41 (*s*, 2H, 2', 6'-CH), 6.29 (s, 1H, 8-CH), 6.22 (s, 1H, 6-CH), 3.82 (s, 3H, 7-OCH_3_). DEPT-Q NMR (100 MHz, Chloroform): *δ*_c_ 179.3 (C-4), 164.5 (C-7), 161.0 (C-5), 157.1 (C-9), 146.2 (C-2), 145.6 (C-3′, 5′), 135.8 (C-3), 135.1 (C-4′), 120.3 (C-1′), 105.3 (C-2′, 6′), 102.6 (C-10), 97.8 (C-6), 92.8 (C-8), 56.1 (-OCH_3_).


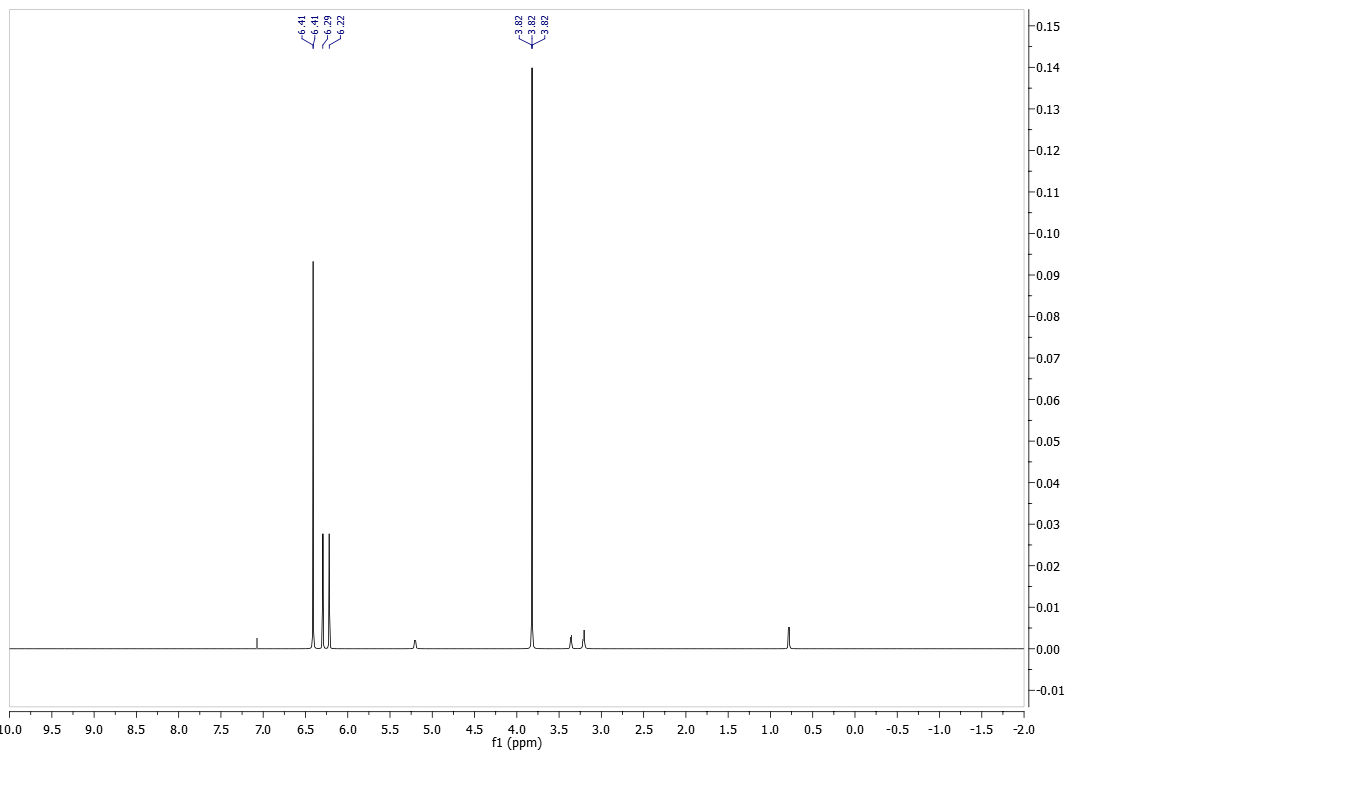


**Figure S14.** ^1^H NMR spectrum of compound **6** measured in CDCl_3_-*d* at 400 MHz.


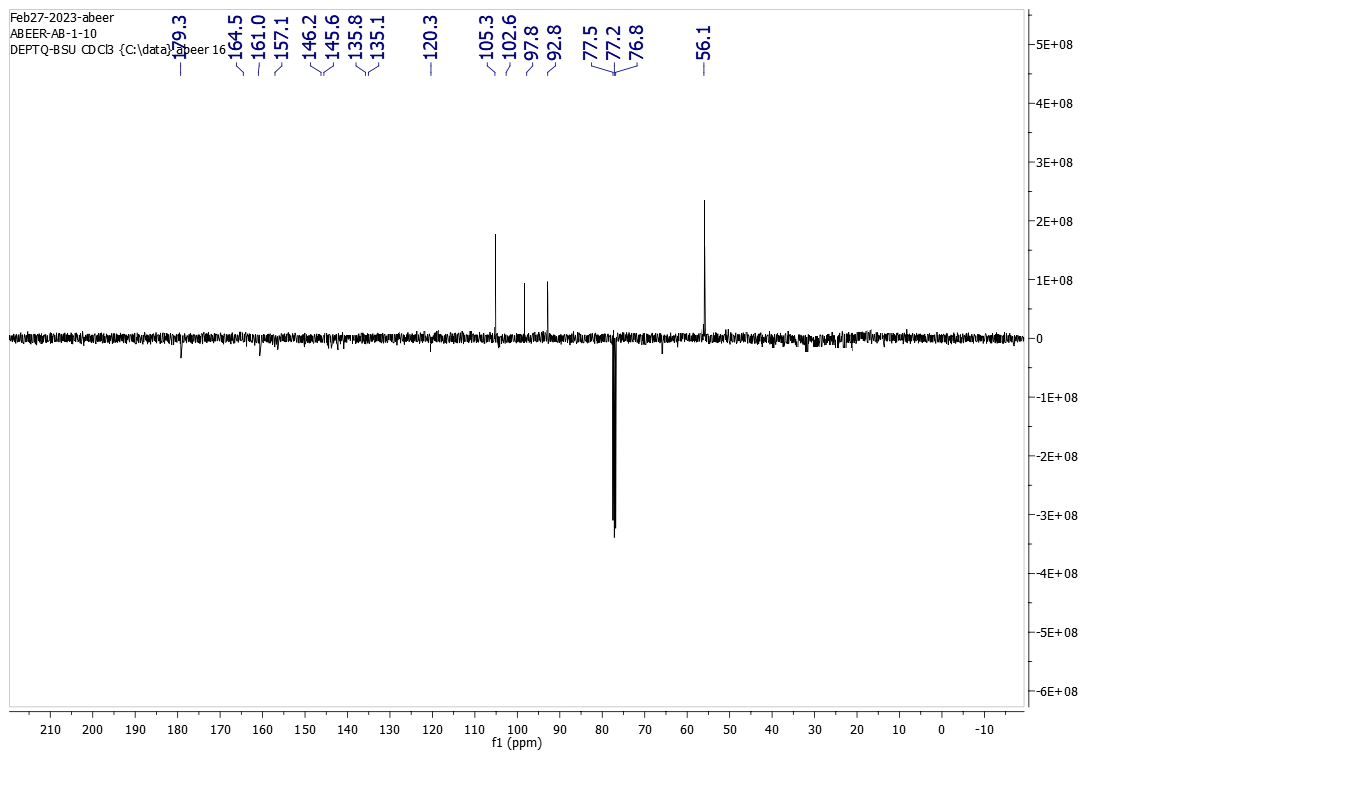


**Figure S15.** ^13^C NMR spectrum of compound **6** measured in CDCl_3_-*d* at 100 MHz.

**References**

1. Wang, X.; Shen, Y.; Wang, S.; Li, S.; Zhang, W.; Liu, X.; Lai, L.; Pei, J.; Li, H. PharmMapper 2017 update: a web server for potential drug target identification with a comprehensive target pharmacophore database. *Nucleic acids research* **2017**, *45*, W356-W360.

2. Huey, R.; Morris, G.M.; Forli, S. Using AutoDock 4 and AutoDock vina with AutoDockTools: a tutorial. *The Scripps Research Institute Molecular Graphics Laboratory* **2012**, *10550*, 1000.

3. Phillips, J.C.; Braun, R.; Wang, W.; Gumbart, J.; Tajkhorshid, E.; Villa, E.; Chipot, C.; Skeel, R.D.; Kale, L.; Schulten, K. Scalable molecular dynamics with NAMD. *Journal of computational chemistry* **2005**, *26*, 1781-1802.

4. Yuan, S.; Chan, H.S.; Hu, Z. Using PyMOL as a platform for computational drug design. *Wiley Interdisciplinary Reviews: Computational Molecular Science* **2017**, *7*, e1298.

5. Ribeiro, J.V.; Bernardi, R.C.; Rudack, T.; Schulten, K.; Tajkhorshid, E. QwikMD-Gateway for Easy Simulation with VMD and NAMD. *Biophysical Journal* **2018**, *114*, 673a-674a.

6. Humphrey, W.; Dalke, A.; Schulten, K. VMD: visual molecular dynamics. *Journal of molecular graphics* **1996**, *14*, 33-38.

7. Miller III, B.R.; McGee Jr, T.D.; Swails, J.M.; Homeyer, N.; Gohlke, H.; Roitberg, A.E. MMPBSA. py: an efficient program for end-state free energy calculations. *Journal of chemical theory and computation* **2012**, *8*, 3314-3321.

8. Mering, C.v.; Huynen, M.; Jaeggi, D.; Schmidt, S.; Bork, P.; Snel, B. STRING: a database of predicted functional associations between proteins. *Nucleic acids research* **2003**, *31*, 258-261.

9. Doncheva, N.T.; Morris, J.H.; Gorodkin, J.; Jensen, L.J. Cytoscape StringApp: network analysis and visualization of proteomics data. *Journal of proteome research* **2018**, *18*, 623-632.
